# Unequivocal determination of caulamidines A and B: application and validation of new tools in the structure elucidation tool box†
†Electronic supplementary information (ESI) available: Sample isolation, NMR experiments including RDC and RCSA analyses, ECD data, CASE results, and DFT computational methods. See DOI: 10.1039/c7sc01996c


**DOI:** 10.1039/c7sc01996c

**Published:** 2017-11-06

**Authors:** Dennis J. Milanowski, Naoya Oku, Laura K. Cartner, Heidi R. Bokesch, R. Thomas Williamson, Josep Saurí, Yizhou Liu, Kirill A. Blinov, Yuanqing Ding, Xing-Cong Li, Daneel Ferreira, Larry A. Walker, Shabana Khan, Michael T. Davies-Coleman, James A. Kelley, James B. McMahon, Gary E. Martin, Kirk R. Gustafson

**Affiliations:** a Molecular Targets Laboratory , Center for Cancer Research , National Cancer Institute , Frederick , Maryland 21702-1201 , USA . Email: gustafki@mail.nih.gov; b Basic Science Program, Leidos Biomedical Research, Inc. , Frederick National Laboratory for Cancer Research , Frederick , Maryland 21702-1201 , USA; c Structure Elucidation Group, Process and Analytical Research and Development , Merck & Co. Inc. , Rahway , New Jersey 07065 , USA . Email: gary.martin2@merck.com; d Molecule Apps , LLC , Corvallis , Oregon 97330 , USA; e National Center for Natural Products Research , Department of BioMolecular Sciences , Division of Pharmacognosy , School of Pharmacy , University of Mississippi , Oxford , Mississippi 38655 , USA; f Department of Chemistry , Rhodes University , Grahamstown , South Africa; g Chemical Biology Laboratory , Center for Cancer Research , National Cancer Institute , Frederick , Maryland 21702-1201 , USA

## Abstract

Ambiguities and errors in the structural assignment of organic molecules hinder both drug discovery and total synthesis efforts. Newly described NMR experimental approaches can provide valuable structural details and a complementary means of structure verification. The caulamidines are trihalogenated alkaloids from a marine bryozoan with an unprecedented structural scaffold. Their unique carbon and nitrogen framework was deduced by conventional NMR methods supplemented by new experiments that define 2-bond heteronuclear connectivities, reveal very long-range connectivity data, or visualize the ^35,37^Cl isotopic effect on chlorinated carbons. Computer-assisted structural elucidation (CASE) analysis of the spectroscopic data for caulamidine A provided only one viable structural alternative. Anisotropic NMR parameters, specifically residual dipolar coupling and residual chemical shift anisotropy data, were measured for caulamidine A and compared to DFT-calculated values for the proposed structure, the CASE-derived alternative structure, and two energetically feasible stereoisomers. Anisotropy-based NMR experiments provide a global, orthogonal means to verify complex structures free from investigator bias. The anisotropic NMR data were fully consistent with the assigned structure and configuration of caulamidine A. Caulamidine B has the same heterocyclic scaffold as A but a different composition and pattern of halogen substitution. Caulamidines A and B inhibited both wild-type and drug-resistant strains of the malaria parasite *Plasmodium falciparum* at low micromolar concentrations, yet were nontoxic to human cells.

## Abstract 2

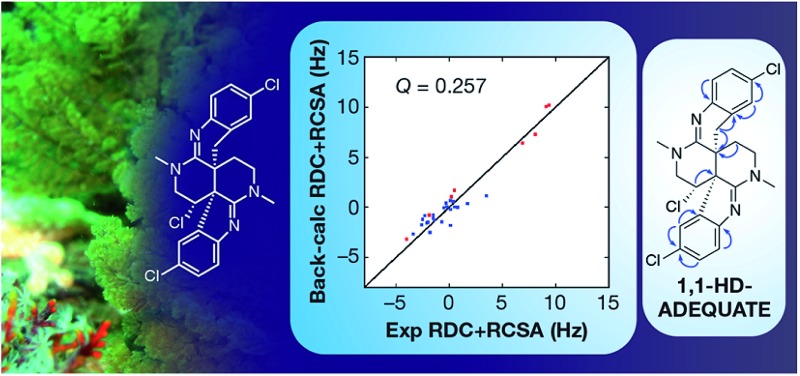
Newly described NMR experimental approaches can provide valuable structural details and a complementary means of structure verification.

## Introduction

Intricate structures of secondary metabolites evolved to interact with important cellular biopolymers, consequently they represent prevalidated templates for exploring biologically relevant chemical space. Many approved drugs are based on natural product structures and these compounds continue to provide effective scaffolds for drug development.^1^–^4^ A recent resurgence in natural products discovery and development has been driven by factors that include better bioactivity screening outcomes,^5^–^7^ advances in DNA sequencing and bioinformatics that facilitate biosynthetic engineering and prediction of the resulting chemical structures,^8^,^9^ and innovative applications of mass spectrometry and molecular networking that afford new avenues for natural product discovery.^10^–^14^ Regardless of the approach utilized, successful natural product studies require the ability to define the precise molecular structures of the isolated compounds. This is especially critical for potential drug development applications or when deduced natural product structures are the target of organic synthesis efforts. Since NMR is the most powerful and information-rich spectroscopic technique for assigning the structure of non-crystalline compounds, some recently described NMR experimental methods that facilitate the elucidation of ever more complex organic structures provide new opportunities to advance natural product discovery, development, and synthesis.

**Fig. 1:**
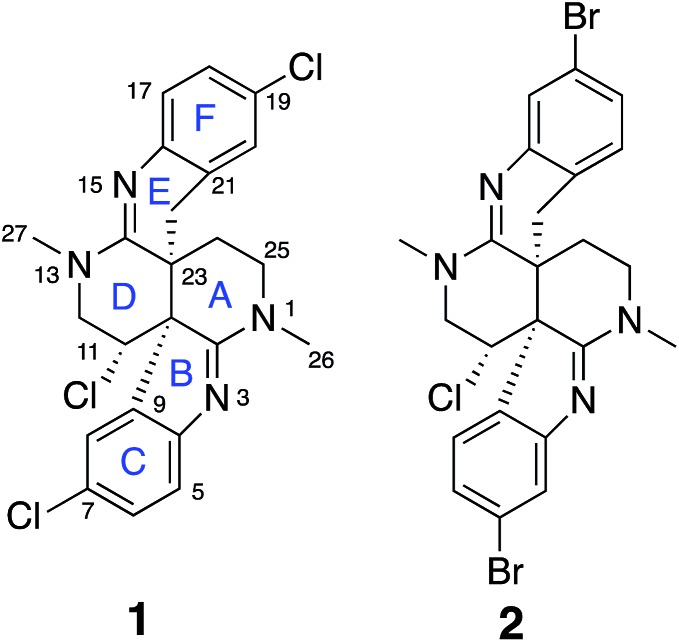
Structures of caulamidines A (**1**) and B (**2**).

A suite of standard 2D NMR experiments have been routinely used to characterize organic structures of natural and synthetic origin for more than two decades. These include HSQC and HMBC heteronuclear correlation pulse sequences to establish interatomic connectivities, COSY and TOCSY to define proton spin systems, and NOESY and ROESY to probe spatial proximity relationships. However, these well-established NMR experimental approaches can sometimes be insufficient to definitively establish the intricate skeletal frameworks and functional group assemblages encountered in many natural products. Incomplete spectroscopic characterization or interpretational mistakes can lead to ambiguous or incorrect structural assignments, as highlighted in some recent reviews.^15^,^16^ Several recently reported NMR techniques that can extend the range of heteronuclear correlations, or establish the number of bonds defined by these correlations, provide additional means to elaborate complex chemical structures.^17^–^24^ These new experimental capabilities can be especially useful for characterizing highly proton-deficient compounds. Other NMR methodologies that are applicable to chlorinated metabolites provide direct visualization of 1-bond chlorine isotope effects on carbons to define sites of Cl-substitution.^25^–^27^ In addition, anisotropy-based NMR experiments provide alternative means to evaluate the global correctness of molecular frameworks and configurations that are complementary to traditional NMR data interpretation.^17^,^28^ These data provide a robust means to verify structural assignments in an objective manner that is free of potential investigator bias. In the current study, all of these contemporary structure elucidation strategies were applied in a concerted fashion to unambiguously establish the structures of two novel heterocyclic marine alkaloids, caulamidines A (**1**) and B (**2**) (Fig. 1).

## Results and discussion

**Fig. 2:**
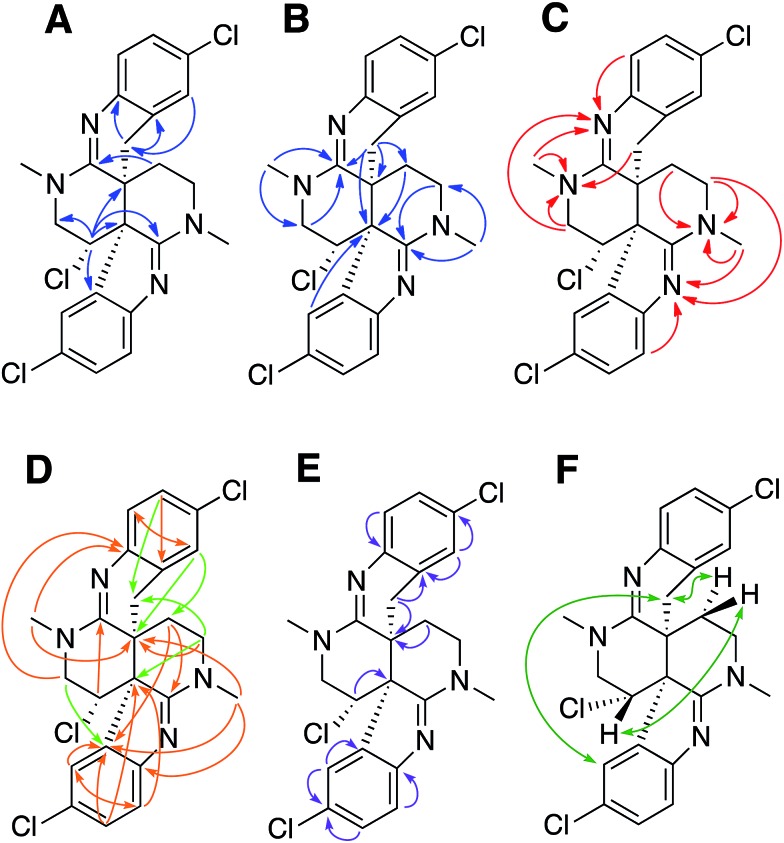
(A) and (B) Selected ^1^H–^13^C HMBC correlations for caulamidine A (**1**). (C) ^1^H–^15^N HMBC correlations. (D) Additional correlations in LR-HSQMBC with respect to ^1^H–^13^C HMBC (orange arrows) and additional correlations in HSQMBC-TOCSY with respect to LR-HSQMBC and HMBC (green arrows). (E) Key 1,1-HD-ADEQUATE correlations revealed quaternary carbons adjacent to protonated centers. (F) NOESY and ROESY correlations used to assign the relative configuration.

Previous studies of an extract from the bryozoan *Caulibugula intermis* that were conducted in the early 2000's provided the caulibugulones, a series of isoquinoline quinones and iminoquinones, as the principal cytotoxic agents.^29^ During final C_18_ HPLC purification of the caulibugulones two structurally distinct minor constituents, named caulamidines A (**1**) and B (**2**), were also obtained. Spectroscopic characterization of these compounds using the instrumentation and experimental techniques available at that time permitted the proposal of a tentative structure for caulamidine A (**1**), but convincing proof of the structure was lacking. Caulamidine B (**2**) was only isolated as a trace constituent, and while it was clearly related to **1**, we were unable to assign its structure. Decomposition of **1** while converting between free base and salt forms of the alkaloid stalled our structural studies until a recent recollection of the bryozoan provided an additional supply of compounds **1** and **2**. A characteristic 27:27:9:1 A + 2 pattern for the isotopic profile of [M + H]^+^ in the mass spectrum of **1** revealed the presence of three chlorine atoms, and HRFABMS established a molecular formula of C_23_H_21_Cl_3_N_4_, which required 14 indices of hydrogen deficiency. Extensive NMR analyses (Table 1) allowed assignment of a substituted hexahydro-2,6-naphthyridine ring system in **1** (rings A and D) with two *N*-methyl groups (*δ*_H/C_ 3.00/37.2 and 3.24/35.8). ^1^H–^13^C HMBC correlations from the N-1 methyl group to a carbon signal at *δ* 174.0 indicated an adjacent C-2 exocyclic imine functionality, while a correlation to a signal at *δ* 47.4 (C-25) established the presence of a neighboring methylene group (Fig. 2A and B). The C-25 methylene protons (*δ*_H_ 3.18, 3.38) showed COSY correlations to a second methylene group (*δ*_H_ 1.73, 2.25) and these C-24 protons exhibited HMBC cross-peaks to the two quaternary ring-junction carbons, C-10 (*δ*_C_ 58.9) and C-23 (*δ*_C_ 39.8). In a similar manner, the N-13 methyl group showed HMBC correlations to the flanking exocyclic imine (*δ*_C_ 159.1) and methylene (*δ*_C_ 52.6) carbon signals. The disparity in the ^13^C NMR shifts of the two exocyclic imine resonances suggested these functionalities were associated with different molecular scaffolds. The C-12 methylene protons (*δ*_H_ 3.66, 3.87) were coupled to the C-11 methine proton, which showed HMBC correlations to C-2, C-10, and C-23. The deshielded chemical shift of H-11 (*δ*_H_ 5.02) and C-11 (*δ*_C_ 54.8) suggested a chlorine substituent at C-11. The N-1, C-2, N-3, C-10 constellation constitutes an amidine functionality in **1**, as does N-13, C-14, N-15, and C-23.

**Table 1:** NMR spectroscopic data for caulamidine A (**1**) in CD_3_CN

Position	*δ*_C_	*δ*_N_ ^*a*^	*δ*_H_ (mult, *J* in Hz)	HMBC^*b*^
1-N	—	78.9	—	—
2	174.0	—		—
3-N	—	241.7	—	—
4	156.0	—	—	—
5	117.8	—	7.17 (d, 8.5)	3, 4, 6, 7, 9
6	129.4	—	7.31 (dd, 8.4, 2.0)	4, 5, 7, 8
7	126.3	—	—	—
8	123.8	—	6.95 (bs)	4, 6, 7, 10
9	133.3	—	—	—
10	58.9	—	—	—
11	54.8	—	5.02 (dd, 10.8, 4.7)	2, 9, 10, 12, 23
12a	52.6	—	3.87 (dd, 13.3, 6.6)	11, 13, 14, 15, 27
12b			3.66 (dd, 13.3, 10.5)	11, 13, 14, 15
13-N	—	87.5	—	—
14	159.1	—	—	—
15-N	—	216.6	—	—
16	143.9	—	—	—
17	124.2	—	6.94 (d, 8.2)	15, 16, 19, 21
18	127.2	—	7.12 (dd, 8.2, 2.4)	16, 19
19	125.8	—	—	—
20	127.3	—	6.96 (s)	16, 18, 19, 21, 22
21	125.4	—	—	—
22a	29.6	—	2.48 (d, 15.9)	10, 14, 16, 21, 23, 24
22b			2.28 (d, 15.9)	10, 13, 14, 16, 21, 23, 24
23	39.8	—	—	—
24a	24.7	—	2.25 (m)	10, 14, 22, 23, 25
24b			1.73 (dd, 15.0, 6.2)	1, 10, 22, 23, 25
25a	47.4	—	3.38 (ddd, 12.5, 7.5, 1.6)	2, 3, 24
25b			3.18 (dt, 11.7, 5.9)	24, 26
26	37.2	—	3.00, 3H (s)	1, 2, 3, 25
27	35.8	—	3.24, 3H (s)	12, 13, 14, 15

^*a*^ ^15^N assignments were based on ^1^H–^15^N HMBC correlations. The *δ*_N_ values were not calibrated to an external standard but were referenced to neat NH_3_ (*δ* 0.00) using the standard Bruker parameters.

^*b*^ ^1^H–^13^C (optimized for 8.3 Hz) and ^1^H–^15^N (optimized for 8 Hz) HMBC correlations are listed.

Carbon and proton resonances for two 1,2,4-trisubstituted benzene moieties in **1** were readily assigned. *ortho*-Coupling (8.4 Hz) between H-5 and H-6 and *meta*-coupling (2.0 Hz) between H-6 and H-8 defined the proton distribution in the C-ring, while nitrogen-substitution at C-4 was based on its deshielded chemical shift (*δ*_C_ 156.0). The link between C-9 and the C-10 bridgehead was based on an HMBC correlation between H-8 and C-10, which established the presence of a fused pyrrole moiety (B). Tentative assignment of a chlorine substituent at C-7 was consistent with its chemical shift of *δ*_C_ 126.3. The second benzene moiety (F) had a similar distribution of protons as defined by their coupling patterns, while the presence of a nitrogen at C-16 (*δ*_C_ 143.9), and a chlorine substituent at C-19 (*δ*_C_ 125.8) was proposed based on their chemical shift values. Attachment of a methylene group attached at C-21 was evident from HMBC correlations from H-20 (*δ*_H_ 6.96) to C-22 (*δ*_C_ 29.6), and conversely from the H-22 protons (*δ*_H_ 2.28 and 2.48) to C-16 (*δ*_C_ 143.9) and C-21 (*δ*_C_ 125.4). The isolated H-22 methylene protons had numerous further HMBC correlations into rings A and D that established its connection to the C-23 bridgehead. Thus, both N-15 and C-22 were incorporated into a fused 6-membered ring (E) situated between rings D and F. Establishment of the 5- and 6-membered rings containing the C-2 and C-14 imino carbons, respectively, was consistent with the disparity observed for the DFT-calculated ^13^C shifts for the dihydroindole-derived (C-2, calculated *δ*_C_ 173.8) and tetrahydroquinoline-derived (C-14, calculated *δ*_C_ 156.8) systems that are fused to the 2,6-naphthyridine core of **1**. The deshielded chemical shifts of B-ring carbons compared with corresponding E-ring signals were attributed to the ring strain associated with the configuration of the fused 5-membered B-ring. An extensive set of ^1^H–^15^N HMBC correlations (Fig. 2C) that were readily observed with our current NMR spectrometer (600 MHz, 3 mm cryogenic probe) but lacking in our original set of NMR data (500 MHz, 5 mm room temperature probe), provided strong evidence for placement of the nitrogen atoms within the structural framework of **1**.

We then applied the recently developed LR-HSQMBC^18^–^20^ and HSQMBC-TOCSY^21^ pulse sequences, which can extend the range of heteronuclear correlations to observe ^4^*J* and even some ^5^*J* and ^6^*J* correlations. These experiments complement the traditional HMBC experiment, which typically detects ^2^*J* and ^3^*J*, and only rarely ^4^*J* correlations. The additional long-range ^1^H–^13^C correlations detected in these experiments, including some ^2^*J*/^3^*J* couplings not seen in the HMBC data set, fully supported the proposed heterocyclic structure of **1** (Fig. 2D).

Another very useful experiment was 1,1-HD-ADEQUATE, which provides proton-detected visualization of one-bond ^13^C–^13^C homonuclear couplings.^22^–^24^,^30^ The correlations observed in a standard HMBC experiment are due to both 2-bond and 3-bond heteronuclear couplings, and the inability to distinguish between these alternatives can lead to ambiguous or biased interpretation of the data. The 1,1-HD-ADEQUATE experiment complements HMBC data by affording proton-detected ^1^*J*_CC_ correlations, which are functionally equivalent to ^2^*J*_CH_ HMBC correlations.^22^,^31^ These data can thus define direct carbon–carbon connectivities, which was particularly useful for establishing the location of quaternary carbons directly adjacent to protonated ones in compound **1** (Fig. 2E).

**Fig. 3:**
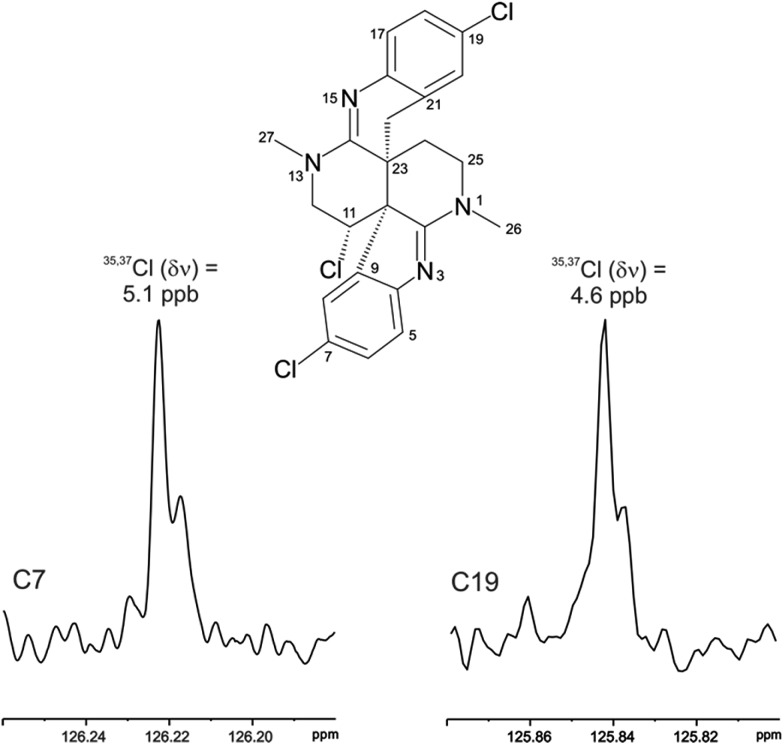
The ^35,37^Cl isotope effect observed in the bs-CLIP-HSQMBC experiment 1D ^13^C slices for C-7 and C-19 of caulamidine A (**1**). The isotope shifts of 5.1 and 4.6 ppb for C-7 and C-19 correspond to shifts of 0.77 and 0.69 Hz, respectively. While isotope effects of this magnitude have been observed in the past in 1D ^13^C NMR spectra, it is far easier to obtain the resolution to observe these effects using the bs-CLIP-HSQMBC experiment.

**Fig. 4:**
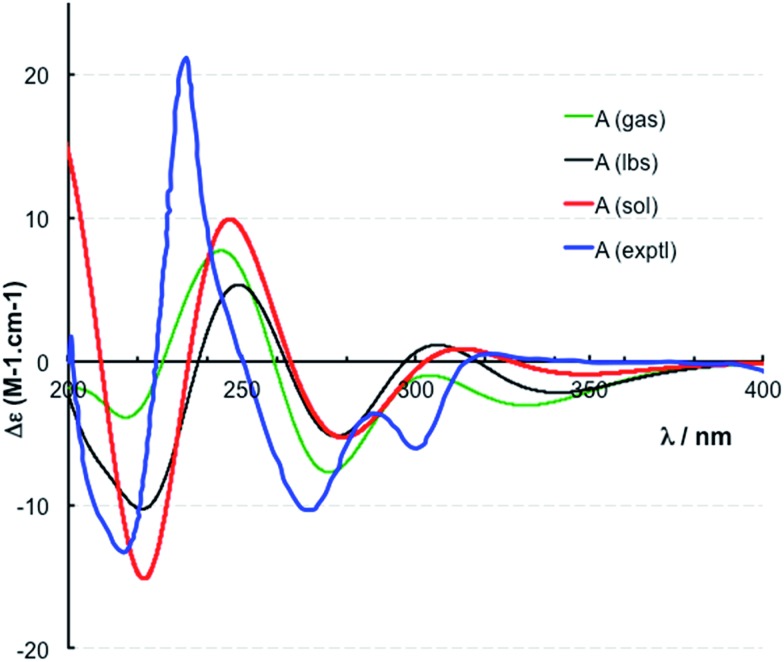
Experimental (exptl, blue) and computed ECD spectra of (10*S*,11*S*,23*S*)-caulamidine A at the B3LYP/6-31G** (gas, green) and B3LYP/6-311++G** (lbs, black) levels in the gas phase and at the B3LYP-SCRF(COSMO)/6-311++G**//B3LYP/6-311++G** (sol, red) level in MeOH.

The location of chlorine substituents in caulamidine A (**1**) was initially assigned solely from carbon/proton NMR chemical shift considerations. Application of a new band-selective CLIP-HSQMBC experiment, that can visualize the ^35,37^Cl isotope effect on both protonated and non-protonated ^13^C nuclei, provided unequivocal support for these assignments.^25^ Carbons substituted with ^37^Cl have a slightly different chemical shift compared to those substituted with ^35^Cl (δ*υ* ∼ 3–5 ppb). This chemical shift differential manifests in 2D correlation cross peaks that are split and 1D ^13^C slices that have a distinct shoulder (Fig. 3). The bs-CLIP-HSQMBC data for **1** clearly revealed the ^35,37^Cl isotope effect for C-7, C-11, and C-19, which definitively established chlorine-substitution at these positions. Once the 2D structure of **1** was firmly established, the relative configurations of the C-10, C-11, and C-23 stereogenic centers were defined by diagnostic NOE interactions (Fig. 2F). Key NOE enhancements included those between H-8/H-22a, H-11/H-24a, and H-22b/H-24b. These NOEs were confirmed from NMR experiments with the TFA salt of **1**, which provided greater dispersion of the proton signals (ESI†). The absolute configuration of caulamidine A (**1**) was then established by comparing its experimental ECD spectrum (Fig. 4) with the DFT-calculated simulations of exciton coupling between the aromatic chromophores (see ESI† for molecular modelling and computational details). This comparison permitted assignment of the absolute configuration of **1** as (10*S*, 11*S*, 23*S*).

**Fig. 5:**
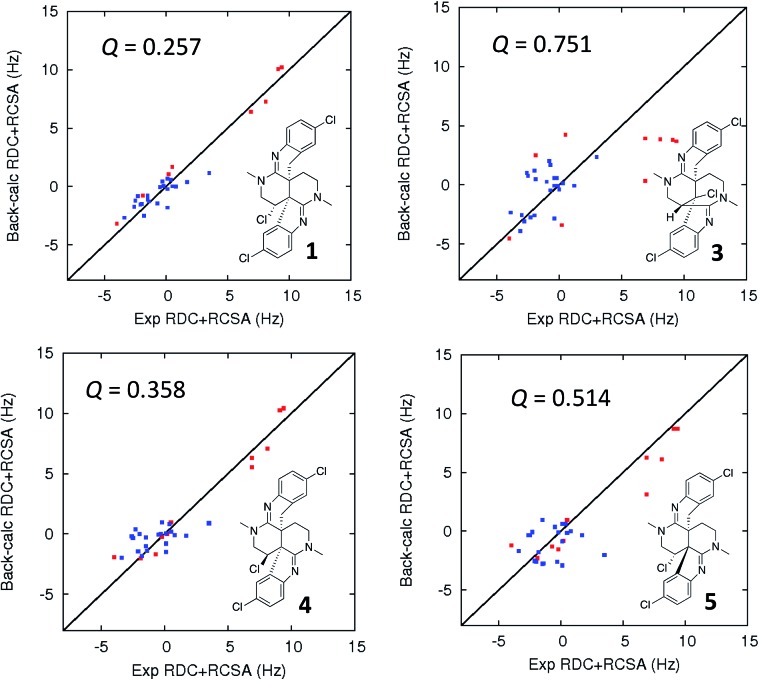
Comparison of the experimental *vs.* DFT-calculated RDC (red) and RCSA (blue) values for caulamidine A (**1**), the CASE-generated alternative structure **3**, and configurational isomers **4** and **5**. The *Q*-value is a quantitative similarity measurement for the DFT-calculated RDC and RCSA values for the structure compared to the experimentally measured data. RDC values define the orientation of the C–H bond vectors for protonated carbons, whereas RCSAs describe the chemical shift tensors for all carbons in the molecule's skeleton.^17^ For proton-deficient molecules, RCSA data can provide a better assessment of global structural correctness than sparsely available RDCs.

In addition to traditional interpretation and assignment of the spectroscopic data for caulamidine A, we also employed computer-assisted structure elucidation (CASE) analysis of the NMR and HRMS data for **1** using the ACD Laboratories Structure Elucidator CASE program^19^,^32^–^34^ to identify and rank potential alternative structures. This revealed only one other plausible structure, compound **3** (Fig. 5), based on the NMR connectivity data and predicted *vs.* calculated ^13^C chemical shift values. A suite of computational studies was then performed to compare the experimental and calculated values of chemical shifts, coupling constants, and free energy levels of the proposed and alternate structures **1** and **3**, respectively. By all these criteria, the assigned structure of caulamidine A (**1**) was confirmed. The critical value of the LR-HSQMBC and 1,1-HD-ADEQUATE experiments was underscored by results from the CASE analyses. When only data from conventional NMR methods including HSQC and HMBC were used, the CASE program ran for 250 hours without ever generating a single structure. When LR-HSQMBC and 1,1-HD-ADEQUATE data were added to the input file, the program ran for less than one second and structure **1** was the top candidate.

Ultimate verification of the structural proposal for caulamidine A was accomplished using both residual chemical shift anisotropy (RCSA) measurements^35^–^37^ and residual dipolar couplings (RDC).^38^–^40^ These NMR phenomena, which result from partial alignment of molecules in an anisotropic medium, carry rich structural information. The measured RDCs result from changes in heteronuclear (^1^H–^13^C) couplings and RCSAs arise from changes in the ^13^C chemical shielding tensor. Anisotropic NMR data can be employed to define the relative orientations of bonds and shielding or dipolar coupling tensors, regardless of the distance between them. They provide a powerful, independent means to assess the global correctness of a proposed structure and configuration, whether the structure is proposed by an investigator or derived from CASE program output.^17^,^28^ Applications of RDCs to biomolecules were first reported more than 20 years ago,^41^,^42^ while the first reported utilization of RDCs in small molecule structural analysis were those of Shapiro and co-workers.^43^,^44^ Subsequently, application of RDCs in small molecule structure elucidation/confirmation has been the subject of several chapters^45^–^47^ and a recent perspective paper.^40^ To date, there has not been a review of the applications of RDCs in natural product structural determination, but the recent chapter of Gil and Navarro-Vázquez does survey applications of RDCs for natural product structure confirmation.^47^

The fundamentals of residual chemical shift anisotropy (RCSA) have been recognized for many years, but the ability to measure these effects in small molecules has been hampered by the difficulty of separating the isotropic from the anisotropic component of the chemical shift. Only recently has a practical approach been described for obtaining reliable RCSA measurements using a polymeric gel (ESI†) and specially constructed NMR tubes.^35^ It is in this context that RCSA and RDC values were experimentally determined for caulamidine A and then compared to the DFT-calculated values for structure **1** and the alternative structure **3** (Fig. 5) generated using the Structure Elucidator CASE program. The *Q*-factor, which is a quantitative assessment of the quality of the fit between experimental and calculated values, was almost three times worse for **3** than for **1**. The difference between calculated and experimental ^13^C chemical shifts was also significantly higher for structure **3** [*d*_N_(^13^C) = 4.323] compared to **1** [*d*_N_(^13^C) = 2.685]. These analyses, where the correct structure should have the lowest *Q* and *d*_N_(^13^C) values, ruled out structure **3** and conclusively affirmed the structure assigned for caulamidine A (**1**). We also examined the fit of the experimental RDC/RCSA data to the two other energetically feasible stereoisomers of **1**. Here again, the best agreement was obtained to **1** itself, with the two other viable isomers, **4** and **5**, giving substantially higher *Q*-values (Fig. 5).

The structural assignment of caulamidine A (**1**) was thus facilitated by an array of new heteronuclear NMR experiments, along with CASE analysis and advanced DFT computational techniques. The structure was then validated by analysis of the RDC and RCSA anisotropy parameters, which provide an orthogonal and unequivocal means of confirming the overall correctness of a molecular structure.^17^ The only other application of both RDCs and RCSAs in the structural elucidation of a new natural product was the recent study of homodimericin A.^37^

Caulamidine B (**2**) was isolated as a glassy solid and its molecular formula, established by HRESIMS as C_23_H_21_ClBr_2_N_4_, was similar to **1** except for halogen content. By using the same conventional NMR experiments and the more recent heteronuclear experiments that were employed with caulamidine A, the carbon and nitrogen molecular framework of **2** was revealed to be the same as **1** (ESI†). However, examination of ^1^H–^1^H couplings and some key HMBC correlations indicated that halogen substitutions in the aromatic C- and F-rings of caulamidine B were different from caulamidine A. The H-5 singlet (*δ* 7.37) showed a 3-bond HMBC correlation to N-3 while the *ortho*-coupled H-8 doublet (*δ* 6.89, 8.4 Hz) correlated with the bridgehead C-10. This supported halogen substitution at C-6 in **2** instead of C-7 as in **1**. In a similar manner, HMBC correlations from the H-17 singlet (*δ* 7.13) to N-15 and from the H-20 doublet (*δ* 6.86, 8.4 Hz) to C-22 required halogen substitution at C-18 in **2**. The regiochemistry of chlorine- *vs.* bromine-substitution in caulamidine B (**2**) was defined using a high-resolution, band-selective HSQC experiment recently described by the Molinski laboratory to assign halogen substitution patterns in a series of polyhalogenated natural products.^26^,^27^ This is a sensitive technique for detecting the ^35,37^Cl isotope effect, but it is only applicable for chlorinated carbons that are also protonated. The bs-HSQC experiment provides characteristic split 2D cross peaks and shoulders on the 1D ^13^C slices for Cl-substituted carbons, similar to the bs-CLIP-HSQMBC experiment employed with **1**. Using this technique, the ^35,37^Cl isotope effect was clearly observed for C-11 (ESI†), which then required Br-substitution at C-6 and C-18 in **2**. The relative configuration was assigned from diagnostic NOE interactions measured in **2** that were similar to those observed in **1**, while the absolute configuration was established as (10*S*, 11*S*, and 23*S*) by comparing the experimental and DFT-calculated ECD spectra (ESI†).

We recently found that the eudistidines, a different class of heterocyclic marine alkaloids,^48^ exhibited antimalarial activity, thus, we also evaluated the caulamidines for antimalarial effects against chloroquine-sensitive (D6) and chloroquine-resistant strains of the *Plasmodium falciparum* parasite.^49^ Caulamidines A (**1**) and B (**2**) showed similar inhibitory effects against both strains of *P. falciparum* with IC_50_ values that ranged from 8.3–12.9 μM (ESI†). Caulamidine A (**1**), was also tested for cytotoxic activity in the single dose (40 μM) NCI-60 cell screen. At this concentration it showed only modest growth inhibition against a very small subset of human cell lines, revealing a significant concentration differential between its antimalarial activity and cytotoxic effects.

## Conclusions

Caulamidines A (**1**) and B (**2**) share a novel heterocyclic scaffold with no close precedents in the chemical literature. A likely precursor is tryptamine or tryptophan, but it is not apparent how the unique heterocyclic core of the caulamidines is biosynthesized, so biosynthesis arguments to help define and rationalize the structures were not viable. However, unambiguous assignment of the caulamidine structures was possible using a suite of powerful new NMR techniques, complemented with CASE analysis and DFT-computational studies. New NMR pulse sequences were utilized to extend long-range heteronuclear connectivities *via*^4^*J* and even ^5^*J* couplings, while the 1,1-HD-ADEQUATE experiment revealed ^1^*J*_CC_ couplings that are functionally equivalent to 2-bond HMBC correlations.^31^ The additional heteronuclear correlation data provided by these experiments supplemented more traditional NMR methods and permitted verification of the structural framework of the caulamidines. The value of these experiments was evident from the dramatic reduction in time for successful CASE analysis that resulted when they were included in the caulamidine NMR data sets being analyzed. High-resolution 2D NMR experiments, which allow visualization of the ^35,37^Cl isotope effect, clearly defined sites of chlorination for both protonated (bs-HSQC) and quaternary (bs-CLIP-HSQMBC) carbons. The assigned structure and configuration of caulamidine A (**1**) was then confirmed by anisotropic NMR experiments to assess the 3D relative orientation of C–H bonds (RDC) and carbon chemical shielding tensors (RCSA), which are not dependent on the distances between bonds and atoms. These data provided a complementary means to assess the global correctness of a molecular structure and thus verify or refute the constitution and configuration of an assigned structure.^17^ Prior application of these new NMR methodologies have largely focused on proof-of-principle studies using known compounds or the application of a single technique to resolve unanswered structural questions. While a number of these NMR techniques were used in the structural assignment of homodimericin A,^37^ the breadth of NMR experiments employed to unequivocally define the caulamidine structures is heretofore unprecedented.

As illustrated by our caulamidine studies, concerted application of contemporary NMR and computational techniques can provide valuable data to help correctly define the complex organic structures often found in natural products. They provide additional means to deduce and evaluate 2D structural assignments as well as to confirm stereochemical features. In concerted applications, these recent advancements provide powerful new tools that can help resolve challenging structural problems, while reducing misassignments and the resulting propagation of incorrect structures. The continuing development and application of new NMR methods that expand the boundaries for data acquisition and structural characterization will further advance natural products discovery, development, and total synthesis efforts.

## Author contributions

Dennis Milanowski; compound isolation, NMR spectroscopic characterization, structural elucidation. Naoya Oku; experimental ECD studies. Laura Cartner; compound isolation, NMR spectroscopic characterization. Heidi Bokesch; structural characterization, HR mass spectrometry. R. Thomas Williamson; NMR experimentation and data analysis, manuscript preparation. Josep Saurí; NMR experimentation and data analysis. Yizhou Liu; NMR experimentation and data analysis. Kirill A. Blinov; computer-assisted structural elucidation studies. Yuanqing Ding; theoretical ECD and other DFT computation studies. Xing-Cong Li; theoretical ECD and other DFT computation studies. Daneel Ferreira; theoretical ECD and other DFT computation studies. Larry A. Walker; theoretical ECD and other DFT computation studies. Shabana Khan; antimalarial testing. Michael Davies-Coleman; molecular modeling and computational studies. James Kelley; HR mass spectrometry analyses. James McMahon; chemical and biological data analysis, project selection and coordination, editorial assistance. Gary Martin; coordination of NMR studies, NMR data collection and analysis, manuscript preparation. Kirk Gustafson; structural elucidation and verification, NMR data analysis, project coordination, overview and management, manuscript preparation.

## Conflicts of interest

There is no conflict to declare.

## Supplementary Material

Supplementary information
