# Supplementary material for: Unequivocal determination of caulamidines A and B: application and validation of new tools in the structure elucidation tool box
Source: Chem Sci. 2017 Nov 6;9(2):307–14. doi: 10.1039/c7sc01996c (PMC5868047; doi:10.1039/c7sc01996c)

Electronic Supplementary Material (ESI) for Chemical Science.
This journal is © The Royal Society of Chemistry 2017

Unequivocal determination of caulamidines A and B: application and validation of new tools in the structure elucidation tool box

Dennis J. Milanowski,[a,‡] Naoya Oku,[a,§] Laura K. Cartner,[a,b] Heidi R. Bokesch,[a,b] R. Thomas Williamson,[c] Josep Saurí,[c] Yizhou Liu,[c] Kirill A. Blinov,[d] Yuanqing Ding,[e] Xing-Cong Li,[e] Daneel Ferreira,[e] Larry A. Walker,[e] Shabana Khan,[e] Michael T. Davies-Coleman,[f,∇] James A. Kelley,[g] James B. McMahon,[a] Gary E. Martin,[*,c] Kirk R. Gustafson[*,a]

[a]Molecular Targets Laboratory, Center for Cancer Research, National Cancer Institute, Frederick, Maryland 21702-1201, United States

[b]Basic Science Program, Leidos Biomedical Research, Inc., Frederick National Laboratory for Cancer Research, Frederick, Maryland 21702-1201, United States

[c]NMR Structure Elucidation, Process Research and Development, Merck & Co. Inc., Rahway, New Jersey 07065, United States

[d]Molecule Apps, LLC, Corvallis, Oregon 97330, United States

[e]National Center for Natural Products Research and Department of BioMolecular Sciences, School of Pharmacy, University of Mississippi, Oxford, Mississippi 38655, United States

[f]Department of Chemistry, Rhodes University, Grahamstown, South Africa

[g]Chemical Biology Laboratory, Center for Cancer Research, National Cancer Institute, Frederick, Maryland 21702-1201, United States

Supplemental Information:

S3-5	General experimental, animal material, isolation
S6	Mass guided LC-MS purification of caulamidines A (**1**) and B (**2**)
S7	Table SI 1. NMR data for caulamidine A (**1**)
S8	LR-HSQMBC & HSQMBC-TOCSY correlations for caulamidine A (**1**)
S9	Table SI 2. NMR data for caulamidine B (**2**)
S10	LR-HSQMBC, HSQMBC-TOCSY, and NOE correlations for caulamidine B (**2**)
S11-12	ECD and computational analysis of caulamidines A (**1**) and B (**2**)
S13	Figure SI 1. Optimized geometries of (10*S*,11*S*,23*S*)-caulamidines A **(1)** and B **(2)**
S14	Figure SI 2. Experimental and DFT calculated ECD spectra of caulamidine A (**1**)
S15	Figure SI 3: Key molecular orbitals in the calculated ECD of caulamidine A (**1**)
S16	Figure SI 4. Experimental and DFT calculated ECD spectra of caulamidine B (**2**)
S17	Table SI 3. Calculated ECD parameters for caulamidine A (**1**)
S18	Table SI 4. Calculated interatomic distances for caulamidines A (**1**) and B (**2**)
S19	Table SI 5. Comparison of DFT-calculated and experimental ^{13}C NMR chemical shift values for caulamidine A (**1**) and caulamidine B (**2**)
S20	Table SI 6. Experimental RDC data of caulamidine A (**1**)
S21	Table SI 7. Experimental RCSA data of caulamidine A (**1**)
S22	^{35}Cl/^{37}Cl isotope effect observed in bc-HSQC for C-11 of caulamidine B (**2**)
S23	Antimalarial screening assay
S24	References
S25	Caulamidine A (**1**) ^{1}H NMR spectrum (CD_3CN)
S26	Caulamidine A (**1**) ^{13}C NMR spectrum (CD_3CN)
S27	Caulamidine A (**1**) COSY spectrum (CD_3CN)
S28	Caulamidine A (**1**) HSQC spectrum (CD_3CN)
S29	Caulamidine A (**1**) ^{1}H-^{13}C HMBC spectrum (CD_3CN)
S30	Caulamidine A (**1**) NOESY spectrum (CD_3CN)

S31 Caulamidine A (**1**) ROESY spectrum (CD_3CN)
S32 Caulamidine A (**1**) ^1H-^{15}N HMBC spectrum (CD_3CN)
S33 Caulamidine A (**1**) ^1H-^{13}C LR-HSQMBC spectrum (CD_3CN) optimized for 2Hz
S34 Caulamidine A (**1**) ^1H-^{13}C HSQMBC-TOCSY spectrum (CD_3CN) optimized for 8Hz + 60 ms mixing time
S35 Caulamidine A (**1**) ^1H-^{13}C HSQMBC-TOCSY spectrum (CD_3CN) optimized for 4Hz + 40 ms mixing time
S36 Caulamidine B (**2**) ^1H NMR spectrum (CD_3CN)
S37 Caulamidine B (**2**) ^{13}C NMR spectrum (CD_3CN)
S38 Caulamidine B (**2**) COSY spectrum (CD_3CN)
S39 Caulamidine B (**2**) HSQC spectrum (CD_3CN)
S40 Caulamidine B (**2**) ^1H-^{13}C HMBC spectrum (CD_3CN)
S41 Caulamidine B (**2**) NOESY spectrum (CD_3CN)
S42 Caulamidine B (**2**) ROESY spectrum (CD_3CN)
S43 Caulamidine B (**2**) ^1H-^{15}N HMBC spectrum (CD_3CN)
S44 Caulamidine B (**2**) ^1H-^{13}C LR-HSQMBC Spectrum (CD_3CN) optimized for 2Hz
S45 Caulamidine B (**2**) ^1H-^{13}C LR-HSQMBC Spectrum (CD_3CN) optimized for 8Hz + 60 ms mixing time
S46 Caulamidine A (**1**) HD-J-HSQC spectra for RDC measurement
S47 Caulamidine A (**1**) $\{^1H\}$-^{13}C spectra for RCSA measurement
S48-49 Coordinates of caulamidine A (**1**) from DFT geometry optimization
S50-56 GIAO chemical shielding tensors of caulamidine A (**1**)
S57 Table SI 8. NMR Data for Caulamidine A (**1**) TFA Salt in CD_3CN
S58 Caulamidine A (**1**) TFA salt ^1H NMR spectrum (CD_3CN)
S59 Caulamidine A (**1**) TFA salt ^{13}C NMR spectrum (CD_3CN)
S60 Caulamidine A (**1** TFA salt) COSY spectrum (CD_3CN)
S61 Caulamidine A (**1**) TFA salt HSQC spectrum (CD_3CN)
S62 Caulamidine A (**1**) TFA salt ^1H-^{13}C HMBC spectrum (CD_3CN)
S63 Caulamidine A (**1**) TFA salt ROESY spectrum (CD_3CN)
S64 Caulamidine A (**1**) TFA salt 1D NOE spectrum-1 (CD_3CN)
S65 Caulamidine A (**1**) TFA salt 1D NOE spectrum-2 (CD_3CN)
S66 Caulamidine A (**1**) TFA salt 1D NOE spectrum-3 (CD_3CN)
S67 Caulamidine A (**1**) TFA salt 1D NOE spectrum-4 (CD_3CN)

General Experimental Methods. CD spectra were obtained in CH_3CN or MeOH with a Jasco J-720 spectropolarimeter using a microvolume disk cell (0.1 mm thickness). The free base of caulamidines A (**1**) and B (**2**) were prepared by washing with 1% aqueous triethylamine followed by H_2O. NMR spectra were acquired in CD_3CN using a Bruker AVANCE III NMR spectrometer equipped with either a 3 mm TCI or 1.7 mm TXI cryogenic probe, and operating at 600 MHz for ^1H and 150 MHz for ^{13}C. NMR spectra were also acquired with a Varian Inova spectrometer equipped with a 5 mm room temperature probe and operating at 500 MHz for ^1H and 125 MHz for ^{13}C. Spectra were referenced to the residual nondeuterated solvent signals at δ_H 1.93 and at δ_C 1.30. ^1H-^{13}C HMBC data sets were acquired using J_{CH} values of 3.5 Hz, 8.3 Hz, and 11 Hz. ^1H-^{15}N HMBC data sets were acquired using a J_{NH} value of 8.0 Hz. LR-HSQMBC were optimized for 2 Hz coupling and 1,1-HD-ADEQUATE for 40 Hz. Anisotropic NMR data were acquired for 1mg of caulamidine A in a pHEMA (poly-(2-hydroxyethyl methacrylate)) gel cross-linked with EGDMA (ethylene glycol dimethylacrylate) with a HEMA monomer concentration of 60% (v/v) and a cross-linking ratio of 0.07% (v/v).[1] Weak and strong alignment conditions were achieved with an NMR stretching tube with inner diameters of 4.2mm and 3.2mm for the wide and thin sections, respectively.[2] RDCs were measured with the HD-*J*-HSQC (homonuclear decoupled *J*-resolved HSQC) experiment,[3] with a recycling delay of 1.5s, an F_1 acquisition time of 256ms on a spectral window of 600Hz, an F_2 acquisition time of 120ms, and a transient number of 96 for both weak and strong alignment conditions. Carbon RCSA were measured with the $\{^1H\}$-^{13}C experiment with a recycling delay of 1.5s, an acquisition time of 0.55s, and transient numbers of 25600 and 76800 for weak and strong alignment conditions, respectively. All anisotropic NMR measurements were conducted at 25°C on a Bruker 500MHz spectrometer equipped with a Prodigy™ probe. (+)HRESIMS data were acquired on an Agilent Technology 6530 Accurate-mass Q-TOF LC/MS. Positive-ion, fast-atom bombardment mass spectra (HR-FABMS) were obtained on a double-focusing mass spectrometer using a sample matrix of nitrobenzyl alcohol. Preparative reversed-phase HPLC was run on an Agilent 1260 Infinity HPLC using a Phenomenex Luna-C_{18}(2) (5μ, 100Å,150 x10 mm) column with 0.1% formic acid or a Dynamax C_{18} (60 Å, 1 x 25 cm) column with 0.1% TFA.

Animal Material. Samples of the marine bryozoan *Caulibugula intermis* were collected and identified by P. L. Colin (Coral Reef Research Foundation) in the South Pacific near Palau. Animal material was frozen shortly after collection and maintained frozen prior to extraction. Voucher specimens for the original collection (0CDN1079, C011545) and later recollections (0YYA1176-T, C034489 and 0YYA0799-J, C034487) are maintained at the Smithsonian Institution, Washington, D.C.

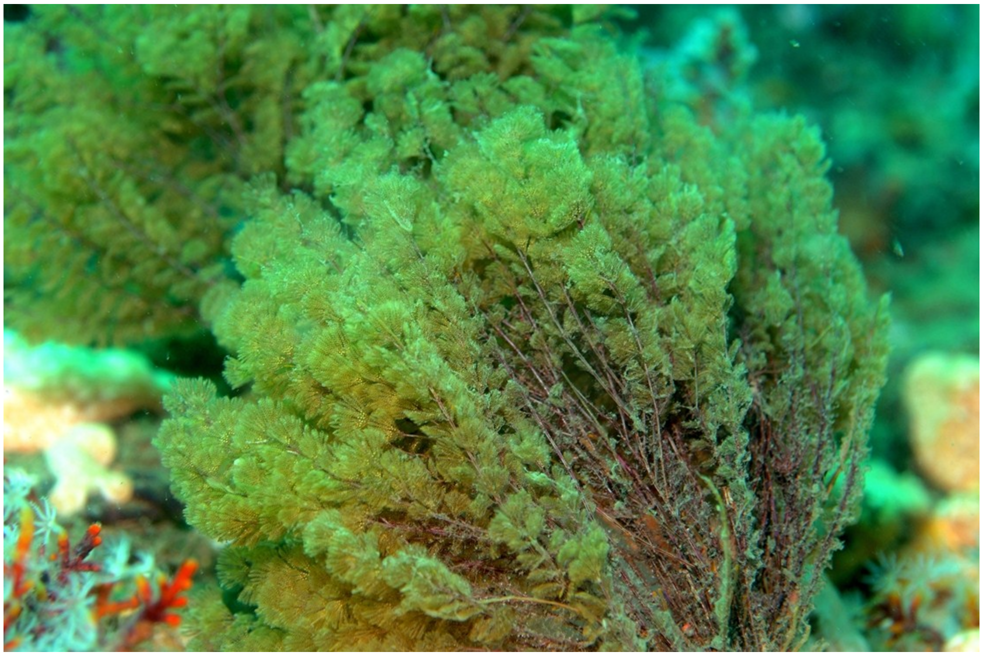

In situ photograph of *Calibugula intermis*

Isolation. The frozen bryozoan from the original collection (227.7 g) was ground and extracted with H_2O to yield 25.9 g of aqueous extract after lyophilization. The animal material was then extracted with CH_2Cl_2-MeOH (1:1) followed by MeOH (100%) to give 5.14 g of combined organic extract after removal of the solvent. The crude organic extract was fractionated by solvent-solvent partitioning as described previously.[4] The methyl *tert*-butyl ether (MTBE) soluble material (1.19 g) was repeatedly chromatographed on Sephadex LH-20 (2 × 125 cm) eluting with hexane-CH_2Cl_2-MeOH (2:5:1), monitoring at 254 nm. Final purification was achieved by C_{18} HPLC (Dynamax 60 Å, 1 x 25 cm) eluted with a linear H_2O/CH_3CN gradient (0.1% TFA vol/vol) from 0 to 100% CH_3CN over 30 min to give a total of 3.7 mg of caulamidine A (**1**). The *Caulibugula intermis* recollections (981 g) were extracted in a similar manner to provide a total of 8.7 g of organic solvent extract. Solvent partitioning and mass-guided HPLC purification using a Phenomenex Luna-C_{18}(2) (5μ, 100Å,150 x10 mm) column and a linear gradient from 95% H_2O/5% CH_3CN to 100% CH_3CN over 20 minutes (all solvents contained 0.1% formic acid) provided 14.8 mg caulamidine A (**1**) and 4.7 mg caulamidine B (**2**).

Caulamidine A (1): glassy solid; $[\alpha]_D$ -5.6 (*c* 0.1, CH_3CN); UV (CH_3CN) λ_{max} 320 (sh, ε 4,100) 282 (ε 15,700), 220 (ε 19,500) nm; CD (CH_3CN, 8.19 × 10^{-4} M) λ_{ext} (Δε) 314 (1.37), 302 (0.0), 265 (-5.22), 237 (0.0), 229 (1.84), 223 (0.0), 206 (-4.17) nm; ^{1}H NMR and ^{13}C data, see Table SI 1; HRFABMS $[M + H]^+$ *m/z* 459.0924, calcd for $C_{23}H_{22}{}^{35}Cl_3N_4$, 459.0910 (Δ 1.4 mDa).

Caulamidine B (2): glassy solid; $[\alpha]_D$ -2.7 (*c* 0.1, CH_3OH); UV (CH_3OH) λ_{max} 293 (ε 6,740) 234 (ε 11,850) nm; CD (CH_3OH, 1.82 × 10^{-4} M) λ_{ext} ($\Delta\varepsilon$) 296 (0.36), 293 (0.0), 267 (-6.45), 247 (0.0), 239 (13.64), 217 (0.0), 209 (-2.64) nm; ^1H NMR and ^{13}C data, see Table SI 2; HRESIMS $[M + H]^+$ *m/z* 546.9891, calcd for $C_{23}H_{22}{}^{35}Cl^{79}Br_2N_4$, 546.9900 ($\Delta$ -0.9 mDa).

Mass guided LC-MS purification of caulamidines A (*m/z* 458.6-459.6) and B (*m/z* 550.6-551.6).

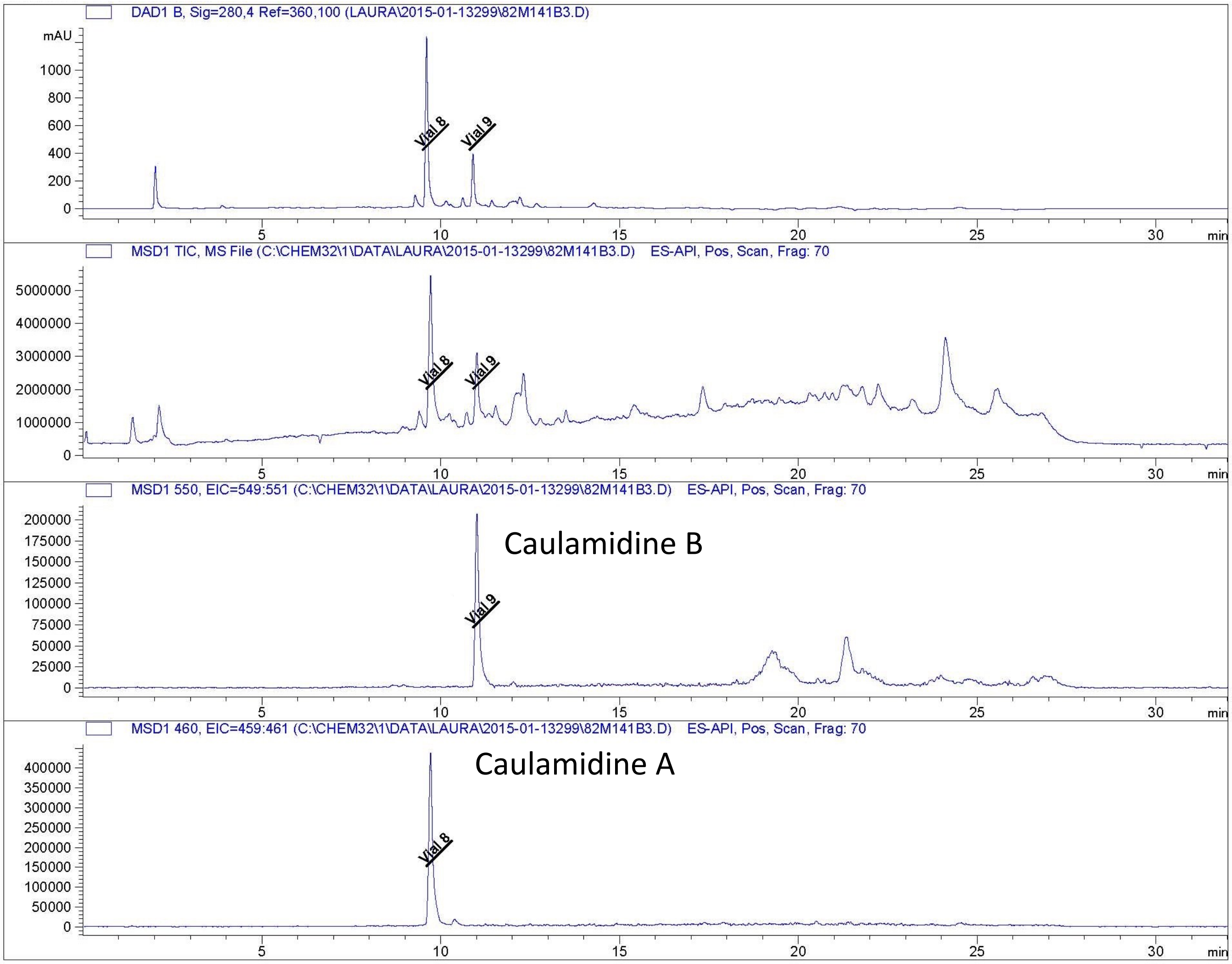

Table SI 1. NMR data for caulamidine A (1) in CD_3CN.

Position'	! '(^{13}C/^{15}N)'	^{1}∀ ! '(mult,'Hz)'	HMBC'	LR:HSQMBC*'	'HSQMBC:TOCSY**'	1,1:HD:ADEQUATE'
1:N'	78.9'	''	''	''	''	''
2'	174.0'	''	''	''	''	''
3:N'	241.7'	''	''	''	''	''
4'	156.0'	''	''	''	''	''
5'	117.8'	7.17'(d,'8.5)'	N3,'C4,'C6,'C7,'C9'	C8'(4*J*),'C10'(4*J*)'	''	C4,'C6'
6'	129.4'	7.31'(dd,'8.4,'2.0)'	C4,'C5,'C7,'C8'	C9'(4*J*),'C10'(5*J*)'	''	C5,'C7'
7'	126.3'	''	''	''	''	''
8'	123.8'	6.95'(bs)'	C4,'C6,'C7,'C10'	C5'(4*J*),'C9'(2*J*)'	''	C7,'C9'
9'	133.3'	''	''	''	''	''
10'	58.9'	''	''	''	''	''
11'	54.8'	5.02'(dd,'10.8,'4.7)'	C2,'C9,'C10,'C12,'C23'	C14'(4*J*)'	N13'(3*J*),'N15'(5*J*)'	C10,'C12'
12a'	52.6'	3.87'(dd,'13.3,'6.6)'	N13,'N15,'C11,'C14,'C27'	''	C9'(4*J*)'	C11'
''''b'	''	3.66'(dd,'13.3,'10.5)'	N13,'N15,'C11,'C14'	C16'(5*J*),'C27'(3*J*)'	C9'(4*J*)'	C11'
13:N'	87.5'	''	''	''	''	''
14'	159.1'	''	''	''	''	''
15:N'	216.6'	''	''	''	''	''
16'	143.9'	''	''	''	''	''
17'	124.2'	6.94'(d,'8.2)'	N15,'C16,'C19,'C21'	C20'(4*J*)'	''	C16,'C18'
18'	127.2'	7.12'(dd,'8.2,'2.4)'	C16,'C19'	C21'(4*J*)'	N15'(4*J*),'C22'(5*J*)'	C17'
19'	125.8'	''	''	''	''	''
20'	127.3'	6.96'(bs)'	C16,'C18,'C19,'C21,'C22'	C17'(4*J*)'	C23'(4*J*)'	C19,'C21'
21'	125.4'	''	''	''	''	''
22a'	29.6'	2.48'(d,'15.9)'	C10,'C14,'C16,'C21,'C23,'C24'	''	''	''
''''b'	''	2.28'(d,'15.9)'	N13,'C10,'C14,'C16,'C21,'C23,'C24'	''	''	C21,'C23'
23'	39.8'	''	''	''	''	''
24a'	24.7'	2.25'(m)'	C10,'C14,'C22,'C23,'C25'	''	''	C23'
''''b'	''	1.73'(dd,'15.0,'6.2)'	N1,'C10,'C22,'C23,'C25'	C2'(4*J*),'C9'(4*J*)'	''	C23,'C25'
25a'	47.4'	3.38'(ddd,'12.5,'7.5,'1.6)'	N3,'C2,'C24'	C23'(3*J*)'	C10'(4*J*),'C22'(4*J*)'	C24'
''''b'	''	3.18'(dt,'11.7,'5.9)'	C24,'C26'	C23'(3*J*)'	C10'(4*J*),'C22'(4*J*)'	C24'
26'	37.2'	3.00'3H'(s)'	N1,'N3,'C2,'C25'	C23'(5*J*)'	''	''
27'	35.8'	3.24'3H'(s)'	N13,'N15,'C12,'C14'	C16'(5*J*),'C23'(4*J*)'	''	''

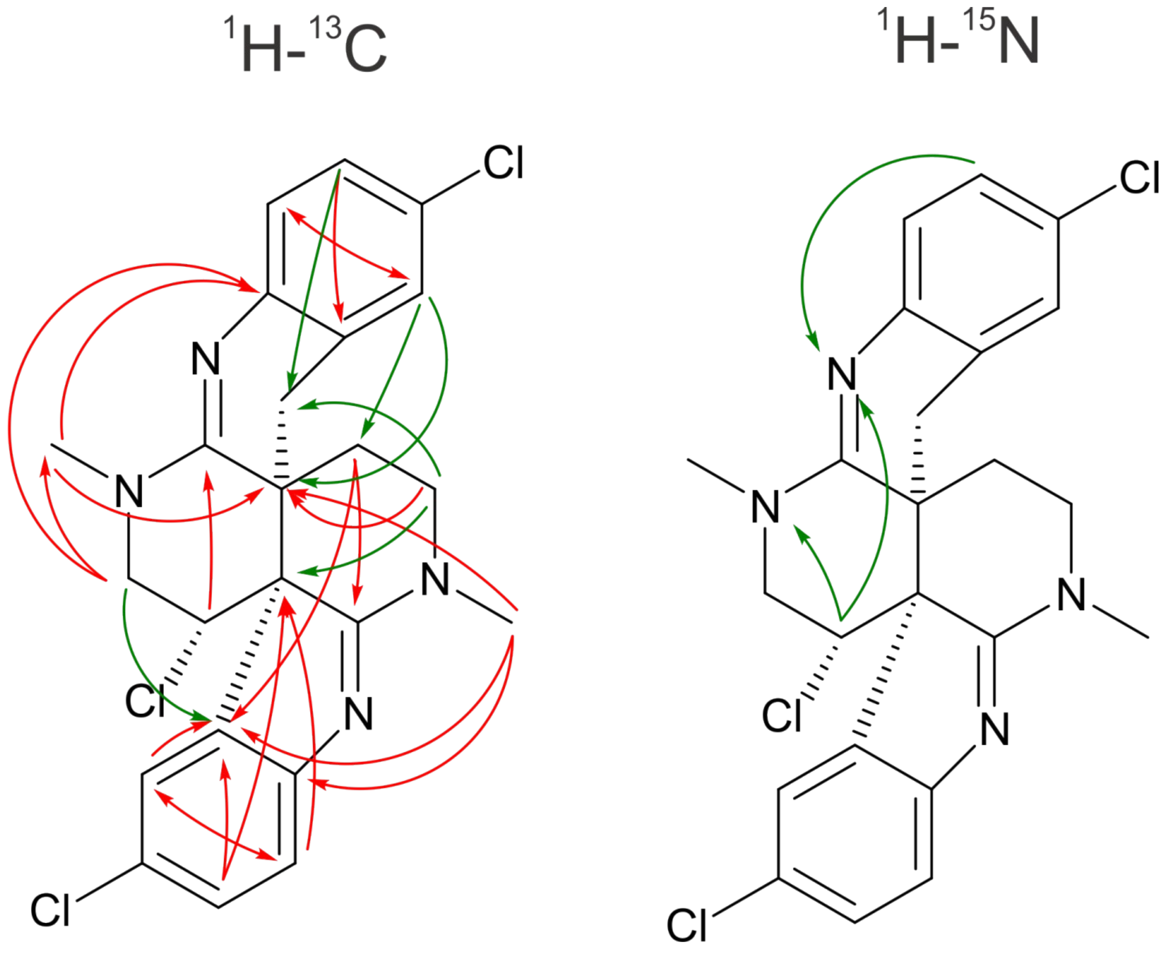

Additional correlations for caulamidine A (**1**) obtained from LR-HSQMBC with respect to HMBC are highlighted in red. Additional correlations obtained from HSQMBC-TOCSY with respect to both HMBC and LR-HSQMBC are highlighted in green.

Table SI 2. NMR data for caulamidine B (2) in CD_3CN.

Position'	! '(^{13}C/^{15}N)'	1H'(mult,'Hz)'	HMBC'	LR:HSQMBC'	HSQMBC:TOCSY'
1:N'	80.3'	''	''	''	''
2'	174.4'	''	''	''	''
3:N'	240.3'	''	''	''	''
4'	159.1'	''	''	''	''
5'	119.7'	7.37'(s)'	N3,'C4,'C6,'C9'	C8'(4*J*),'C10'(4*J*)'	''
6'	122.5'	''	''	''	''
7'	123.8'	7.12'(d,'10.6)'	C5,'C6,'C9'	C4'(4*J*),'C8'(2*J*),'C10'(4*J*)'	''
8'	125.1'	6.89'(d,'8.4)'	C4,'C6,'C10'	C5'(4*J*),'C11'(4*J*),'C23'(4*J*)'	''
9'	130.4'	''	''	''	''
10'	58.4'	''	''	''	''
11'	54.7'	5.01'(dd,'11.1,'6.5)'	C2,'C9,'C10,'C12,'C23'	''	''
12'	52.7'	3.87'(dd,'13.5,'6.5)'	N13,'N15,'C10,'C11,'C14,'C27'	''	C9'(4*J*)'
''	''	3.66'(t,'11.3)'	N13,'N15,'C11,'C14'	C10'(3*J*)'	C9'(4*J*)'
13:N'	89.0'	''	''	''	''
14'	159.7'	''	''	''	''
15:N'	216.3'	''	''	''	''
16'	146.9'	''	''	''	''
17'	125.2'	7.13'(bs)'	N15,'C16,'C19,'C21'	C20'(4*J*)'	''
18'	120.2'	''	''	''	''
19'	124.3'	6.99'(dd,'8.0,'2.1)'	C17,'C18,'C20,'C21'	''	C16'(4*J*),'C22'(4*J*)'
20'	129.2'	6.86'(d,'8.4)'	C16,'C18,'C22'	''	''
21'	122.5'	''	''	''	''
22'	29.3'	2.38'(d,'15.9)'	C10,'C14,'C16,'C20,'C21,'C23,'C24'	''	''
''	''	2.28'(d,'15.9)'	N13,'C10,'C14,'C16,'C20,'C21,'C23,'C24'	''	''
23'	39.6'	''	''	''	''
24'	24.6'	2.23'(dd,'15.1,'5.9)'	C22,'C23,'C25'	''	''
''	''	1.74'(dd,'15.1,'5.9)'	N1,'C10,'C22,'C23,'C25'	''	''
25'	47.4'	3.41'(dd,'13.0,'6.8)'	N3,'C2,'C23,'C24'	C26'(3*J*)'	''
''	''	3.18'(dt,'12.1,'6.0)'	N1,'C24,'C26'	''	''
26'	37.2'	3.01'3H'(s)'	N1,'N3,'C2,'C25'	''	''
27'	35.7'	3.23'3H'(s)'	N13,'N15,'C12,'C14'	''	''

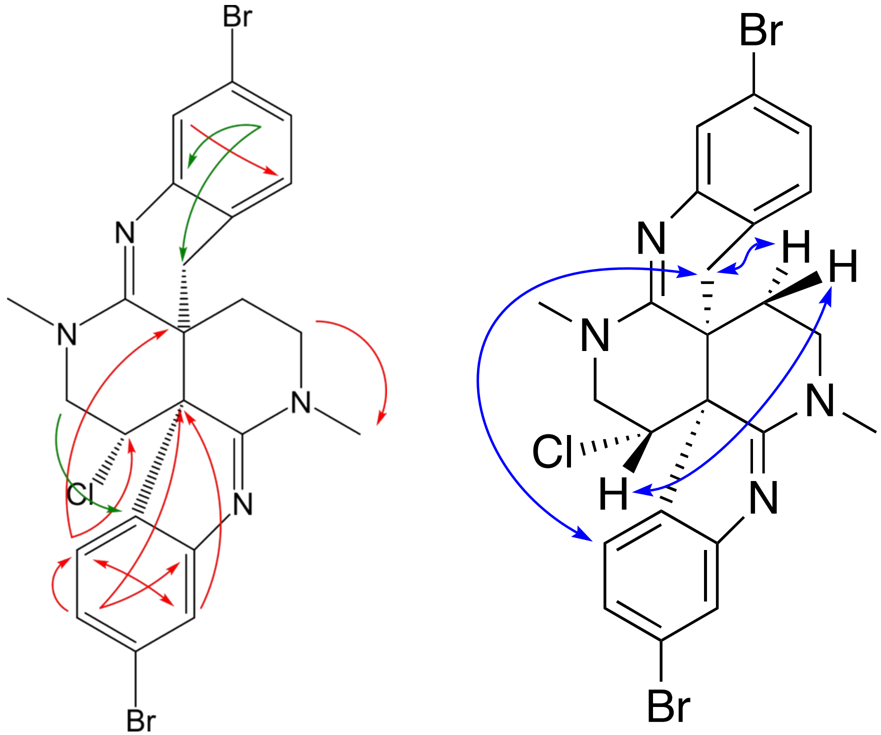

Additional ^{1}H-^{13}C correlations for caulamidine B (**2**) obtained from LR-HSQMBC with respect to HMBC are highlighted in red. Additional correlations obtained from HSQMBC-TOCSY with respect to both HMBC and LR-HSQMBC are highlighted in green. NOESY and ROESY correlations are in blue.

ECD and computational analysis of caulamidines A (1) and B (2)

Based on extensive NMR analysis, the absolute configurations (AC) of caulamidines A (**1**) and B (**2**) were determined to be either 10*S*, 11*S*, 23*S* or the 10*R*, 11*R*, 23*R* enantiomer. Electronic circular dichroism (ECD) data were computed to facilitate the AC assignments.[5-8] The 10*S*, 11*S*, 23*S* configuration was employed for the conformational random search with an energy window of 130 kJ/mol by using the OPLS_2005 force field in MacroModel,[9] yielding seven conformers with only one within an energy cut-off of 19 kJ/mol. This lowest energy conformer was used for the geometry optimization followed by harmonic vibrational frequency computation at the B3LYP/6-31G** and B3LYP/6-311++G** levels in the gas phase (Figure SI 1), and subsequently by calculation of excitation energies and rotatory strengths at the B3LYP/6-31G** and B3LYP/6-311++G** levels in the gas phase, and at the B3LYP-SCRF(COSMO)/6-311++G**//B3LYP/6-311++G** level in MeOH (Figure SI 2). All computations at the quantum mechanics levels were performed using the Gaussian 09 software packages.[10] The simulated ECD spectra at the above levels overall match the experimental ECD curve.

Molecular orbital analysis was carried out at the B3LYP-SCRF(COSMO)/6-311++G**//B3LYP/6-311++G** level in MeOH (Figure SI 3). Interestingly, orbitals O115 and O118 involve a $^{13}_{10}\pi$ bonding, and orbitals O120 and O122 a $^{13}_{10}\pi^*$ bonding, both delocalizing 13 electrons at 10 atoms including N-1 – C-9 and Cl at C-7. Similarly, orbitals O115 and O117 also involve a $^{13}_{10}\pi$ bonding, and orbitals O121 and O123 a $^{13}_{10}\pi^*$ bonding, both involving 13 electrons at 10 atoms including N-13 – C-21 and Cl at C-19. The experimentally observed low amplitude positive Cotton effect (CE) at 323 nm is attributed to the electronic transition (ET) at 309 nm from orbital O118 to its unoccupied LUMO orbital O120 (Table SI 3, Figure SI 3). The broad negative CEs in the 313 - 250 nm region are generated by the ETs at 316, 283, 281, and 279 nm. The negative CE at 323 nm is predominantly attributed to the ET at 316 nm from HOMO (O119) to LUMO (O120), and that at 269 nm is mainly contributed by ET at 279 nm from orbital O118 to O121. The high amplitude positive CE at 233 nm is contributed by the ETs at 239 (O118→O124 and O125), 238 (O116→O120 and O121), and 237 (O117→O120) nm. Noticeably, only the ET at 239 nm partially relates to the C-Cl antibonding orbital O125, indicative of the inability to differentiate the (11*R*)- and (11*S*)- configurations by ECD spectroscopy. However, the NOESY correlation between H-11 and H-24β supports an (11*S*)- configuration. This assignment is confirmed by the fact that the H-11-H-24β distances were optimized as 2.06 and 3.95 Å for the (11*S*)- and (11*R*)- configurations, respectively, at the B3LYP/6-311++G** levels in the gas phase (Table SI 4). Additionally, the calculated total nuclear spin-spin coupling constant *J* values also support the (11*S*)- configuration. The *J* values for H-11/H-12α and H-11/H-12β with

the (11*S*)- configuration were calculated as 11.7 and 6.4 Hz, consistent with the experimentally observed values of 10.8 and 6.4 Hz, respectively, whereas those for the (11*R*)- configuration were computed as 4.4 and 2.0 Hz, respectively, at the mPW1PW91-SCRF(PCM)/6-311++G**//B3LYP/6-311++G** level in acetonitrile.[11] Therefore the AC of caulamidine A can be unambiguously assigned as (10*S*,11*S*,23*S*). The experimentally observed CE at 214 nm is contributed by the ETs at 228, 226, 221, 217, 205, 204, and 201 nm (Table SI 3 and Figure SI 3).

ECD computation was also carried out to assign the AC of caulamidine B (Figure SI 1), using the same protocols. As analyzed above, the diagnostic CEs in the ECD spectrum of caulamidine A are generally contributed by the ETs from ${}_{10}^{13}\pi$ to ${}_{10}^{13}\pi^*$, in which some of the 13 electrons are rarely delocalized onto the chlorine atoms. Thus, it may be assumed that the presence of the bromine atoms in caulamidine B wouldn't significantly change the shape of the ECD curve. Since the experimental ECD curve of caulamidine B is highly similar to that of caulamidine A, the AC of caulamidine B was mandatorily assigned as (10*S*,11*S*,23*S*)-. This was confirmed by the excellent agreement of the calculated ECD spectrum of (10*S*,11*S*,23*S*)- caulamidine B with its experimental ECD spectrum (Figure SI 4).

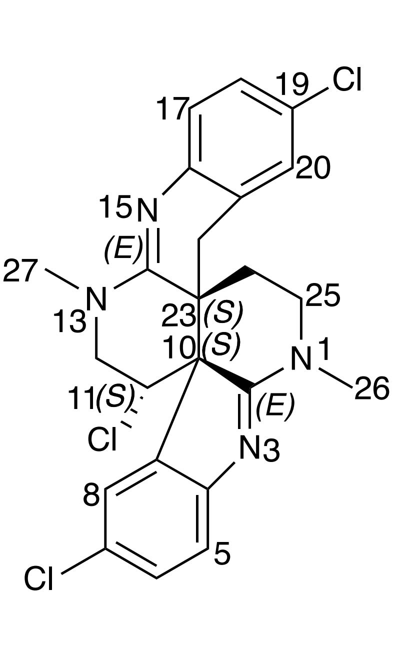

caulamidine A

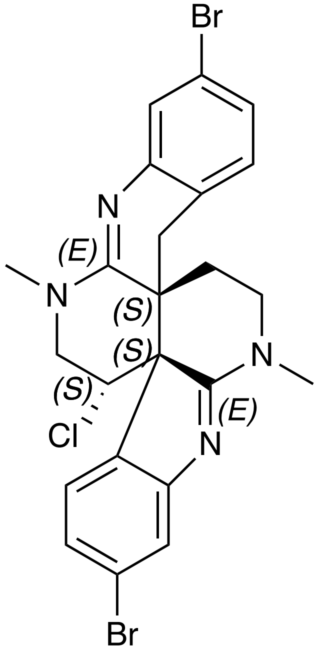

caulamidine B

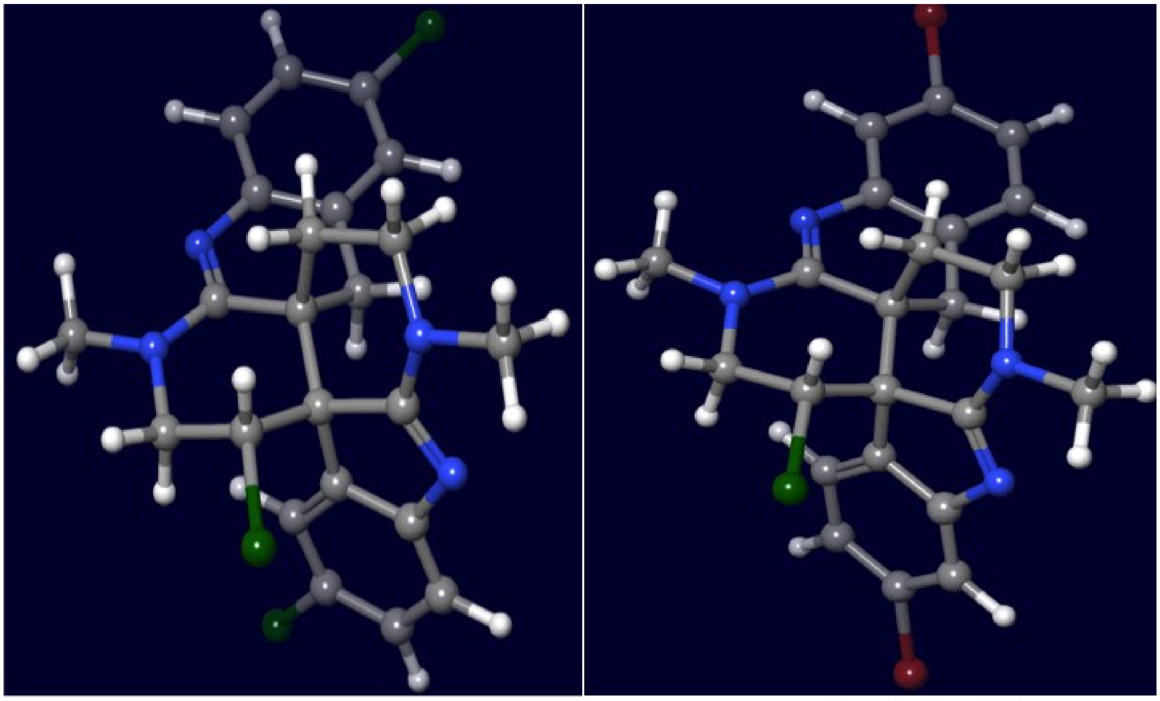

caulamidine A caulamidine B

Figure SI 1. Optimized geometries of (10*S*,11*S*,23*S*)- caulamidines A (**1**) and B (**2**) at the B3LYP/6-311G++ level in the gas phase.

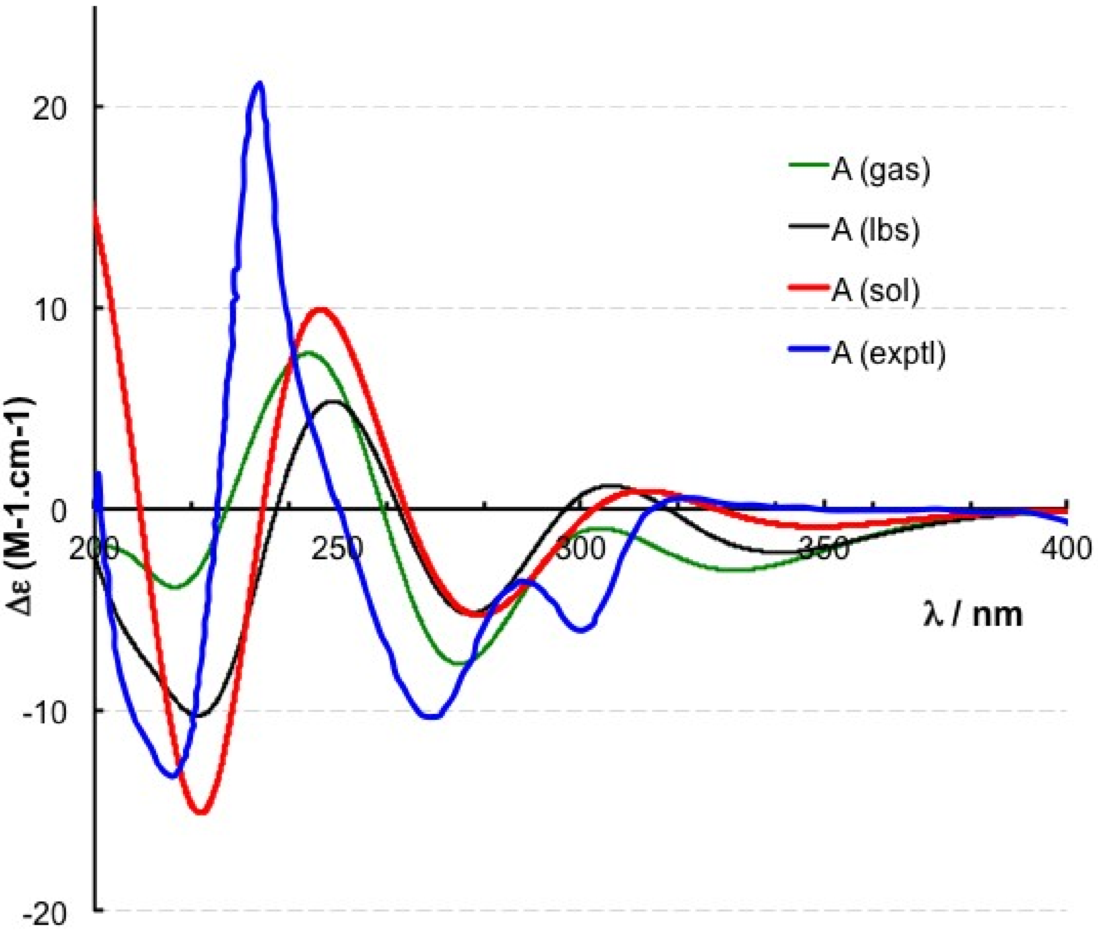

Figure SI 2. Experimental (exptl) and computed ECD spectra of (10*S*,11*S*,23*S*)- caulamidine A at the B3LYP/6-31G** (gas) and B3LYP/6-311++G** (lbs) levels in the gas phase and at the B3LYP-SCRF(COSMO)/6-311++G**//B3LYP/6-311++G** (sol) level in MeOH.

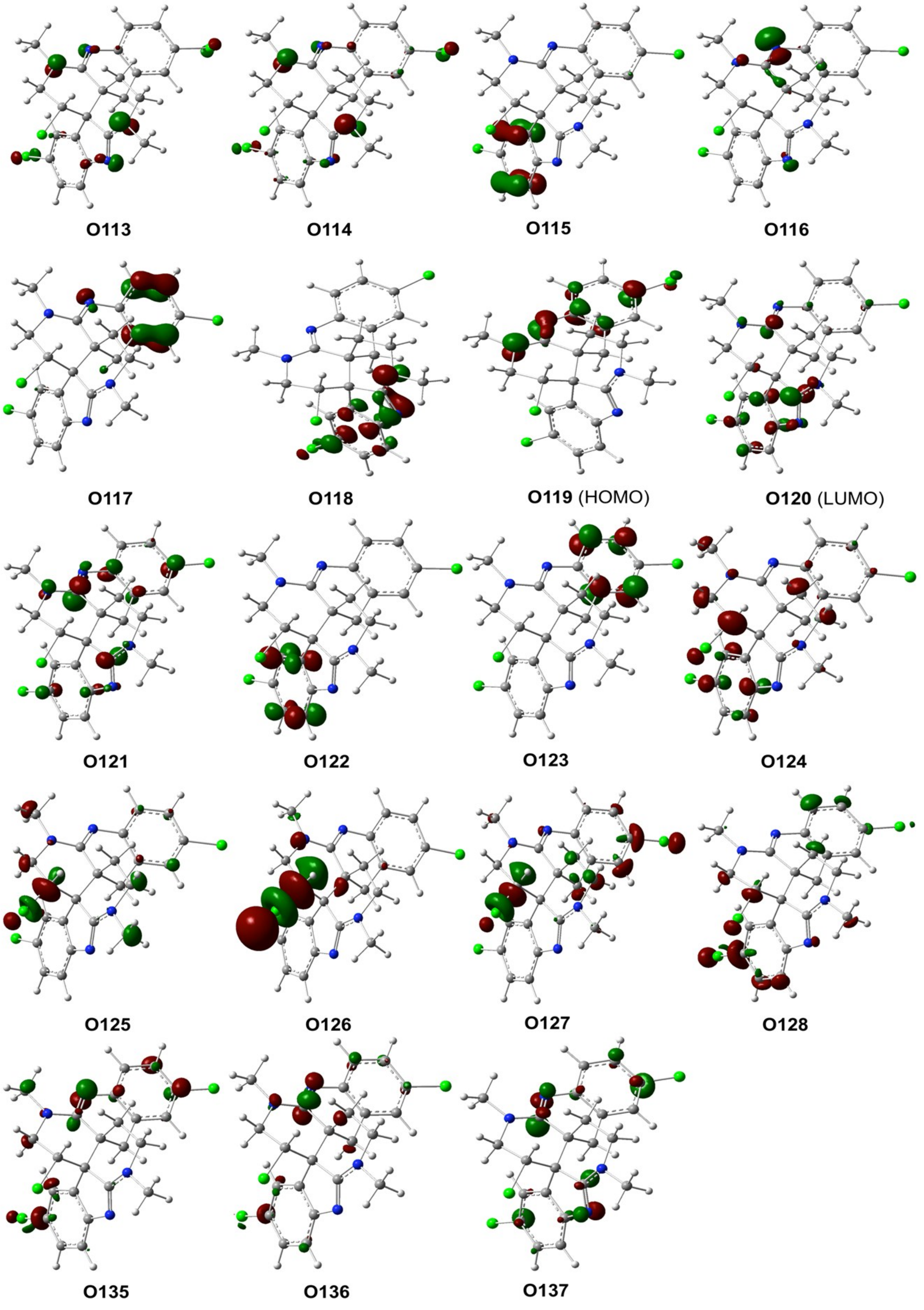

Figure SI 3. Molecular orbitals involved in key transitions in the calculated ECD spectrum of (10*S*,11*S*,23*S*)- caulamidine A at the B3LYP-SCRF(COSMO) /6-311++G**//B3LYP/6-311++G** level in MeOH.

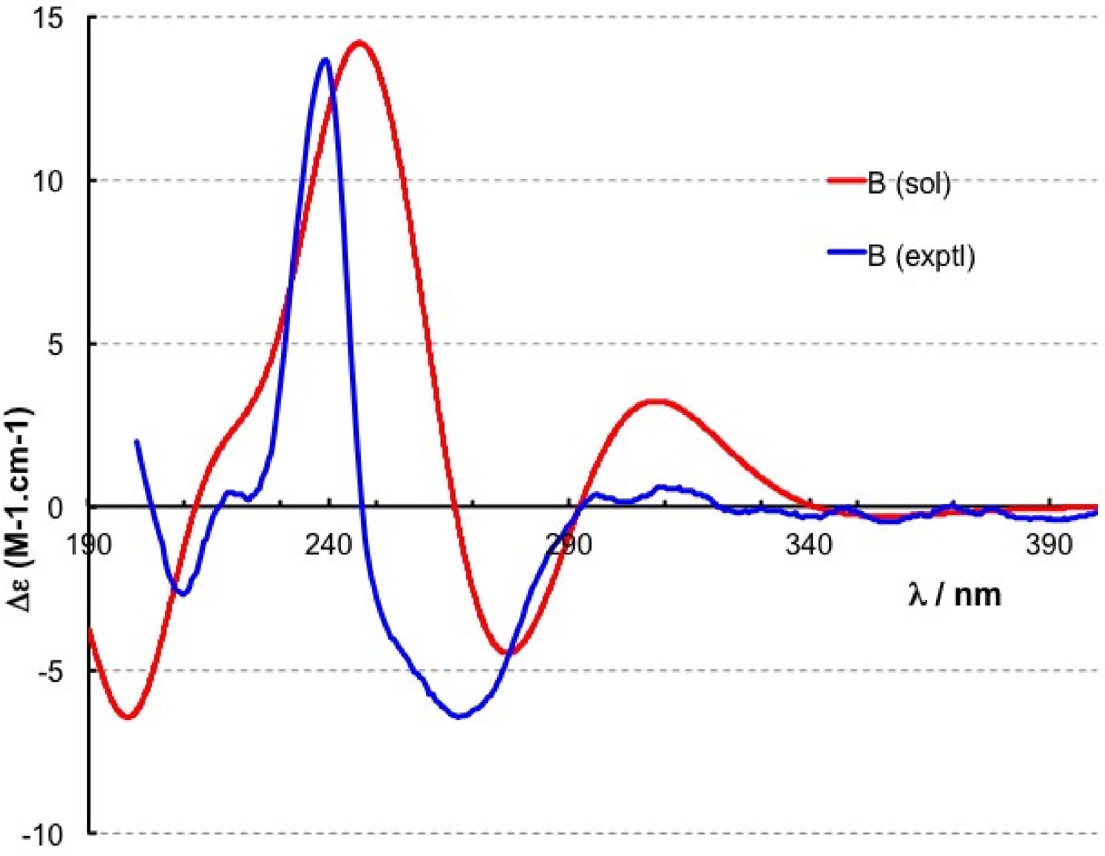

Figure SI 4. Experimental (exptl) and computed ECD spectrum of (10*S*,11*S*,23*S*)- caulamidine B at the B3LYP-SCRF(COSMO)/6-311++G**//B3LYP/6-311++G** (sol) level in MeOH.

Table SI 3. Calculated Transition States, Related Excitation Energies (E), Wave Lengths (λ), Oscillator Strengths (f) and Rotatory Strengths in Length Form (R_{len}) of (10*S*,11*S*,23*S*)- caulamidine A (**1**) at the B3LYP-SCRF(COSMO)/6-311++G**//B3LYP/6-311++G** Level in MeOH.

Excited State		E/ev	λ/nm	f	R_{len}
State#	Related Orbitals				
1	119→120	3.92	316.0	0.12	-77.5
2	118→120	4.01	309.3	0.13	99.7
4	119→122, 119→123	4.38	283.4	0.24	-40.2
5	119→122, 119→123	4.42	280.7	0.01	-17.9
6	118→122	4.44	279.1	0.15	81.2
7	118→121	4.45	278.6	0.17	-78.7
13	118→124, 118→125	5.18	239.4	0.02	40.3
14	116→120, 116→121	5.21	237.8	0.02	52.8
16	117→120	5.24	236.7	0.17	34.8
20	119→128	5.43	228.4	0.01	-31.7
22	115→120, 118→126	5.48	226.1	0.05	-54.4
29	113→120, 118→127	5.62	220.6	0.03	-60.3
32	117→122	5.72	216.9	0.05	-75.0
47	114→122, 119→137	6.06	204.7	0.07	-66.7
49	118→136/5	6.08	203.9	0.03	-29.6
52	115→123, 119→137	6.16	201.4	0.08	-31.4

Table SI 4. Important Interatomic Distances in the Geometries of (10*S*,11*S*,23*S*)-caulamidines A (**1**) and B (**2**) Optimized at the B3LYP/6-311++G** Level in the Gas Phase (Å).

Distance	A	B
H-5 to H-6	2.50	-
H-5 to Me-26	4.81	4.79
H-7 to H-8	-	2.47
H-8 to H-12α	2.31	2.31
H-8 to H-22α	2.93	2.94
H-8 to Me-27	3.46	3.47
H-11 to H-12β	2.41	2.41
H-11 to H-12α	3.05	3.05
H-11 to H-24β	2.06	2.05
H-11 to H-24α	3.63	3.62
H-12β□to Me-27	2.23	2.23
H-12α to Me-27	2.90	2.91
H-17 to Me-27	3.77	3.76
H-17 to H-18	2.49	-
H-19 to H-20	-	2.48
H-20 to H-22β	2.51	2.51
H-20 to H-22α	3.24	3.24
H-22β□ to H-24α	2.98	2.98
H-22β□ to H-25α	2.36	2.37
H-24α□to H-25α	2.38	2.38
H-24α□to H-25β	2.54	2.54
H-24β□to H-25β	2.38	2.38
H-24β□to H-25α	3.05	3.05
H-25β□to Me-26	2.40	2.40
H-25α□to Me-26	2.60	2.61

Table SI 5. Comparison of the DFT-calculated and experimentally measured ^{13}C NMR chemical shift values for caulamidine A (**1**) and caulamidine B (**2**) in CD_3CN.

Position	**1**		**2**	
	DFT-calculated ^{13}C shift (ppm)	Observed ^{13}C shift (ppm)	DFT-calculated ^{13}C shift (ppm)	Observed ^{13}C shift (ppm)
2	173.8	174.0	174.5	174.4
4	156.9	156.0	159.3	159.1
5	120.4	117.8	123.4	119.7
6	130.2	129.4	122.7	122.5
7	127.9	126.3	124.7	123.8
8	123.6	123.8	123.5	125.1
9	133.2	133.3	130.3	130.4
10	57.9	58.9	57.1	58.4
11	54.9	54.8	54.7	54.7
12	54.0	52.6	53.9	52.7
14	156.8	159.1	157.5	159.7
16	143.9	143.9	146.4	146.9
17	125.6	124.2	127.6	125.2
18	128.1	127.2	119.6	120.2
19	128.2	125.8	125.9	124.3
20	126.9	127.3	127.7	129.2
21	123.9	125.4	121.4	122.5
22	30.5	29.6	30.1	29.3
23	41.3	39.8	41.5	39.6
24	26.9	24.7	25.8	24.6
25	49.0	47.4	49.0	47.4
26-Me	38.9	37.2	38.8	37.2
27-Me	37.7	35.8	36.8	35.7

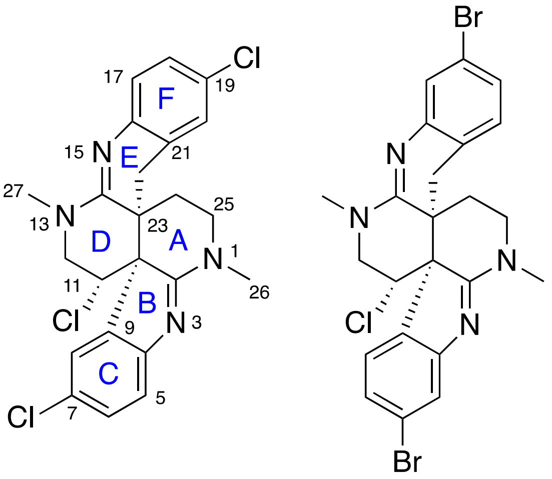

1 **2**

Table SI 6. Experimental RDC data of caulamidine A (1)

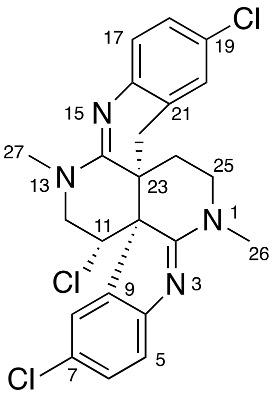

Bond	RDC (Hz)
C23-H23	6.9
C24-H24	8.1
C6-H6	-1.9
C4-H4	9.4
C1-H1	9.1
C21-H21	6.9
C26-H26	-4
C28-H28a/b	overlap with gel signal
C14-H14a/b	overlap with gel signal
C9-H9a/b*	0.2
C15-H15a/b	0.5
C29-H29a/b/c†	-0.7
C17-H17a/b/c	-0.2

* Methylene RDCs are reported as the averages of the two individual CH RDCs.

† Methyl group RDCs are utilized in analogy to previously described analysis,[12,13], except that a C-H to C-N conversion factor of 6.3^{-1} was used specifically for the N-methyl.

Table SI 7. Experimental RCSA data of caulamidine A (1)

Atom	RCSA*† (Hz)
C12	-0.2
C8	-1.5
C19	-0.3
C2	-2.6
C20	-1.5
C23	-2.1
C4	-3.4
C6	-2.5
C22	3.5
C3	0.1
C5	-2.3
C1	-1.8
C21	-1.4
C24	-2
C10	overlap with gel signal
C26	0.1
C28	-0.5
C14	1.7
C11	overlap with gel signal
C17	0.1
C29	0.4
C9	0.8
C15	0.5

* Resonances are first referenced relative to TMS (tetramethylsilane) at 0 ppm. In order to compensate for a potential referencing error due to TMS evaporation during the relatively lengthy NMR measurements, the strong alignment spectrum was further shifted upfield by 0.5 Hz relative to the weak alignment spectrum, on the basis of a slightly improved Q-factor.

† Values in Hz are based on a spectrometer frequency of 500 MHz.

The $^{35}Cl/^{37}Cl$ isotope effect detected by bs-HSQC for C-11 of caulamidine B (2).

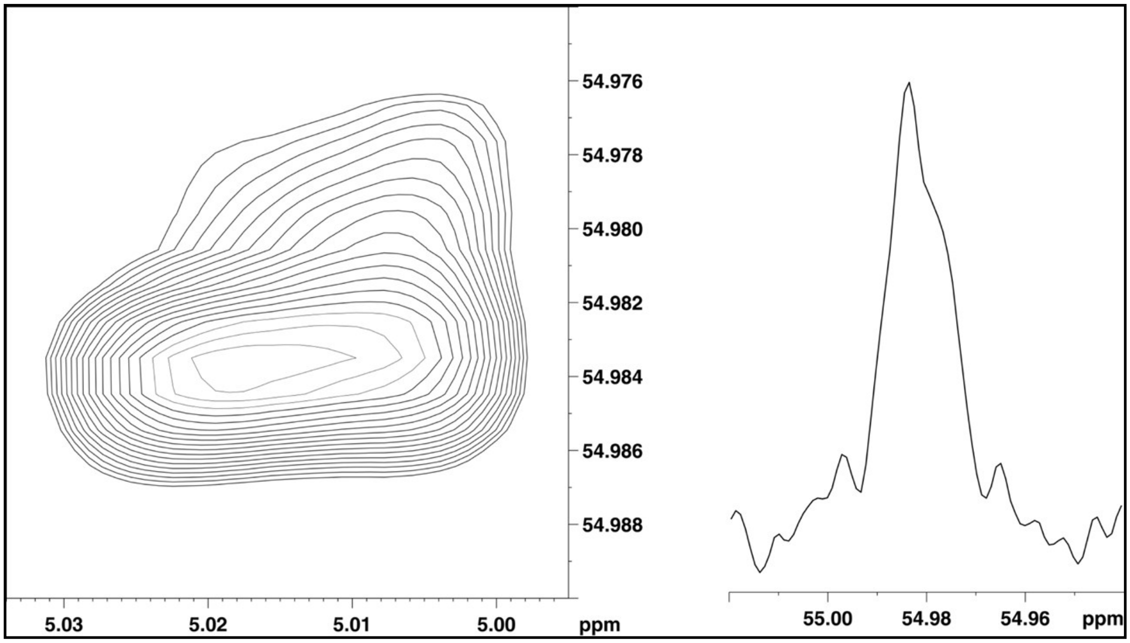

Antimalarial Screening Assay

The antimalarial activity was determined against chloroquine sensitive (D6) and chloroquine resistant (W2) strains of *Plasmodium falciparum* by measuring plasmodial lactate dehydrogenase (LDH) activity according to the procedure of Makler and Hinrichs.[14] A suspension of red blood cells infected with the D6 or W2 strain of *P. falciparum* (200 μL, with 2% parasitemia and 2% hematocrit in RPMI 1640 medium supplemented with 10% human serum and 60 μg/mL Amikacin) was added to the wells of a 96- well plate containing 10 μL of serially diluted test samples. The plate was incubated at 37 ºC, for 72 h in an environment of 90% N_2, 5% O_2, and 5% CO_2. Plasmodial LDH activity was determined by mixing 20 μL of the incubation mixture with 100 μL of the Malstat reagent and incubating at room temperature for 30 min. Twenty microliters of a 1:1 mixture of NBT/PES (Sigma, St. Louis, MO) was added and the plate was further incubated in the dark for 1 h. The reaction was then stopped by adding 100 μL of a 5% acetic acid solution and the absorbance was read at 650 nm. Artemisinin and chloroquine were included as the drug controls. The *in vitro* cytotoxicity of samples to mammalian cells was also tested to determine the selectivity index of the antimalarial activity. Vero cells (monkey kidney fibroblasts) were seeded into a 96-well plate at a density of 25,000 cells/well and grown for 24 h. Test samples at different concentrations were added and cells were further incubated for 48 h. Cell viability was determined by the Neutral Red method.[15] Doxorubicin was included as the drug control. IC_{50} values were obtained from the dose response curves.

	***P. falciparum* strain**		**Cytotox IC_{50} Vero cells**
**Sample**	**D6 (IC_{50} μM)**	**W2 (IC_{50} μM)**	
Caulamidine A	11.3	8.3	NC
Caulamidine B	12	12.9	NC
Chloroquine	0.02	0.37	
Artemisinin	0.03	0.02	

NC= no cytotoxicity at 50 μM

Supporting Information References

(1) Gil-Silva, L.F.; Santamaría-Fernández, R.; Navarro-Vázquez, A.; Gil, R.R. *Chemistry Eur. J*, **2016**, 472-476.
(2) Nath, N.; Schmidt, M.; Gil, R.R; Williamson, R.T.; Martin, G.E.; Navarro-Vázquez, A.; Griesinger, C.; Liu, Y. *J. Am. Chem. Soc.* **2016**, 138, 9548-9556.
(3) Castañar, L.; García,M.; Hellemann, E.; Nolis, P.; Gil, R.R.; Parella, T. *J. Org. Chem*. **2016**, 81, 11126-11131.
(4) Bokesch, H. R.; Blunt, J. W.; Westergaard, C. K.; Cardellina II, J. H.; Johnson, T. R.; Michael, J. A.; McKee, T. C.; Hollingshead, M. G.; Boyd, M. R. *J. Nat. Prod*. **1999**, *62*, 633-635.
(5) Ding, Y.; Li, X.-C.; Ferreira, D. *J. Org. Chem.* **2007**, *72*, 9010-9017.
(6) Ding, Y.; Li, X.-C.; Ferreira, D. *J. Nat. Prod.* **2009**, *72*, 327-335.
(7) Ding, Y.; Li, X.-C.; Ferreira, D. *J. Nat. Prod.* **2010**, *73*, 435-440.
(8) Li, C.-S.; Ding, Y.; Yang, B.-J.; Miklossy, G.; Yin, H.-Q.; Walker, L. A.; Turkson, J.; Cao, S. *Org. Lett.* **2015**, *17*, 3556-3559.
(9) MacroModel; version 9.9 ed.; Schrödinger LLC, New York, NY: 2011.
(10) Frisch, M. J.; Trucks, G. W.; Schlegel, H. B.; Scuseria, G. E.; Robb, M. A.; Cheeseman, J. R.; Scalmani, G.; Barone, V.; Mennucci, B.; Petersson, G. A.; Nakatsuji, H.; Caricato, M.; Li, X.; Hratchian, H. P.; Izmaylov, A. F.; Bloino, J.; Zheng, G.; Sonnenberg, J. L.; Hada, M.; Ehara, M.; Toyota, K.; Fukuda, R.; Hasegawa, J.; Ishida, M.; Nakajima, T.; Honda, Y.; Kitao, O.; Nakai, H.; Vreven, T.; Montgomery, J., J. A.; Peralta, J. E.; Ogliaro, F.; Bearpark, M.; Heyd, J. J.; Brothers, E.; Kudin, K. N.; Staroverov, V. N.; Kobayashi, R.; Normand, J.; Raghavachari, K.; Rendell, A.; Burant, J. C.; Iyengar, S. S.; Tomasi, J.; Cossi, M.; Rega, N.; Millam, J. M.; Klene, M.; Knox, J. E.; Cross, J. B.; Bakken, V.; Adamo, C.; Jaramillo, J.; Gomperts, R.; Stratmann, R. E.; Yazyev, O.; Austin, A. J.; Cammi, R.; Pomelli, C.; Ochterski, J. W.; Martin, R. L.; Morokuma, K.; Zakrzewski, V. G.; Voth, G. A.; Salvador, P.; Dannenberg, J. J.; Dapprich, S.; Daniels, A. D.; Farkas, Ö.; Foresman, J. B.; Ortiz, J. V.; Cioslowski, J.; Fox, D. J.; Gaussian 09 Revision A.1; Gaussian Inc., Wallingford CT, 2009.
(11) Li, C.-S.; Ren, G.; Yang, B.-J.; Miklossy, G.; Turkson, J.; Fei, P.; Ding, Y.; Walker, L. A.; Cao, S. *Org. Lett.* **2016**, *18*, 2335-2338.
(12) Ottiger, M.; Bax, A. *J. Am. Chem. Soc*. **1999**, 121, 4690-4695.
(13) Sánchez-Pedregal, V.M.; Santamaría-Fernández, R.; Navarro-Vázquez, A. *Org Lett*., **2009**, 11, 1471-1474.
(14) Makler, M. T.; Hinrichs, D. J. *Am. J. Trop. Med. Hyg.* **1993**, *48*, 205-210.
(15) Borenfreund, E.; Babich, H.; Martin-Alguacil, N. *In vitro Cell. Dev. Biol.* **1990**, *26*, 1030-1034

Caulamidine A (1) ^{1}H NMR Spectrum (600 MHz, CD_3CN)

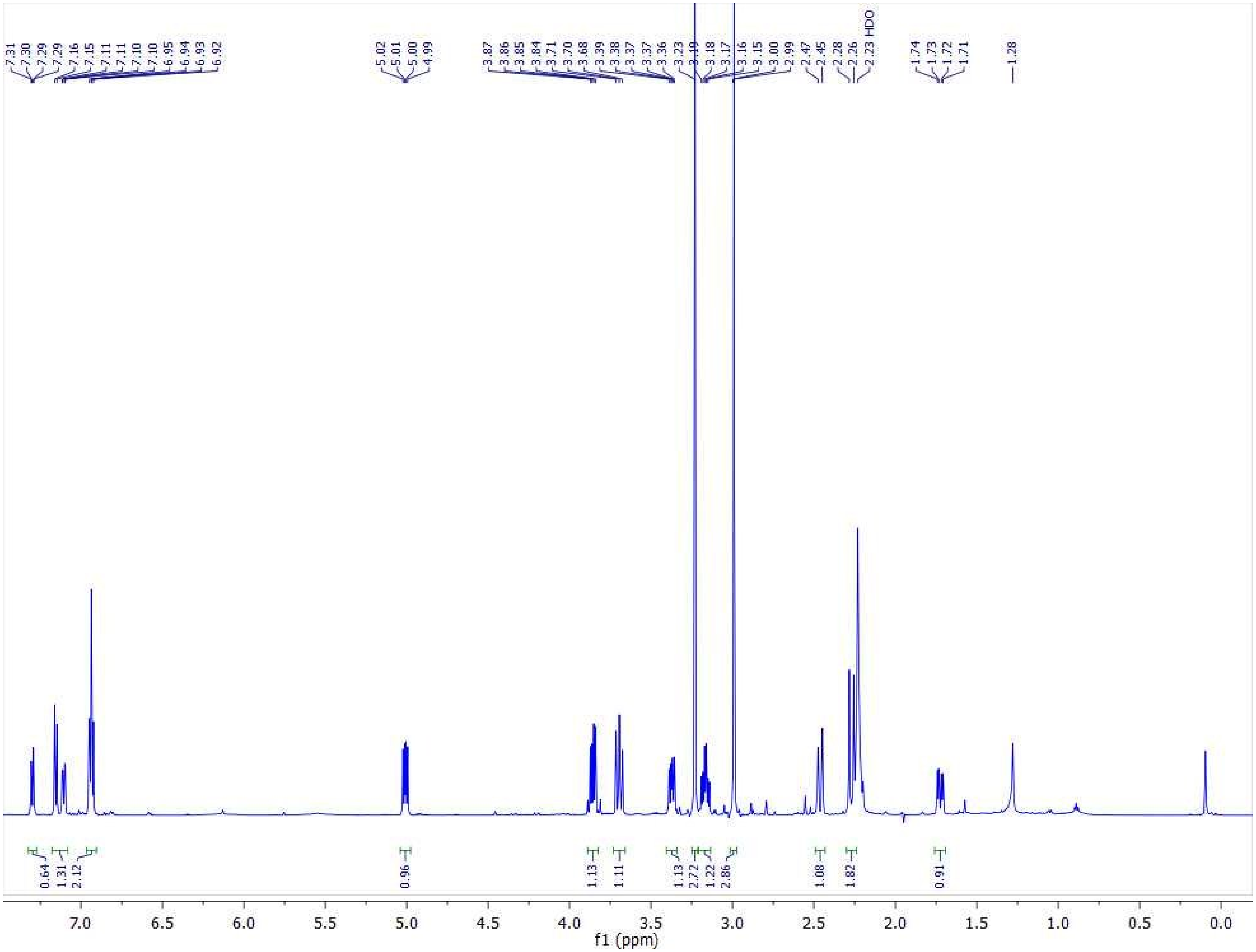

Caulamidine A (1) ^{13}C NMR Spectrum (150 MHz, CD_3CN)

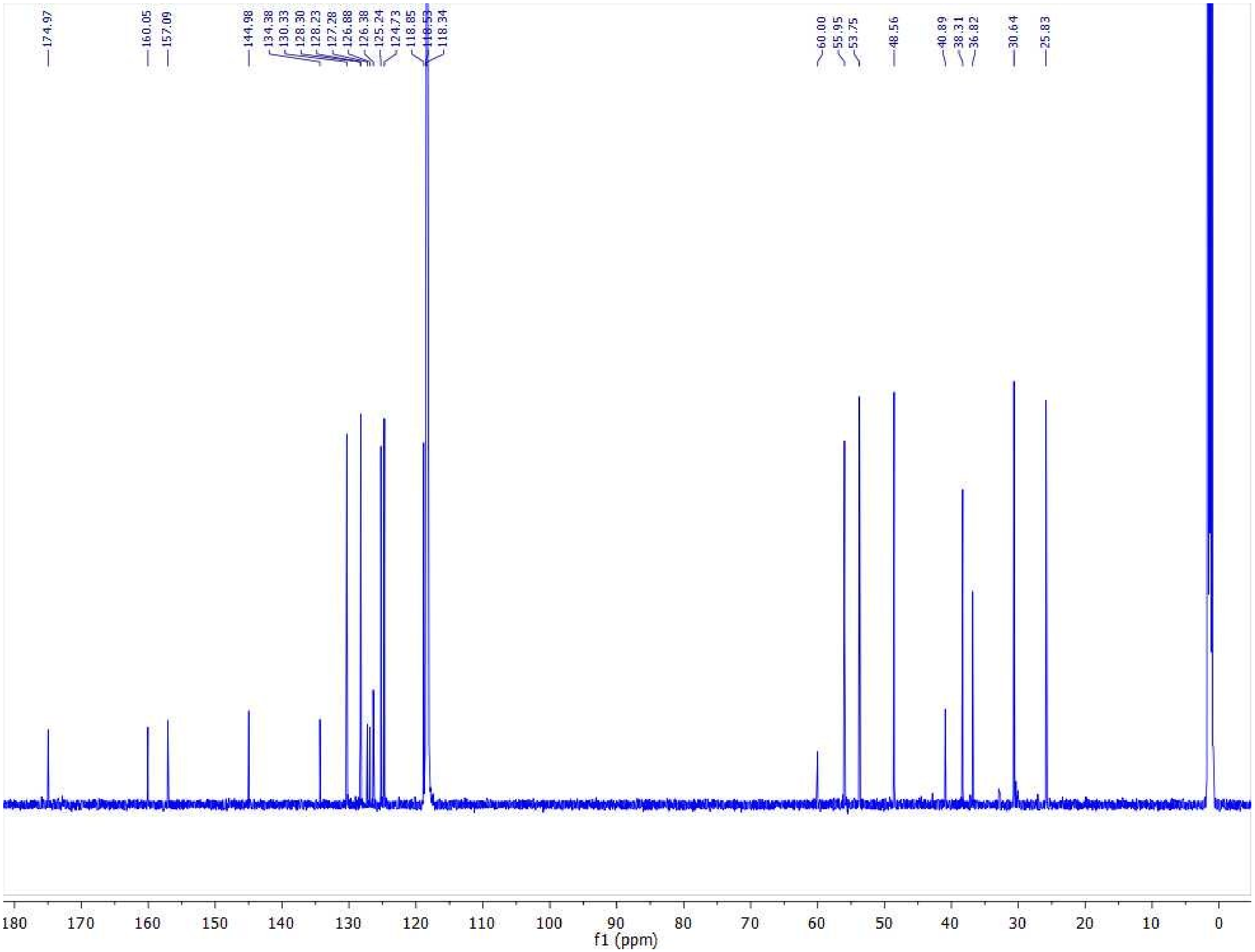

Caulamidine A (1) COSY Spectrum (CD_3CN)

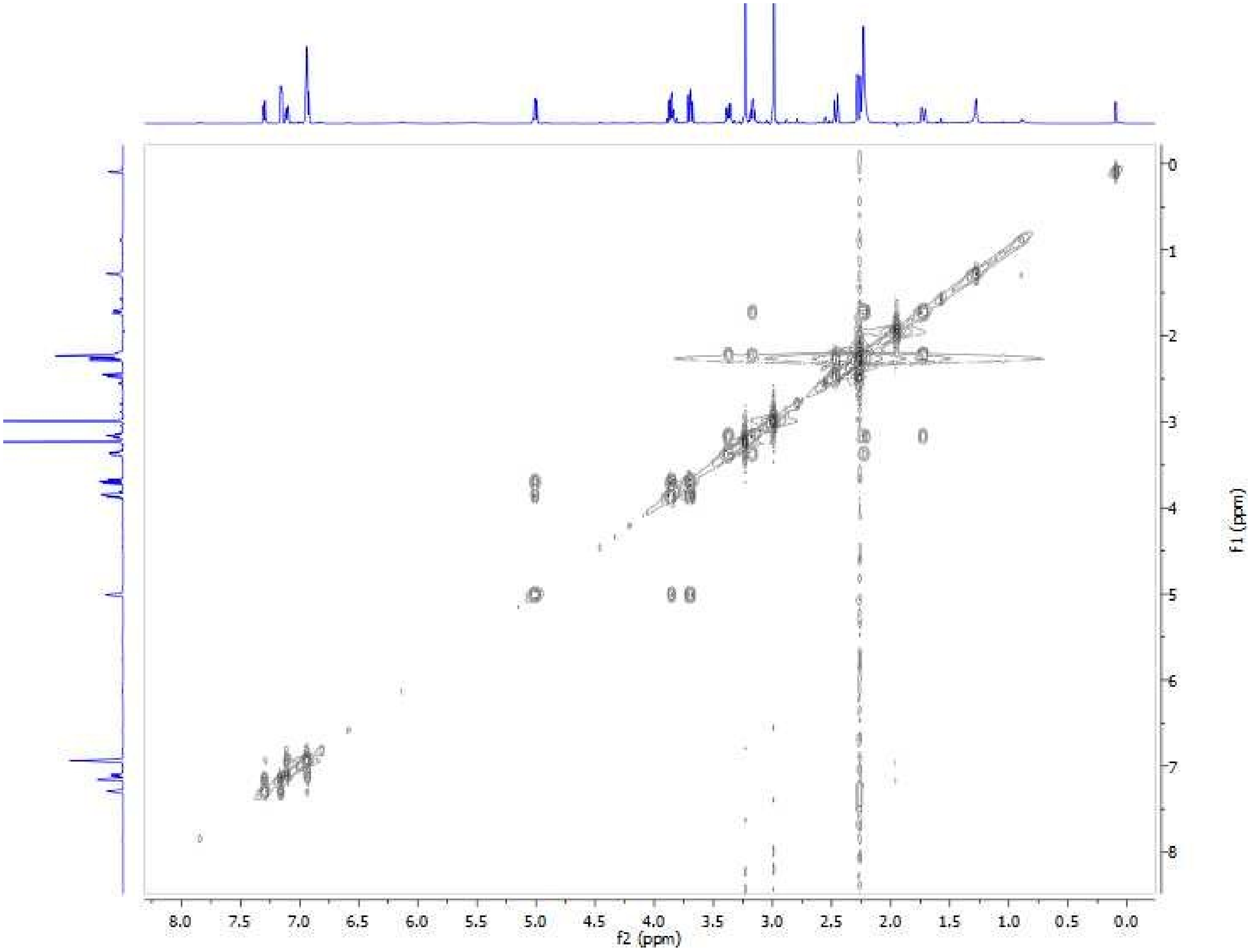

Caulamidine A (1) HSQC Spectrum (CD_3CN)

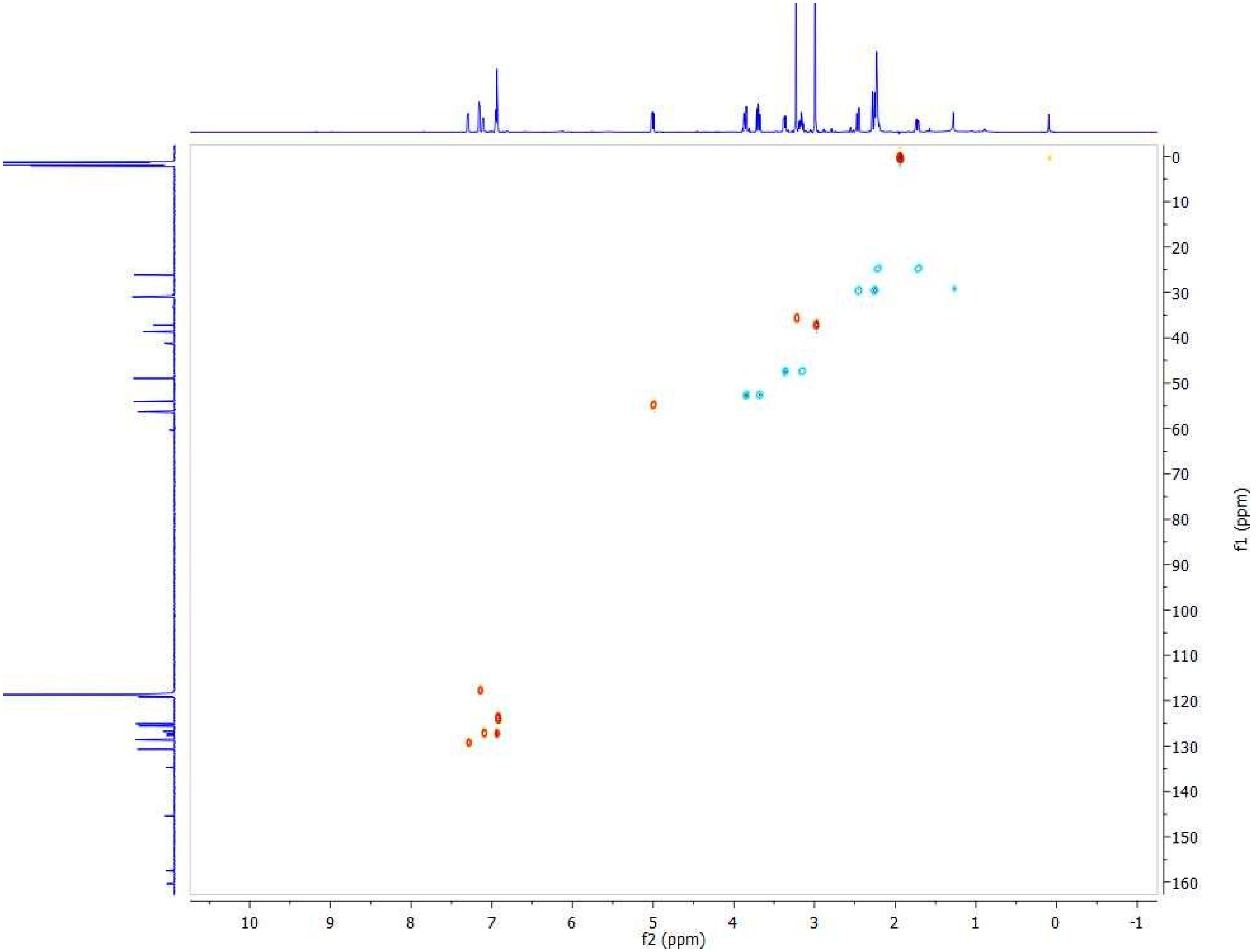

Caulamidine A (1) ^{1}H-^{13}C HMBC Spectrum (CD_3CN) Optimized for 8.3 Hz

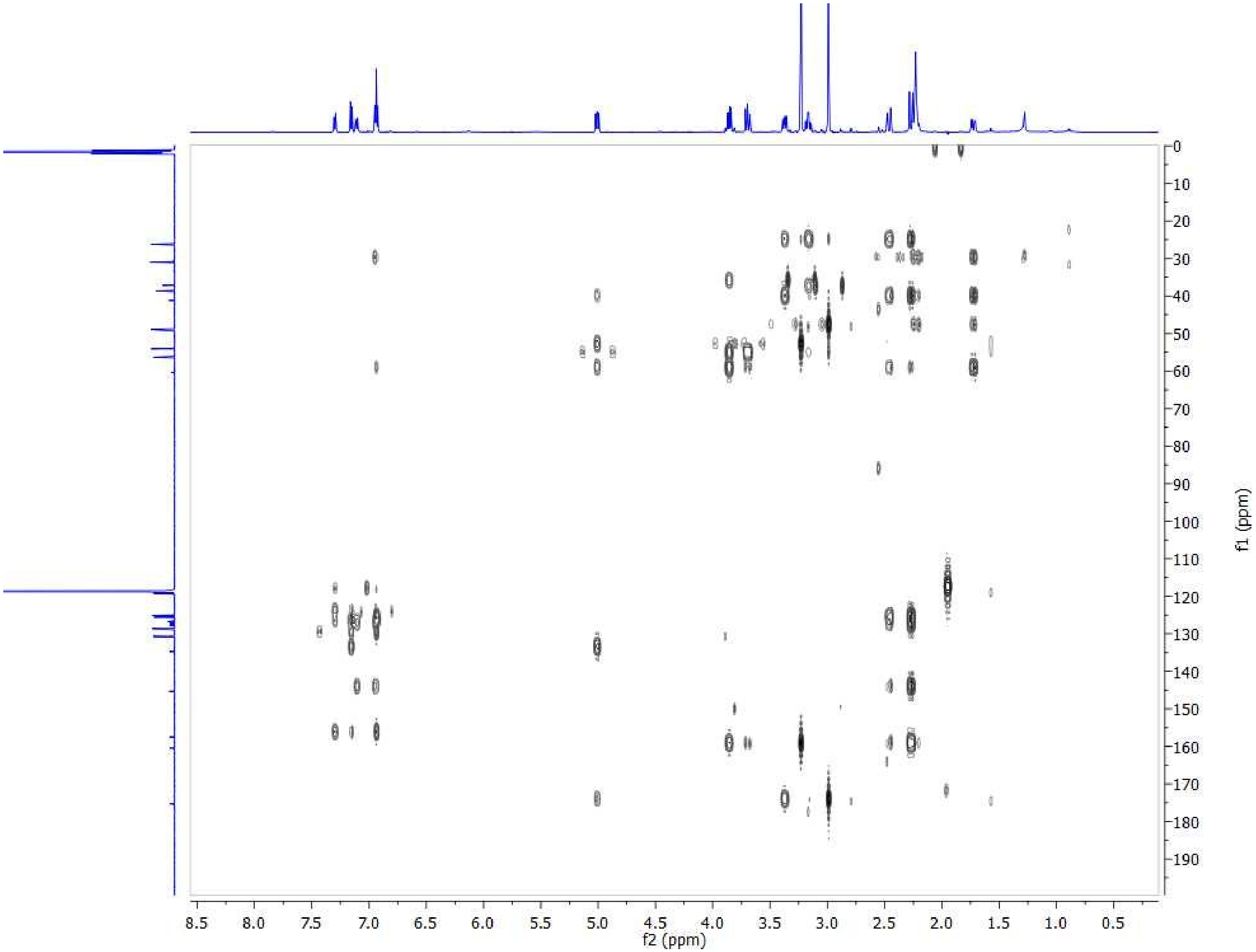

Caulamidine A (1) NOESY Spectrum (CD_3CN)

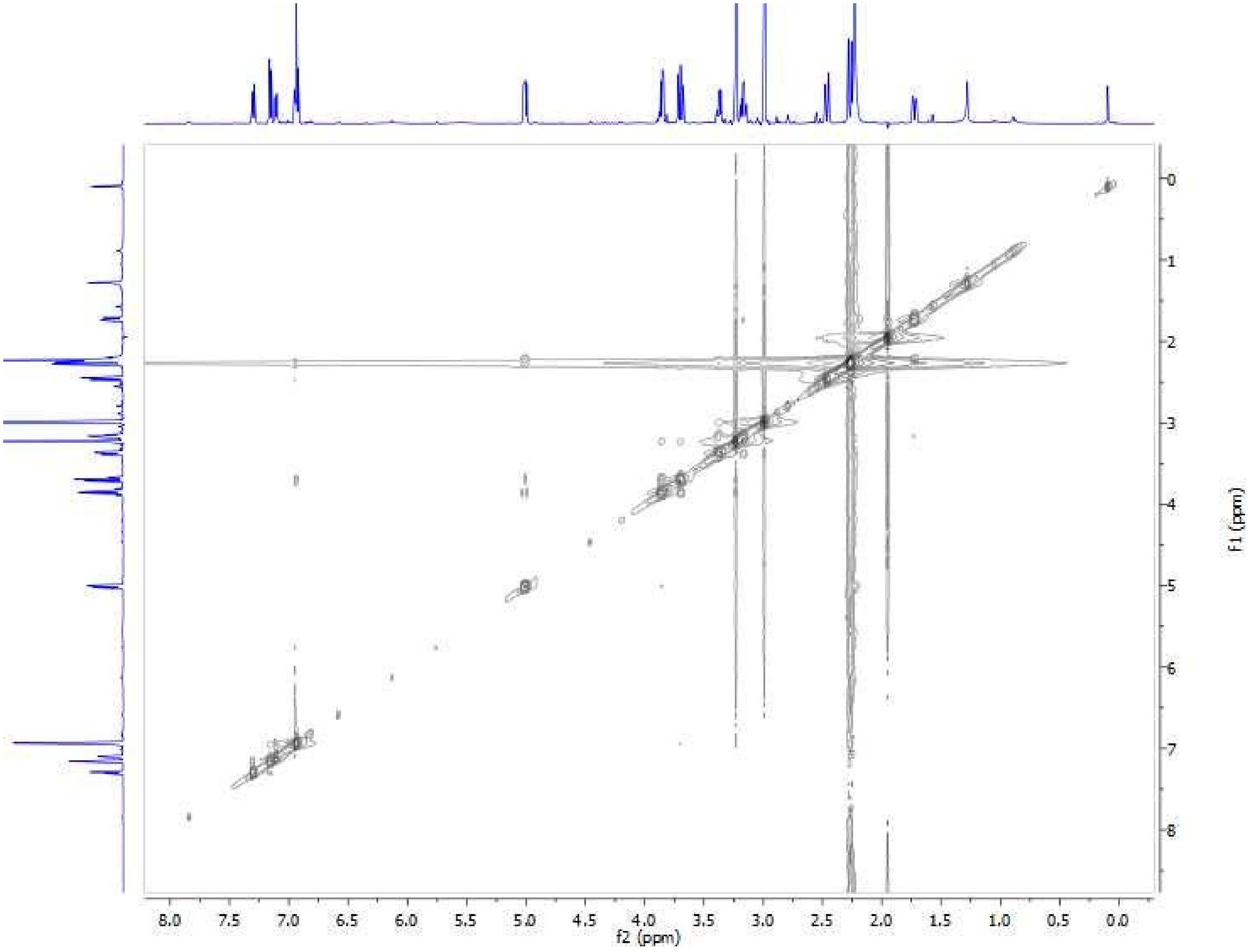

Caulamidine A (1) ROESY Spectrum (600 MHz, CD_3CN)

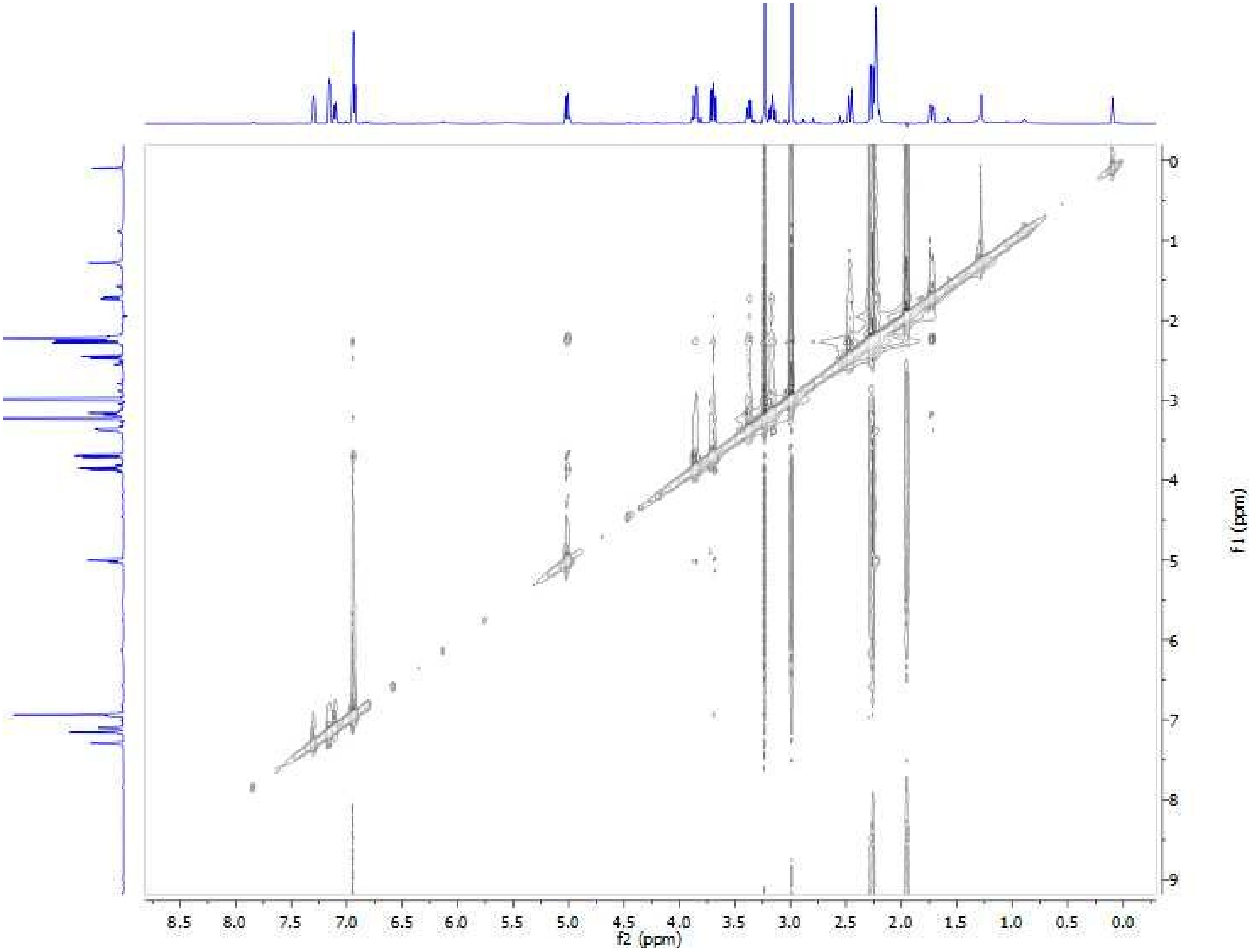

Caulamidine A (1) ^{15}N-^{1}H HMBC Spectrum (CD_3CN) Optimized for 8.0 Hz

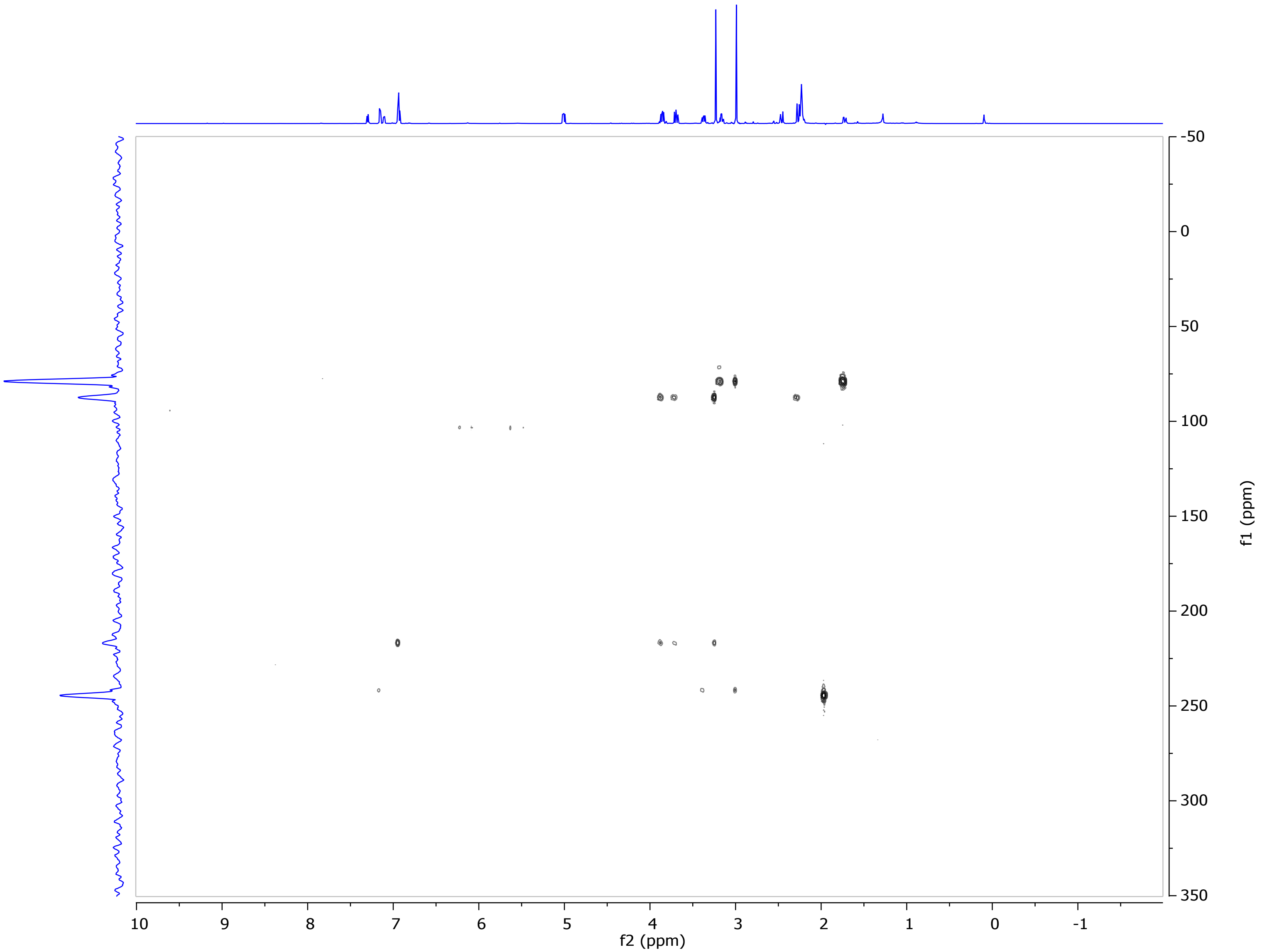

Caulamidine A (1) ^{1}H-^{13}C LR-HSQMBC Spectrum (CD_3CN) Optimized for 2Hz.

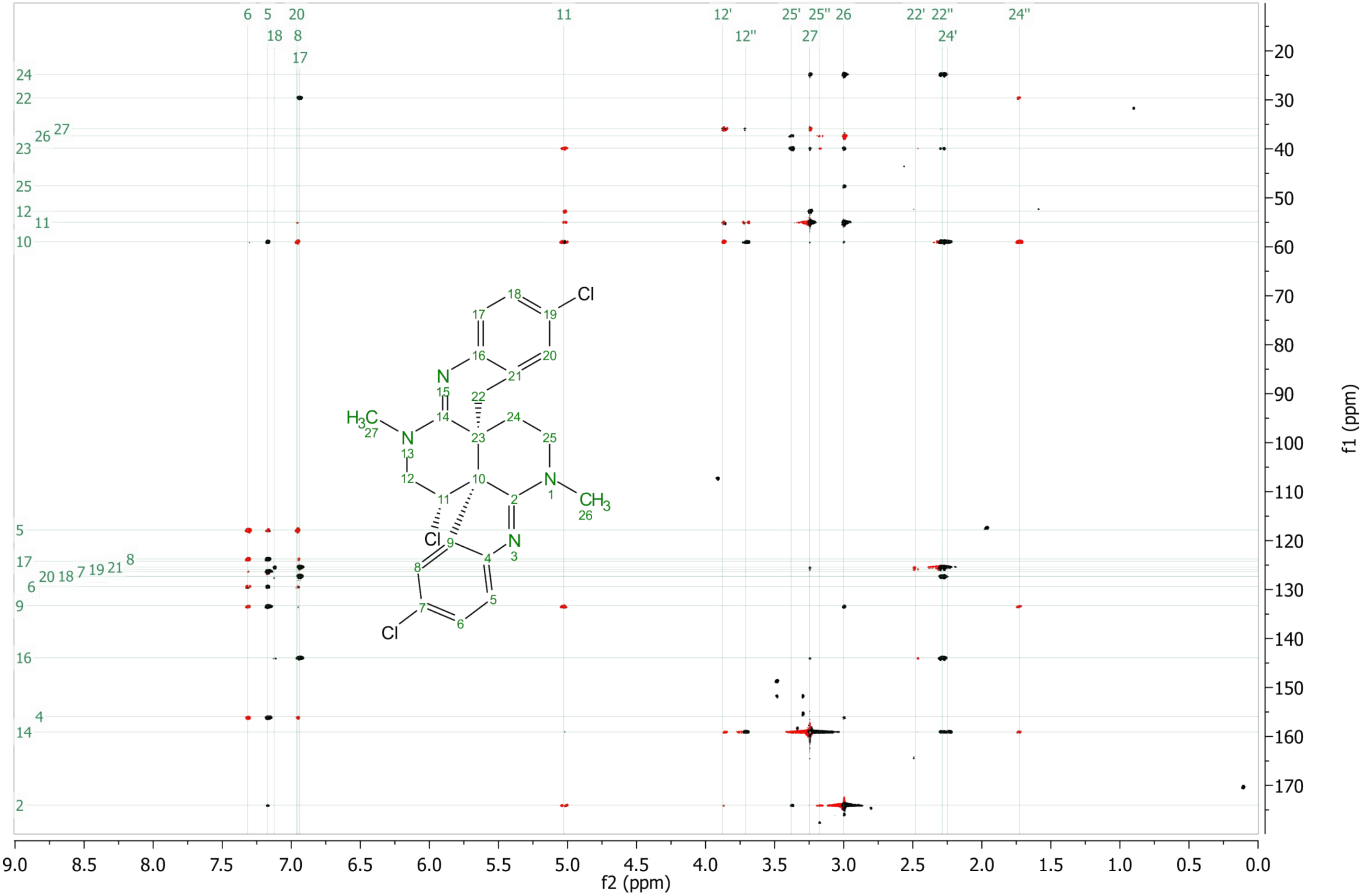

Caulamidine A (1) ^1H-^{13}C HSQMBC-TOCSY Spectrum (CD_3CN) Optimized for 8Hz + 60 ms Mixing Time

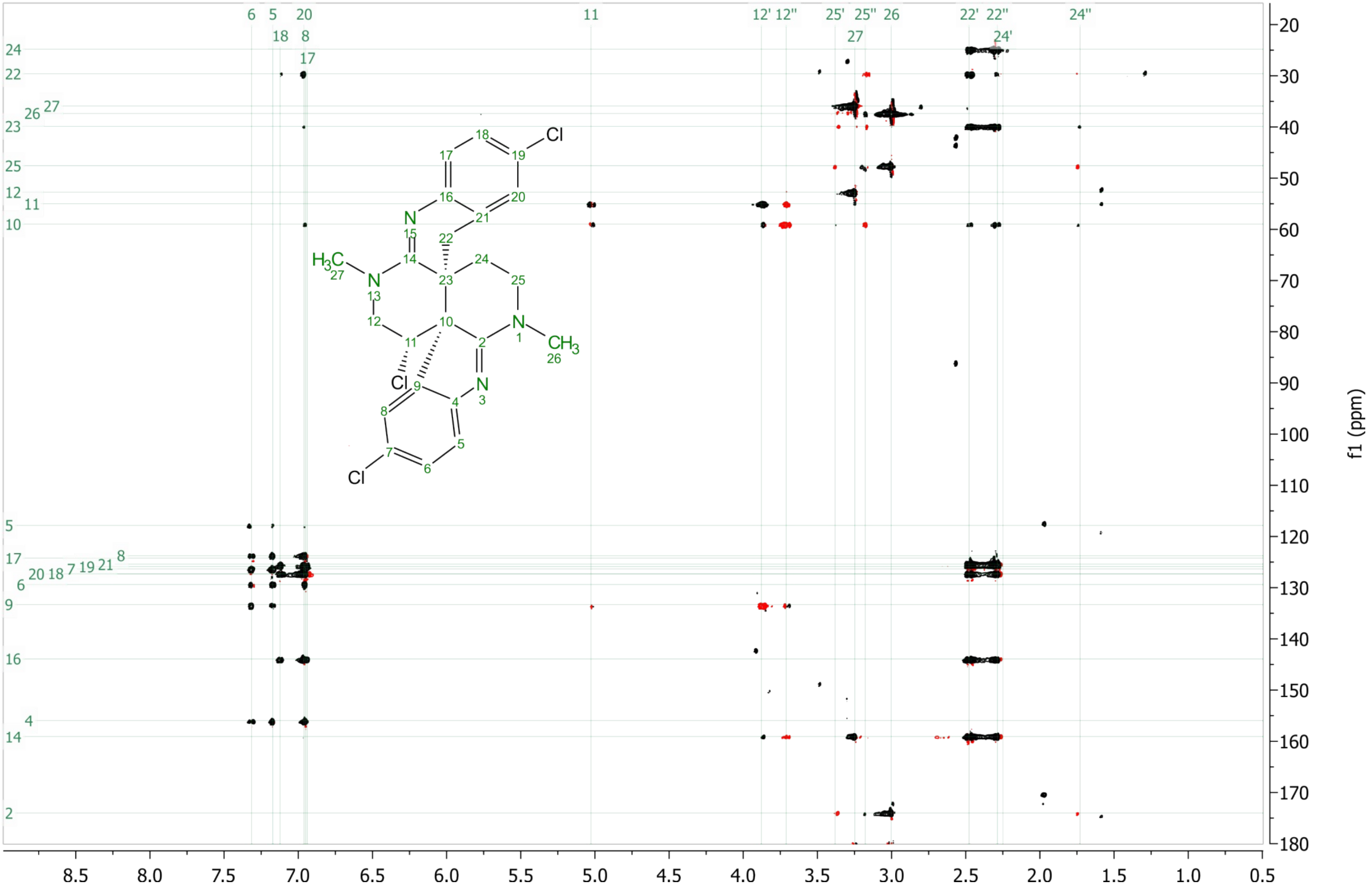

Caulamidine A (1) ^{1}H-^{15}N HSQMBC-TOCSY Spectrum (CD_3CN) Optimized for 4Hz + 40 ms Mixing Time

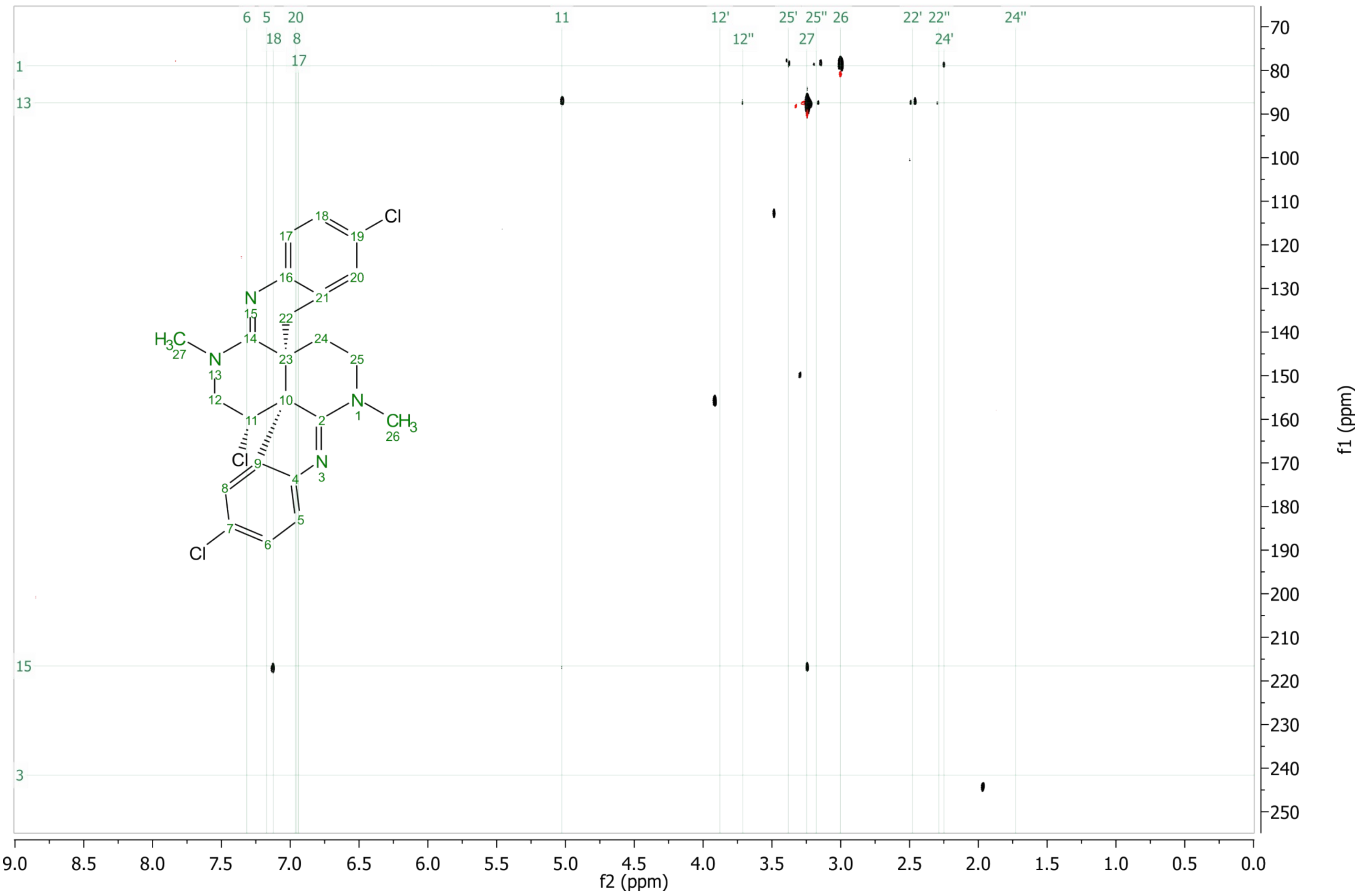

Caulamidine B (2) ^{1}H NMR Spectrum (600 MHz, CD_3CN)

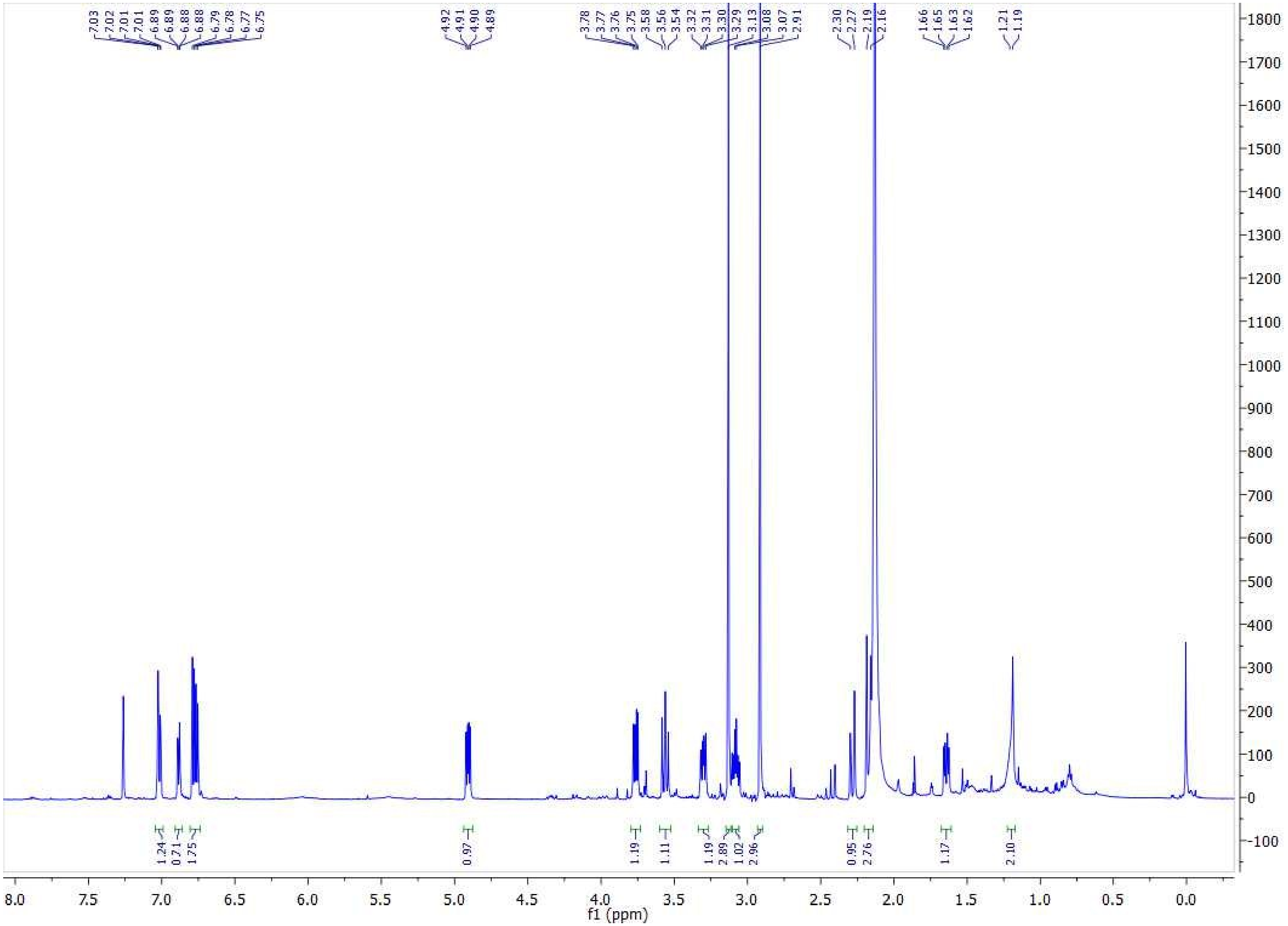

Caulamidine B (2) ^{13}C NMR Spectrum (150 MHz, CD_3CN)

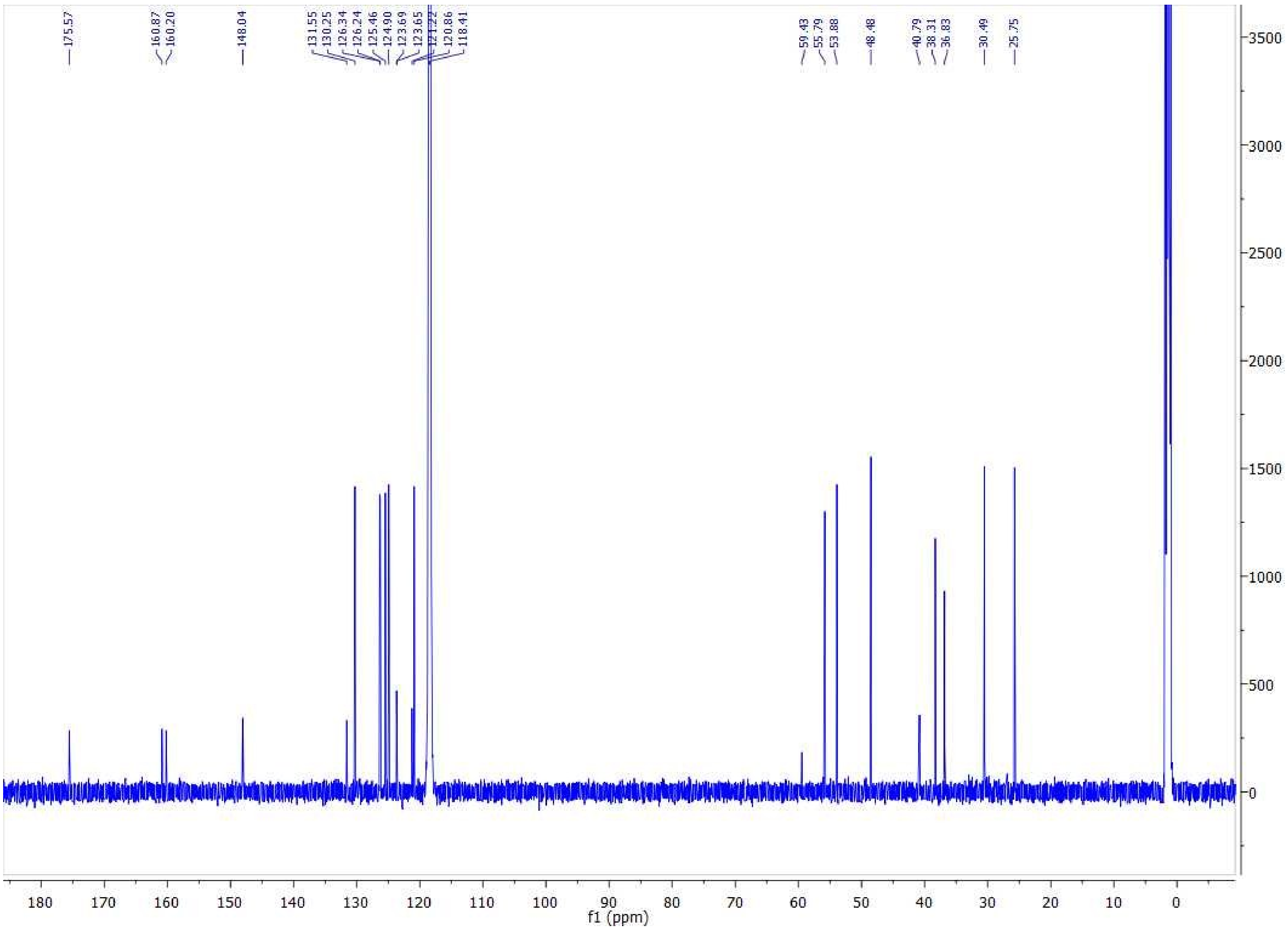

Caulamidine B (2) COSY Spectrum (CD_3CN)

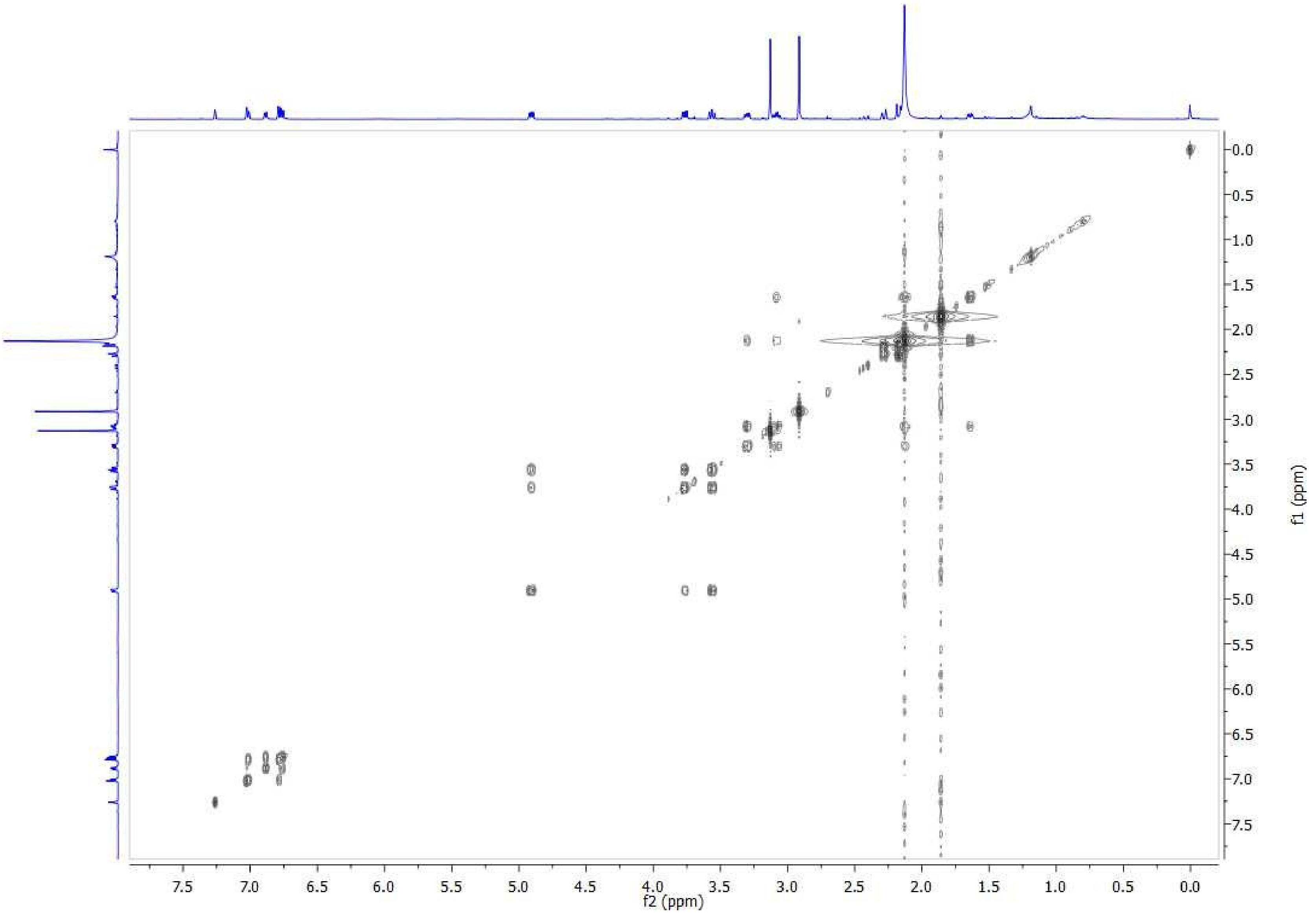

Caulamidine B (2) HSQC Spectrum (CD_3CN)

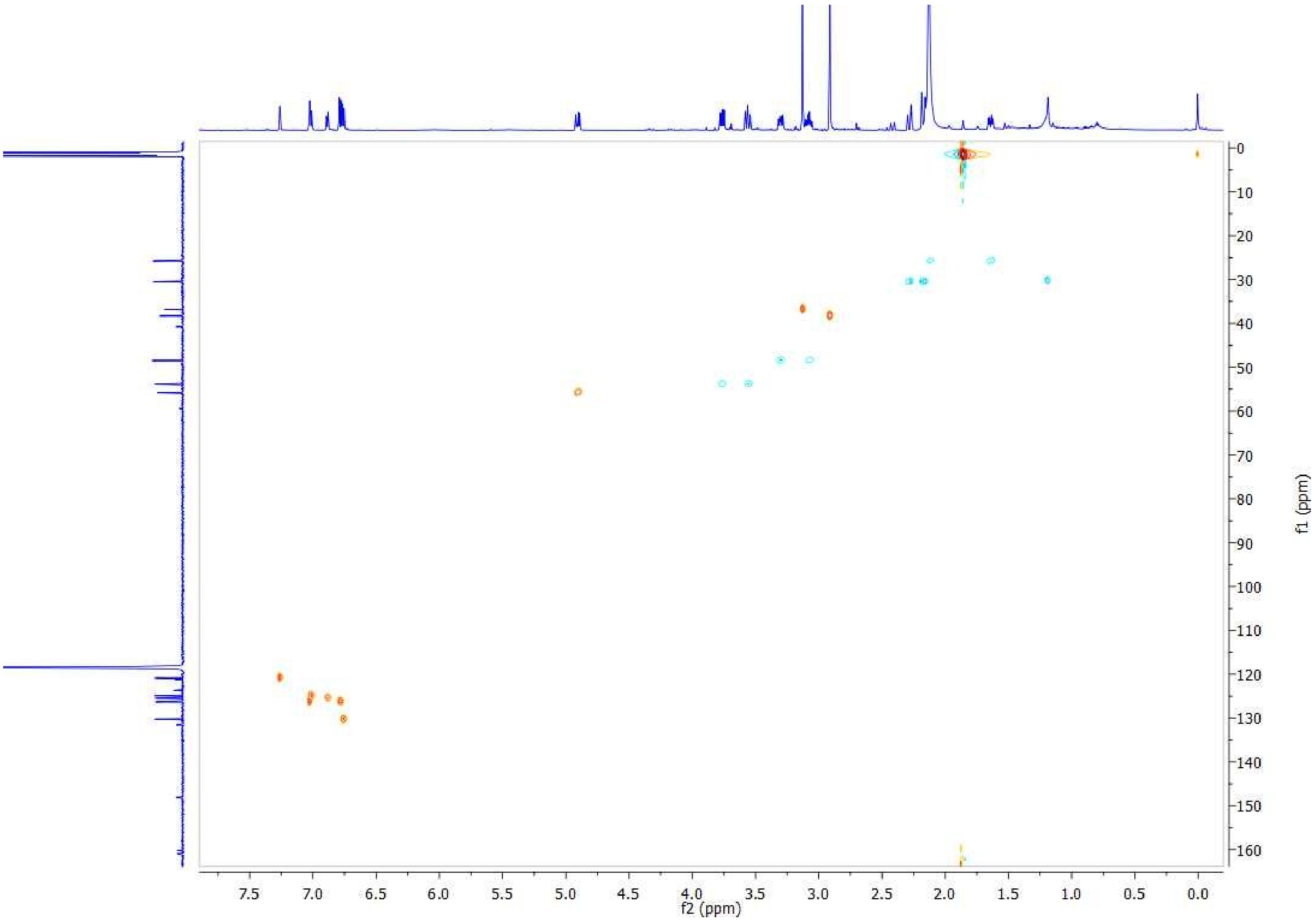

Caulamidine B (2) ^{1}H-^{13}C HMBC Spectrum (CD_3CN) Optimized for 8.3 Hz

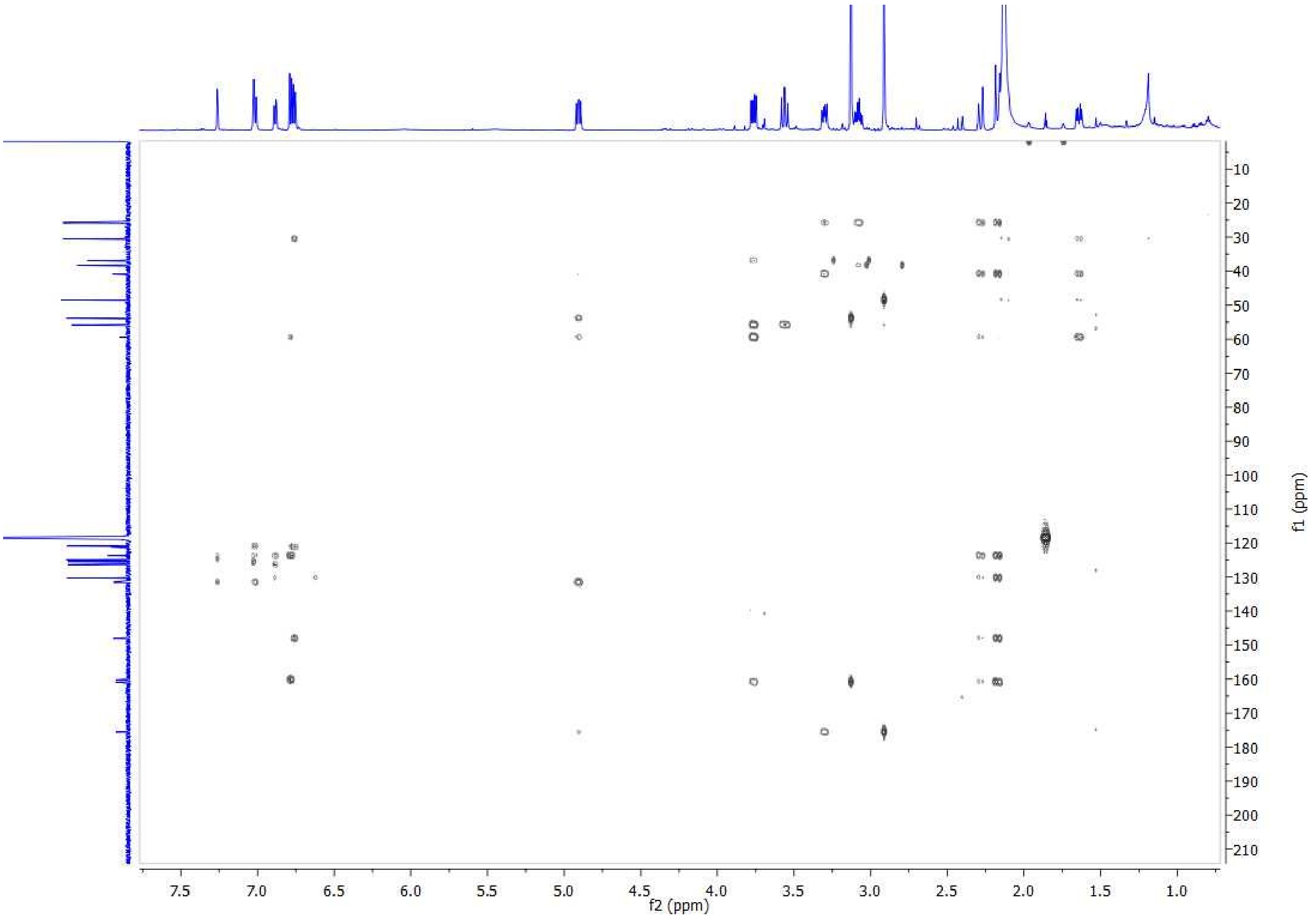

Caulamidine B (2) NOESY Spectrum (CD_3CN)

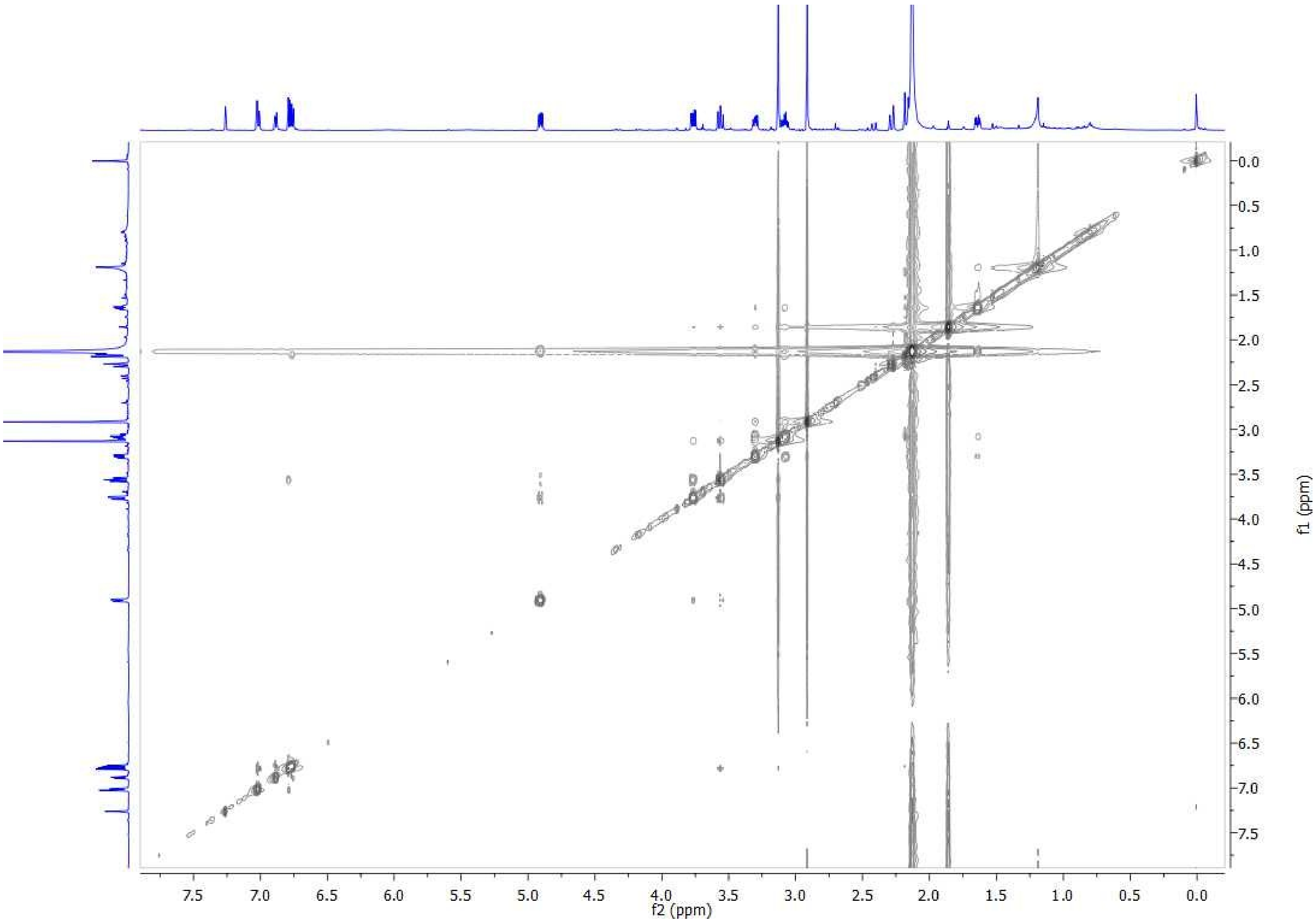

Caulamidine B (2) ROESY Spectrum (CD_3CN)

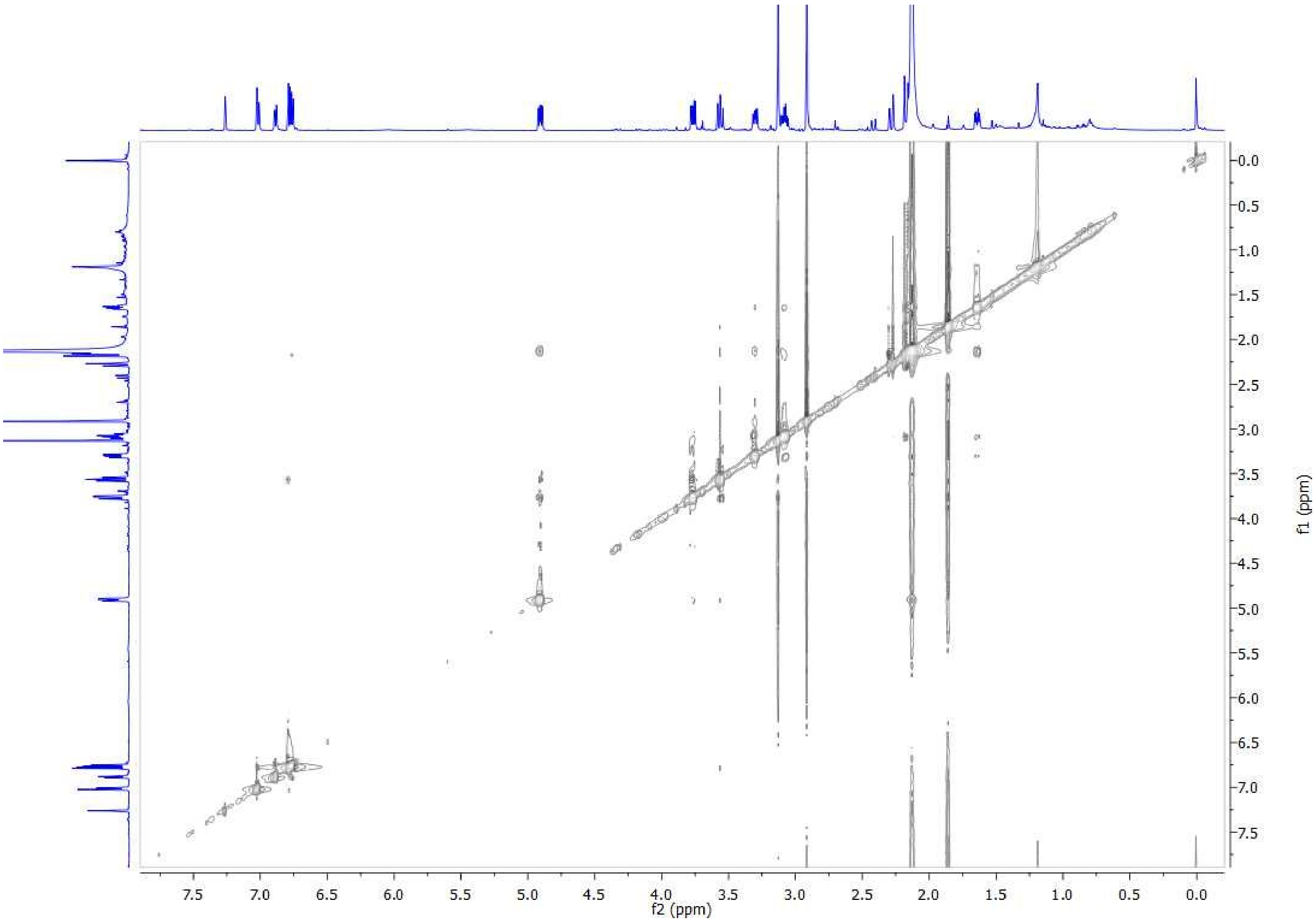

Caulamidine B (2) ^{15}N-^{1}H HMBC Spectrum (CD_3CN) Optimized for 8.0 Hz

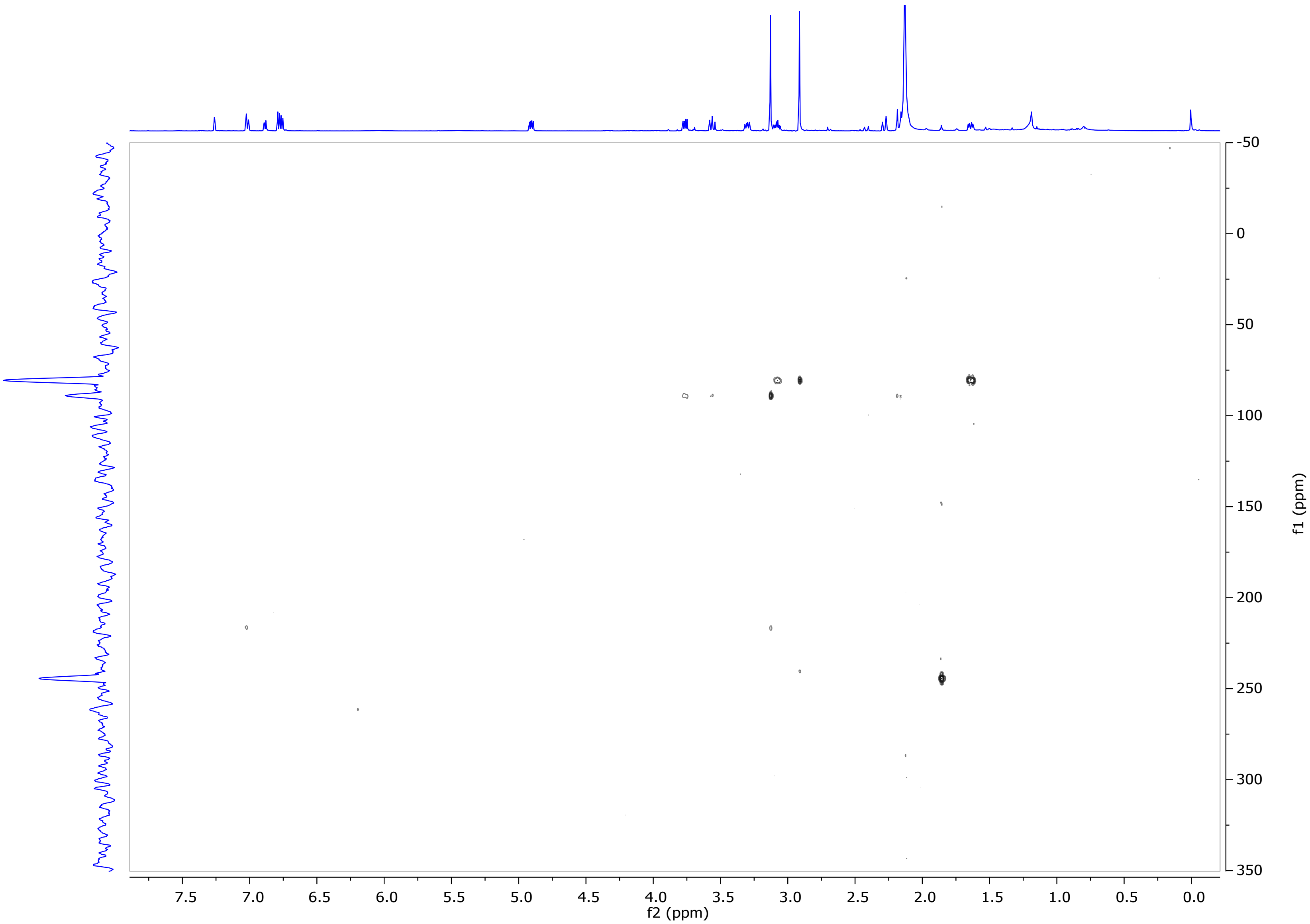

Caulamidine B (2) ^1H-^{13}C LR-HSQMBC Spectrum (CD_3CN) Optimized for 2Hz

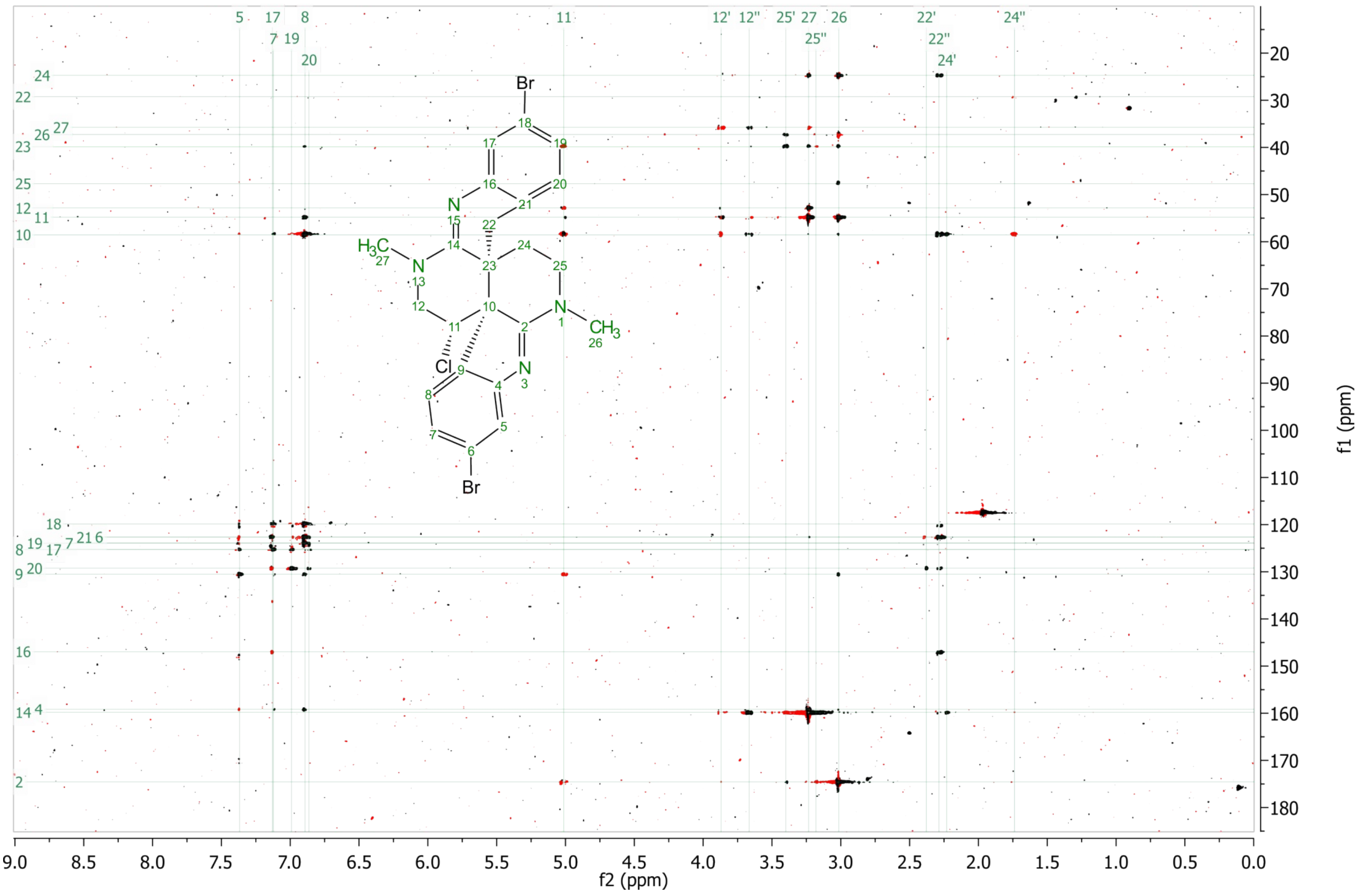

Caulamidine B (2) ^{1}H-^{13}C LR-HSQMBC Spectrum (CD_3CN) Optimized for 8Hz + 60 ms Mixing Time

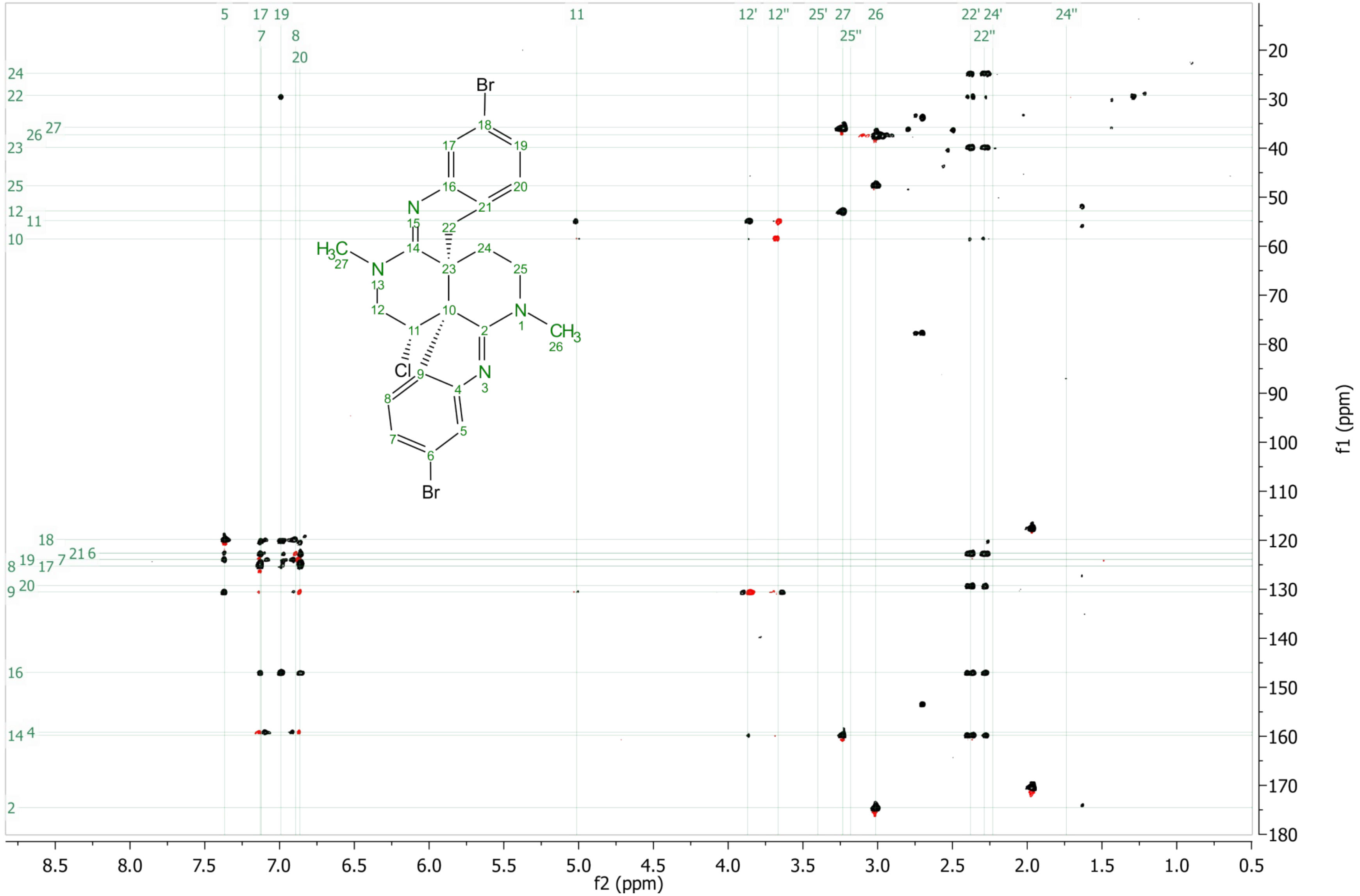

Caulamidine A (1) HD-*J*-HSQC Spectra for RDC Measurement Showing a Representative Region.
Spectra from weakly and strongly aligned samples are shown in red and blue respectively.

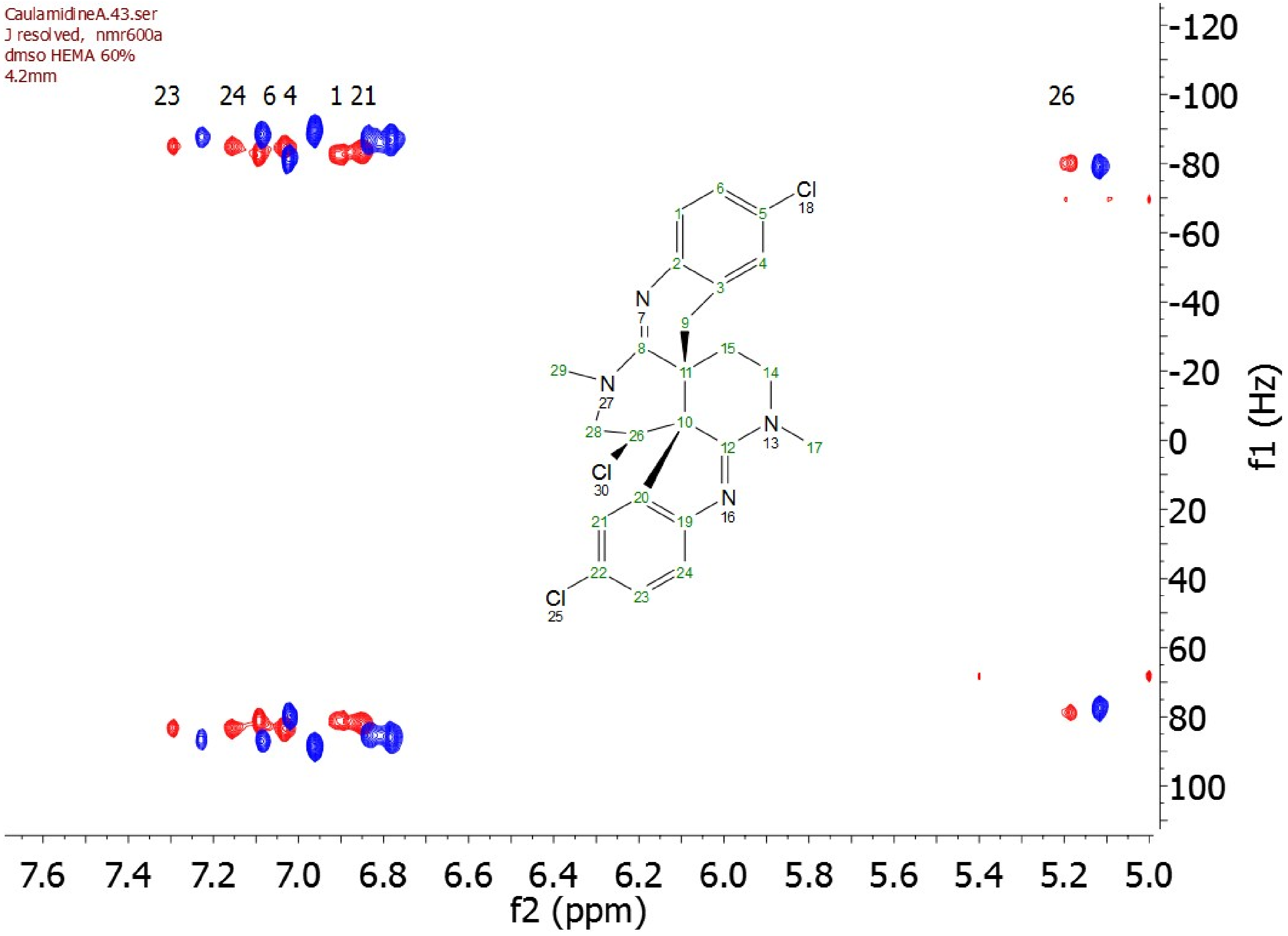

Caulamidine A (1) {^{1}H}-^{13}C Spectra for RCSA Measurement Showing a Representative Region.
Spectra from weakly and strongly aligned samples are shown in red and blue, respectively.

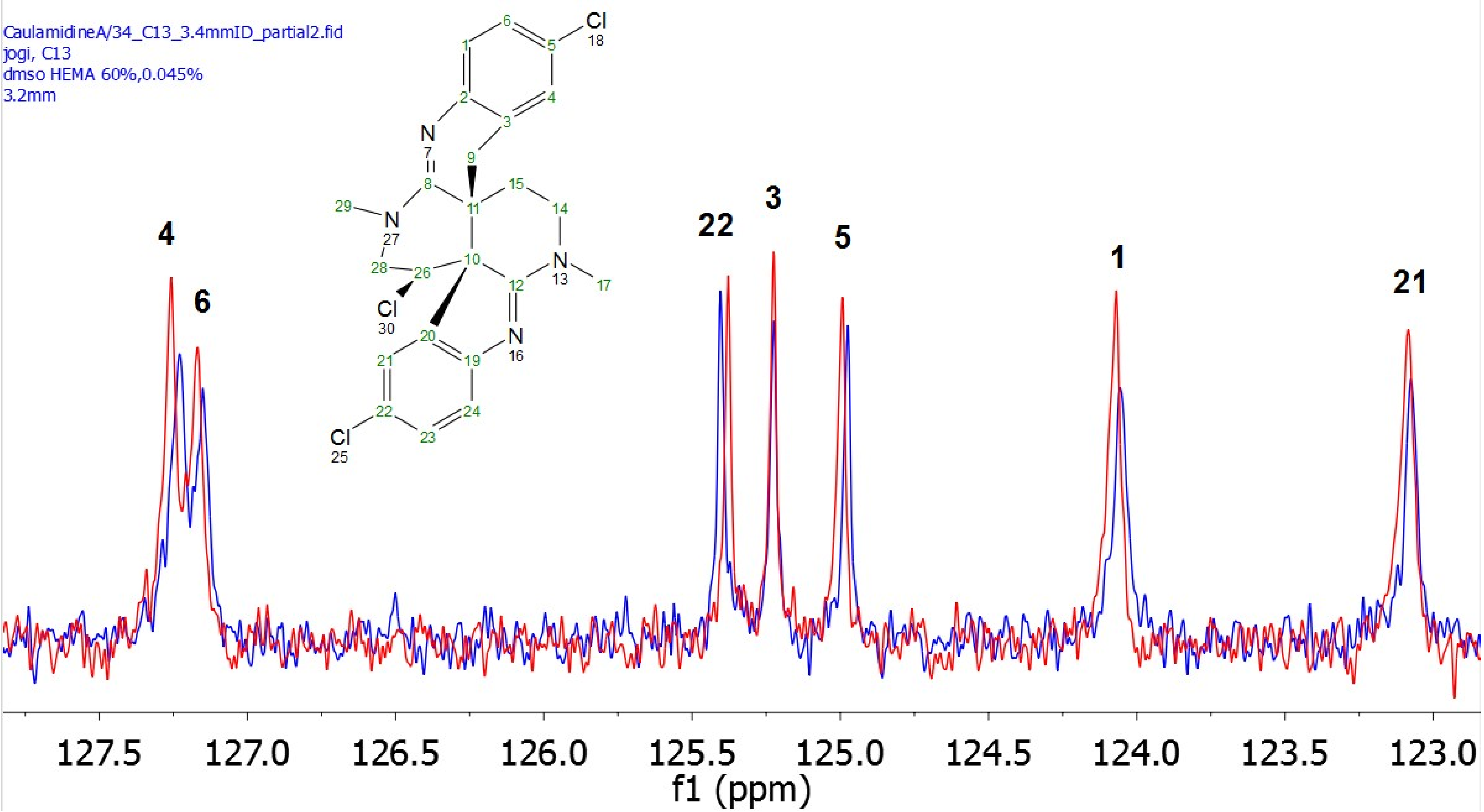

Structure Coordinate of Caulamidine A from DFT Geometry Optimization.

C	4.021	-1.026	-1.527
C	2.729	-0.675	-1.109
C	2.528	-0.270	0.227
C	3.604	-0.210	1.110
C	4.880	-0.556	0.667
C	5.099	-0.967	-0.648
N	1.679	-0.761	-2.029
C	0.556	-0.161	-1.783
C	1.110	0.020	0.638
C	-1.199	0.796	-0.128
C	0.313	0.675	-0.515
C	-1.377	1.864	0.964
N	-0.817	3.101	0.798
C	0.567	3.135	0.291
C	0.849	2.113	-0.814
N	-2.105	1.506	1.978
C	-1.108	4.099	1.823
Cl	6.236	-0.471	1.789
C	-2.429	0.151	1.763
C	-1.864	-0.372	0.580
C	-2.005	-1.713	0.255
C	-2.747	-2.522	1.127
C	-3.330	-2.012	2.286
C	-3.170	-0.662	2.615
Cl	-2.940	-4.231	0.742
C	-1.967	1.212	-1.410
N	-0.409	-0.151	-2.764
C	-1.811	0.170	-2.509
C	-0.158	-0.890	-3.999
Cl	-3.745	1.483	-1.142
H	-1.604	2.181	-1.747
H	-2.173	4.074	2.055
H	-0.837	5.086	1.441
H	-0.552	3.908	2.752
H	0.895	-0.803	-4.259

H	-0.780	-0.469	-4.794
H	-0.394	-1.957	-3.892
H	0.617	-0.921	0.908
H	1.074	0.651	1.533
H	0.761	4.141	-0.094
H	1.261	2.982	1.133
H	0.436	2.469	-1.761
H	1.933	2.059	-0.955
H	-2.243	0.565	-3.433
H	-2.387	-0.728	-2.245
H	4.162	-1.342	-2.556
H	3.453	0.098	2.140
H	6.097	-1.234	-0.976
H	-1.550	-2.151	-0.627
H	-3.899	-2.671	2.933
H	-3.605	-0.254	3.520

GIAO Chemical Shielding Tensors of caulamidine A.

1 C Isotropic = 73.3703 Anisotropy = 141.2073
XX= 61.7645 YX= 20.1256 ZX= 8.3978
XY= 22.6134 YY= 150.8281 ZY= -45.4062
XZ= 4.3523 YZ= -46.0083 ZZ= 7.5184
Eigenvalues: -7.9826 60.5851 167.5086
2 C Isotropic = 56.1240 Anisotropy = 151.0068
XX= 2.2535 YX= 21.9017 ZX= -31.7615
XY= 5.5815 YY= 147.1450 ZY= -30.3660
XZ= -38.4038 YZ= -29.5607 ZZ= 18.9735
Eigenvalues: -25.8332 37.4100 156.7952
3 C Isotropic = 73.9681 Anisotropy = 155.5631
XX= 11.4624 YX= 28.4309 ZX= 14.6050
XY= 30.6791 YY= 164.5843 ZY= -31.0167
XZ= 7.3042 YZ= -36.9867 ZZ= 45.8575
Eigenvalues: -1.0643 45.2916 177.6768
4 C Isotropic = 72.1485 Anisotropy = 124.9639
XX= 71.4064 YX= 14.7081 ZX= 6.0047
XY= 8.8009 YY= 141.5472 ZY= -44.4196
XZ= 12.3490 YZ= -44.2133 ZZ= 3.4921
Eigenvalues: -11.2938 72.2816 155.4578
5 C Isotropic = 62.7872 Anisotropy = 96.2986
XX= 23.5580 YX= 4.1230 ZX= -47.1358
XY= 5.5475 YY= 122.6791 ZY= -11.9696
XZ= -47.2617 YZ= -15.7686 ZZ= 42.1245
Eigenvalues: -15.4510 76.8263 126.9863
6 C Isotropic = 71.0978 Anisotropy = 141.3478
XX= 1.3397 YX= 33.9428 ZX= 19.4546
XY= 34.0807 YY= 150.8592 ZY= -30.3426
XZ= 16.5239 YZ= -32.1980 ZZ= 61.0945
Eigenvalues: -14.2618 62.2255 165.3297
7 N Isotropic = 30.5974 Anisotropy = 329.5536
XX= -90.3982 YX= 72.9496 ZX= -107.6496
XY= 101.8368 YY= 176.1129 ZY= -78.8872
XZ= -107.6878 YZ= -74.5075 ZZ= 6.0775

Eigenvalues: -163.6262 5.1186 250.2998
8 C Isotropic = 42.1054 Anisotropy = 122.4684
XX= 36.7657 YX= 10.4281 ZX= -55.4613
XY= 38.1858 YY= 90.2246 ZY= -33.8850
XZ= -19.9424 YZ= -52.5531 ZZ= -0.6742
Eigenvalues: -29.8249 32.3900 123.7510
9 C Isotropic = 163.9295 Anisotropy = 30.7497
XX= 183.1890 YX= -9.7276 ZX= -2.1628
XY= -2.3796 YY= 149.9786 ZY= -7.5451
XZ= 4.2144 YZ= -4.5715 ZZ= 158.6209
Eigenvalues: 146.1904 161.1687 184.4293
10 C Isotropic = 133.6350 Anisotropy = 8.4992
XX= 132.1560 YX= 0.4894 ZX= 2.3603
XY= 2.4525 YY= 138.5751 ZY= -3.1071
XZ= -0.3945 YZ= -1.1990 ZZ= 130.1738
Eigenvalues: 129.0778 132.5260 139.3011
11 C Isotropic = 149.8353 Anisotropy = 10.5135
XX= 150.8640 YX= 6.5660 ZX= -1.5064
XY= 0.6875 YY= 154.3546 ZY= 5.0037
XZ= -4.6389 YZ= 2.2930 ZZ= 144.2872
Eigenvalues: 141.3115 151.3500 156.8443
12 C Isotropic = 25.6525 Anisotropy = 119.8213
XX= 61.0411 YX= -45.6530 ZX= 34.0583
XY= -22.0725 YY= -24.2707 ZY= -58.7191
XZ= 29.5146 YZ= -26.0970 ZZ= 40.1869
Eigenvalues: -48.1664 19.5904 105.5333
13 N Isotropic = 178.6561 Anisotropy = 57.0590
XX= 179.2225 YX= 16.6399 ZX= 34.0686
XY= -3.7729 YY= 206.5919 ZY= 8.9946
XZ= 54.6386 YZ= -1.7402 ZZ= 150.1540
Eigenvalues: 118.0060 201.2669 216.6954
14 C Isotropic = 146.4623 Anisotropy = 52.3242
XX= 172.5768 YX= 3.0525 ZX= -21.9418
XY= 1.9261 YY= 131.6762 ZY= 9.6545
XZ= -18.1439 YZ= 10.5424 ZZ= 135.1341
Eigenvalues: 118.4499 139.5919 181.3452
15 C Isotropic = 168.0703 Anisotropy = 18.5335

XX= 154.2974 YX= 10.3105 ZX= -8.0709
XY= -4.0269 YY= 177.7419 ZY= 2.0173
XZ= 1.3805 YZ= 7.2840 ZZ= 172.1715
Eigenvalues: 153.0483 170.7366 180.4259
16 N Isotropic = 1.8926 Anisotropy = 324.9541
XX= 109.3593 YX= -142.5246 ZX= 88.8207
XY= -130.9505 YY= -145.5634 ZY= -10.5896
XZ= 118.5731 YZ= 24.8457 ZZ= 41.8819
Eigenvalues: -214.8154 1.9646 218.5287
17 C Isotropic = 157.1465 Anisotropy = 53.7879
XX= 142.9931 YX= -8.8105 ZX= -6.3505
XY= -14.4810 YY= 166.4434 ZY= 25.7413
XZ= -9.1196 YZ= 23.9804 ZZ= 162.0031
Eigenvalues: 137.1422 141.2923 193.0051
18 Cl Isotropic = 745.6722 Anisotropy = 445.7861
XX= 857.7816 YX= 20.1521 ZX= 222.5571
XY= 17.9074 YY= 606.9492 ZY= 11.0637
XZ= 222.9181 YZ= 9.2948 ZZ= 772.2857
Eigenvalues: 587.2543 606.8993 1042.8629
19 C Isotropic = 42.6006 Anisotropy = 133.3178
XX= 104.8531 YX= -19.7264 ZX= 32.8428
XY= -32.9676 YY= -27.9125 ZY= -24.0419
XZ= 38.3681 YZ= -38.1621 ZZ= 50.8613
Eigenvalues: -40.0820 36.4047 131.4792
20 C Isotropic = 64.8497 Anisotropy = 140.7011
XX= 109.2979 YX= -33.8811 ZX= 70.7649
XY= -33.1744 YY= 27.3681 ZY= 4.6508
XZ= 60.6524 YZ= 8.7023 ZZ= 57.8830
Eigenvalues: -6.5843 42.4829 158.6504
21 C Isotropic = 74.9504 Anisotropy = 134.3850
XX= 124.2024 YX= 0.8888 ZX= 63.4456
XY= -5.8062 YY= 55.9753 ZY= -31.4389
XZ= 68.0744 YZ= -34.6500 ZZ= 44.6735
Eigenvalues: -5.2541 65.5649 164.5404
22 C Isotropic = 63.0850 Anisotropy = 95.0787
XX= 110.5856 YX= -21.6542 ZX= 17.3036
XY= -19.1233 YY= -7.8558 ZY= -23.7352

XZ= 18.1375 YZ= -24.2073 ZZ= 86.5251
Eigenvalues: -15.6206 78.4047 126.4708
23 C Isotropic = 69.0081 Anisotropy = 145.8769
XX= 111.3271 YX= -44.5049 ZX= 70.7496
XY= -41.9390 YY= 36.3844 ZY= 12.9909
XZ= 69.3082 YZ= 14.8894 ZZ= 59.3127
Eigenvalues: -16.3294 57.0944 166.2593
24 C Isotropic = 78.7263 Anisotropy = 147.2814
XX= 131.5354 YX= -12.2772 ZX= 69.1256
XY= -12.2632 YY= 56.5800 ZY= -33.2231
XZ= 70.0949 YZ= -30.7667 ZZ= 48.0635
Eigenvalues: -0.3500 59.6151 176.9139
25 Cl Isotropic = 744.3695 Anisotropy = 449.2518
XX= 606.3369 YX= 44.4931 ZX= 7.9014
XY= 46.1914 YY= 1017.6208 ZY= 97.2326
XZ= 16.9450 YZ= 93.6977 ZZ= 609.1508
Eigenvalues: 587.5620 601.6758 1043.8707
26 C Isotropic = 131.9597 Anisotropy = 37.9082
XX= 155.8470 YX= -3.7316 ZX= 0.6133
XY= 0.2230 YY= 103.6539 ZY= 1.3887
XZ= -10.9790 YZ= 2.7745 ZZ= 136.3782
Eigenvalues: 103.4827 135.1646 157.2318
27 N Isotropic = 168.4667 Anisotropy = 63.9112
XX= 146.8593 YX= 31.1782 ZX= 41.2037
XY= 27.8584 YY= 165.3508 ZY= -15.8643
XZ= 24.7464 YZ= -3.3920 ZZ= 193.1901
Eigenvalues: 112.0381 182.2880 211.0742
28 C Isotropic = 142.1216 Anisotropy = 55.2026
XX= 165.5857 YX= -16.7552 ZX= -11.1831
XY= -17.0915 YY= 126.2872 ZY= 10.2945
XZ= -17.2476 YZ= 14.0455 ZZ= 134.4918
Eigenvalues: 116.6099 130.8314 178.9233
29 C Isotropic = 158.5209 Anisotropy = 51.2351
XX= 139.0610 YX= -3.0297 ZX= -7.1055
XY= -10.0630 YY= 153.8083 ZY= 16.0405
XZ= -10.2752 YZ= 17.9747 ZZ= 182.6934
Eigenvalues: 136.4199 146.4652 192.6777

30 Cl Isotropic = 825.2891 Anisotropy = 398.7389
XX= 1067.8549 YX= -88.1460 ZX= -36.1264
XY= -66.6517 YY= 691.7209 ZY= 24.1388
XZ= -61.5745 YZ= 43.7720 ZZ= 716.2915
Eigenvalues: 663.8824 720.8699 1091.1150
31 H Isotropic = 26.9536 Anisotropy = 5.4980
XX= 29.8788 YX= 1.5543 ZX= 0.9342
XY= 1.6556 YY= 26.9053 ZY= -1.4178
XZ= -0.3150 YZ= -2.3721 ZZ= 24.0767
Eigenvalues: 22.9752 27.2667 30.6189
32 H Isotropic = 28.0650 Anisotropy = 10.4456
XX= 30.1133 YX= -2.0861 ZX= -3.6483
XY= -3.1952 YY= 28.3072 ZY= 4.2407
XZ= -4.1561 YZ= 2.9942 ZZ= 25.7745
Eigenvalues: 22.6684 26.4978 35.0287
33 H Isotropic = 29.5112 Anisotropy = 10.8451
XX= 25.0117 YX= -0.6675 ZX= -1.1310
XY= -0.0672 YY= 36.4224 ZY= 1.0397
XZ= -0.8780 YZ= 2.3251 ZZ= 27.0994
Eigenvalues: 24.5992 27.1930 36.7413
34 H Isotropic = 29.0133 Anisotropy = 10.5733
XX= 25.3539 YX= 0.2474 ZX= 1.7516
XY= -0.1575 YY= 27.5358 ZY= 4.0078
XZ= 2.4191 YZ= 3.1407 ZZ= 34.1504
Eigenvalues: 24.5705 26.4073 36.0622
35 H Isotropic = 27.2668 Anisotropy = 9.9417
XX= 30.0622 YX= -0.6155 ZX= -2.5279
XY= -1.5669 YY= 21.4167 ZY= 2.0695
XZ= -3.9125 YZ= 2.5936 ZZ= 30.3215
Eigenvalues: 20.8327 27.0731 33.8946
36 H Isotropic = 29.6406 Anisotropy = 10.3484
XX= 26.1726 YX= -1.4245 ZX= 0.7071
XY= -1.5115 YY= 26.4451 ZY= 0.7749
XZ= 2.3895 YZ= 0.0732 ZZ= 36.3041
Eigenvalues: 24.6564 27.7259 36.5395
37 H Isotropic = 28.8680 Anisotropy = 10.8856
XX= 24.2393 YX= 0.6753 ZX= -0.6464

XY= 1.2875 YY= 33.8310 ZY= 3.7728
XZ= -0.7149 YZ= 4.5161 ZZ= 28.5335
Eigenvalues: 23.7602 26.7186 36.1250
38 H Isotropic = 29.0276 Anisotropy = 10.6224
XX= 35.3426 YX= 1.4134 ZX= -2.1505
XY= 2.6187 YY= 28.0268 ZY= -4.2969
XZ= 0.6384 YZ= -3.1095 ZZ= 23.7134
Eigenvalues: 21.5754 29.3982 36.1092
39 H Isotropic = 29.7828 Anisotropy = 5.9403
XX= 31.8159 YX= -1.7767 ZX= 0.6611
XY= -3.0972 YY= 26.0882 ZY= 2.2371
XZ= 3.5257 YZ= 2.3867 ZZ= 31.4443
Eigenvalues: 24.0377 31.5678 33.7430
40 H Isotropic = 28.8647 Anisotropy = 9.0542
XX= 27.0462 YX= 4.0491 ZX= -2.9172
XY= 1.2815 YY= 33.8819 ZY= 0.1657
XZ= -2.2185 YZ= -0.3843 ZZ= 25.6660
Eigenvalues: 23.4511 28.2422 34.9009
41 H Isotropic = 28.7092 Anisotropy = 7.4608
XX= 32.0266 YX= 0.6688 ZX= 3.3941
XY= -1.2609 YY= 27.2452 ZY= 4.0218
XZ= 2.6607 YZ= 2.8103 ZZ= 26.8558
Eigenvalues: 22.9575 29.4871 33.6831
42 H Isotropic = 29.7416 Anisotropy = 6.1177
XX= 26.1179 YX= 2.4225 ZX= 0.0154
XY= -0.1327 YY= 30.5919 ZY= -2.0349
XZ= 2.1269 YZ= -2.0438 ZZ= 32.5151
Eigenvalues: 25.4854 29.9195 33.8201
43 H Isotropic = 29.9960 Anisotropy = 7.2667
XX= 33.9750 YX= 0.3413 ZX= -2.7971
XY= -2.6364 YY= 31.1269 ZY= 0.3502
XZ= -1.8709 YZ= -0.8745 ZZ= 24.8860
Eigenvalues: 24.2809 30.8665 34.8404
44 H Isotropic = 28.5233 Anisotropy = 7.6006
XX= 28.3106 YX= -3.4353 ZX= 3.0521
XY= -2.8245 YY= 24.3881 ZY= -0.0126
XZ= 0.6269 YZ= 0.6758 ZZ= 32.8711

Eigenvalues: 22.5173 29.4622 33.5904
45 H Isotropic = 28.0484 Anisotropy = 4.0128
XX= 28.0204 YX= 2.9271 ZX= 0.4015
XY= 2.3852 YY= 28.1132 ZY= 0.7291
XZ= -2.2452 YZ= 1.0154 ZZ= 28.0116
Eigenvalues: 24.8938 28.5278 30.7236
46 H Isotropic = 24.5374 Anisotropy = 6.5512
XX= 28.5921 YX= -1.7171 ZX= 0.3344
XY= -1.3646 YY= 21.0710 ZY= 1.2504
XZ= 0.6082 YZ= 1.3616 ZZ= 23.9492
Eigenvalues: 20.2567 24.4507 28.9049
47 H Isotropic = 24.9006 Anisotropy = 11.2089
XX= 31.6052 YX= -1.8748 ZX= 1.7725
XY= -2.0262 YY= 20.1046 ZY= 0.6602
XZ= 2.5401 YZ= 0.4247 ZZ= 22.9922
Eigenvalues: 19.5315 22.7972 32.3733
48 H Isotropic = 24.4840 Anisotropy = 5.9518
XX= 25.0908 YX= -0.6856 ZX= 1.2924
XY= -0.8746 YY= 20.7083 ZY= 2.1289
XZ= 0.7234 YZ= 2.2150 ZZ= 27.6530
Eigenvalues: 19.8796 25.1206 28.4519
49 H Isotropic = 24.7006 Anisotropy = 12.1858
XX= 21.6119 YX= 3.1805 ZX= -2.7905
XY= 5.1495 YY= 29.8404 ZY= -2.7189
XZ= -2.8492 YZ= -2.5623 ZZ= 22.6494
Eigenvalues: 18.9487 22.3286 32.8245
50 H Isotropic = 24.3207 Anisotropy = 6.0724
XX= 21.5565 YX= 0.4059 ZX= -2.9132
XY= 0.4218 YY= 25.8285 ZY= 2.2790
XZ= -3.0551 YZ= 1.9485 ZZ= 25.5772
Eigenvalues: 19.6509 24.9423 28.3690
51 H Isotropic = 24.2814 Anisotropy = 4.8745
XX= 22.2474 YX= 1.7701 ZX= -2.7518
XY= 1.9463 YY= 25.9261 ZY= -0.3684
XZ= -2.6241 YZ= -0.1247 ZZ= 24.6706
Eigenvalues: 20.1435 25.1695 27.5310

Table SI 8. NMR Spectroscopic Data for Caulamidine A (1) TFA Salt in CD_3CN

Pos.	δ_C, type[a]	δ_H (*J* in Hz)	NOE[b]	HMBC
2	169.4, C			
4	148.8, C			
5	117.1, CH	7.39, d (8.4)	H6	C4, 7, 9
6	132.0, CH	7.47, dd (8.4, 1.8)	H5	C4, 7, 8
7	129.7, C			
8	125.5, C	7.23, d (1.8)	H12, 22, 27	C4, 6, 7, 10
9	128.3, C			
10	57.8, C			
11	53.0, CH	5.06, dd (10.8, 6.4)	H12, 24, 27	C2, 9, 10, 12, 23
12 b	54.6, CH_2	3.94, dd (14.3, 11.0)	H8, 11, 27	C11, 14
a		4.15, dd (14.3, 6.6)	H8, 27	C10, 11, 14, 27
14	160.4, C			
16	134.0, C			
17	121.0, CH	7.55, d (8.6)	H18	C16, 19, 21
18	129.3, CH	7.33, dd (8.4, 2.2)	H17	C16, 19, 20
19	131.4, C			
20	129.5, CH	7.15, br d (2.2)	H22, 25	C16, 18, 19, 22
21	124.5, C			
22 b	30.0, CH_2	2.61, d (16.1)	H20, 24, 25	C10, 14, 16, 20, 21, 23, 24
a		2.78, d (15.7)	H8, 20	C10, 14, 16, 20, 21, 23, 24
23	41.2, C			
24 a	25.2, CH_2	2.38, dt (15.1, 8.1)	H11, 25	C14, 22, 23, 25
b		2.01, ddd (14.7, 7.0, 2.2)	H22, 25	C10, 22, 23, 25
25 a	48.1, CH_2	3.67, br dd (13.8, 7.3)	H24, 26	C2, 23, 24
b		3.42, m	H22, 24, 26	C2, 23, 24
26	39.9, CH_3	3.27 (s)	H25	C2, 25
27	40.6, CH_3	3.53 (s)	H8, 11, 12	C12, 14

[a]multiplicity from multiplicity edited HSQC data. [b]NOESY and ROESY interactions, geminal NOE's omitted

Caulamidine A (1) TFA Salt ^{1}H NMR Spectrum (500 MHz, CD_3CN)

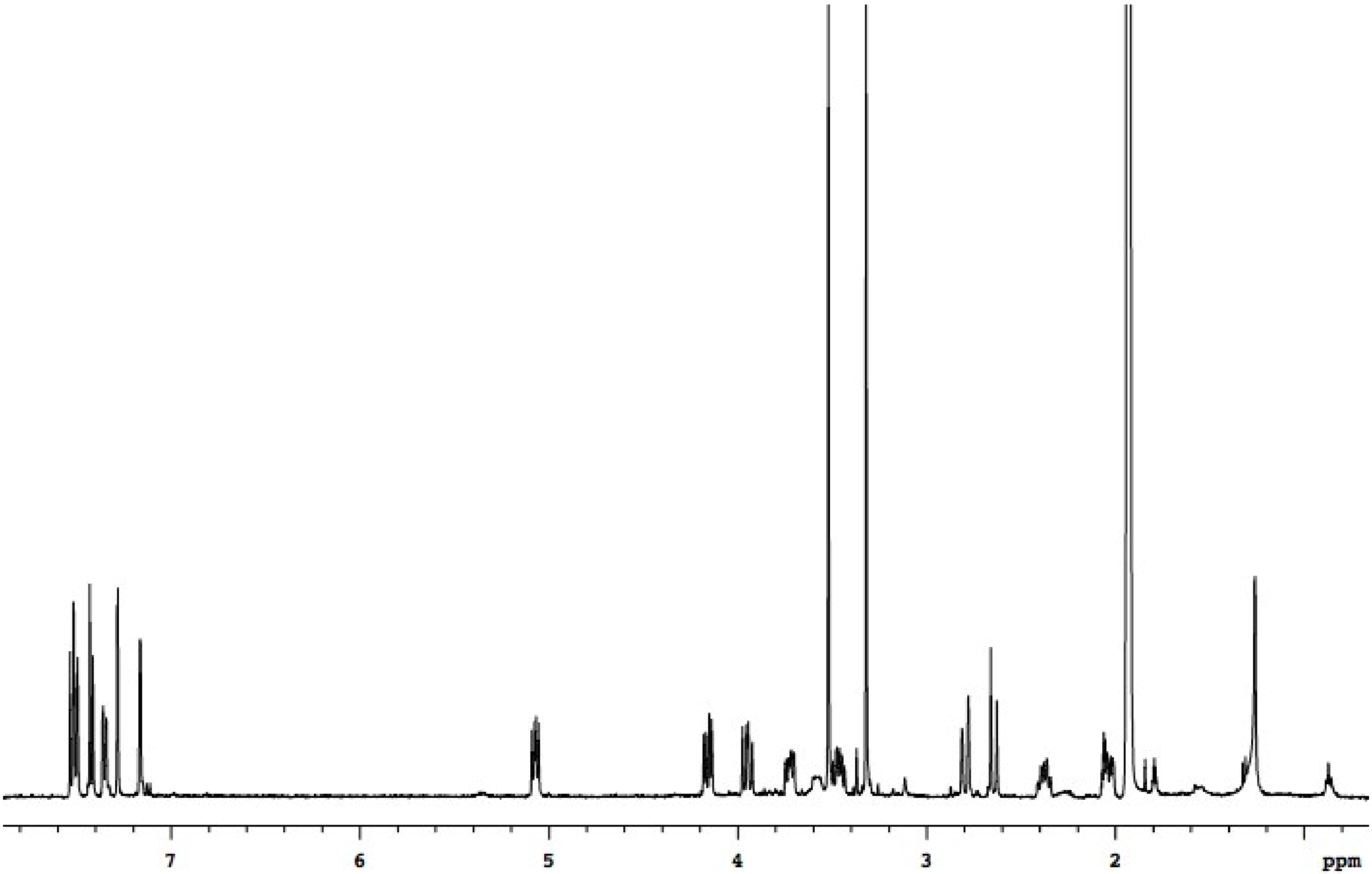

Caulamidine A (1) TFA Salt ^{13}C NMR Spectrum (125 MHz, CD_3CN)

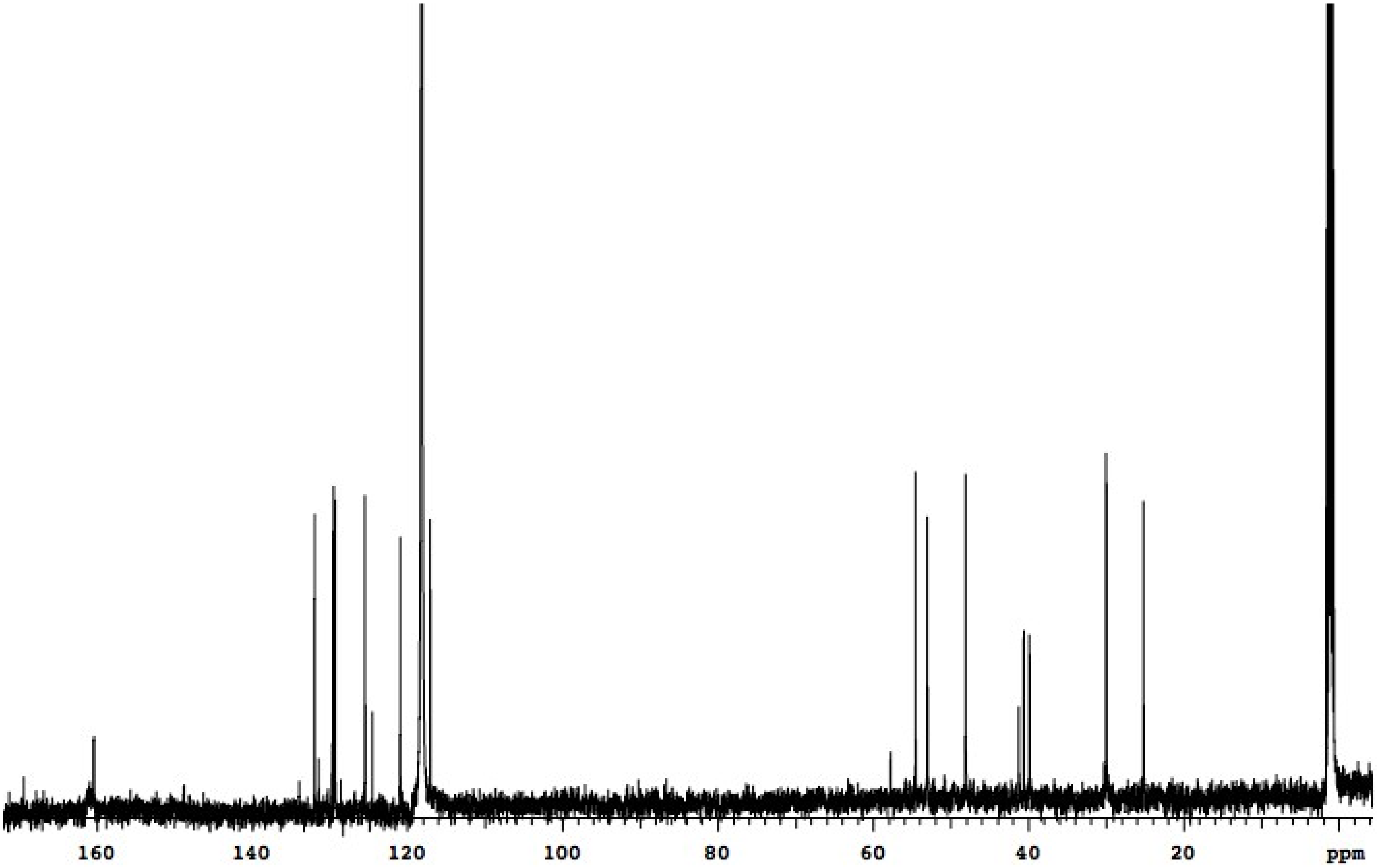

Caulamidine A (1) TFA Salt COSY Spectrum (CD_3CN)

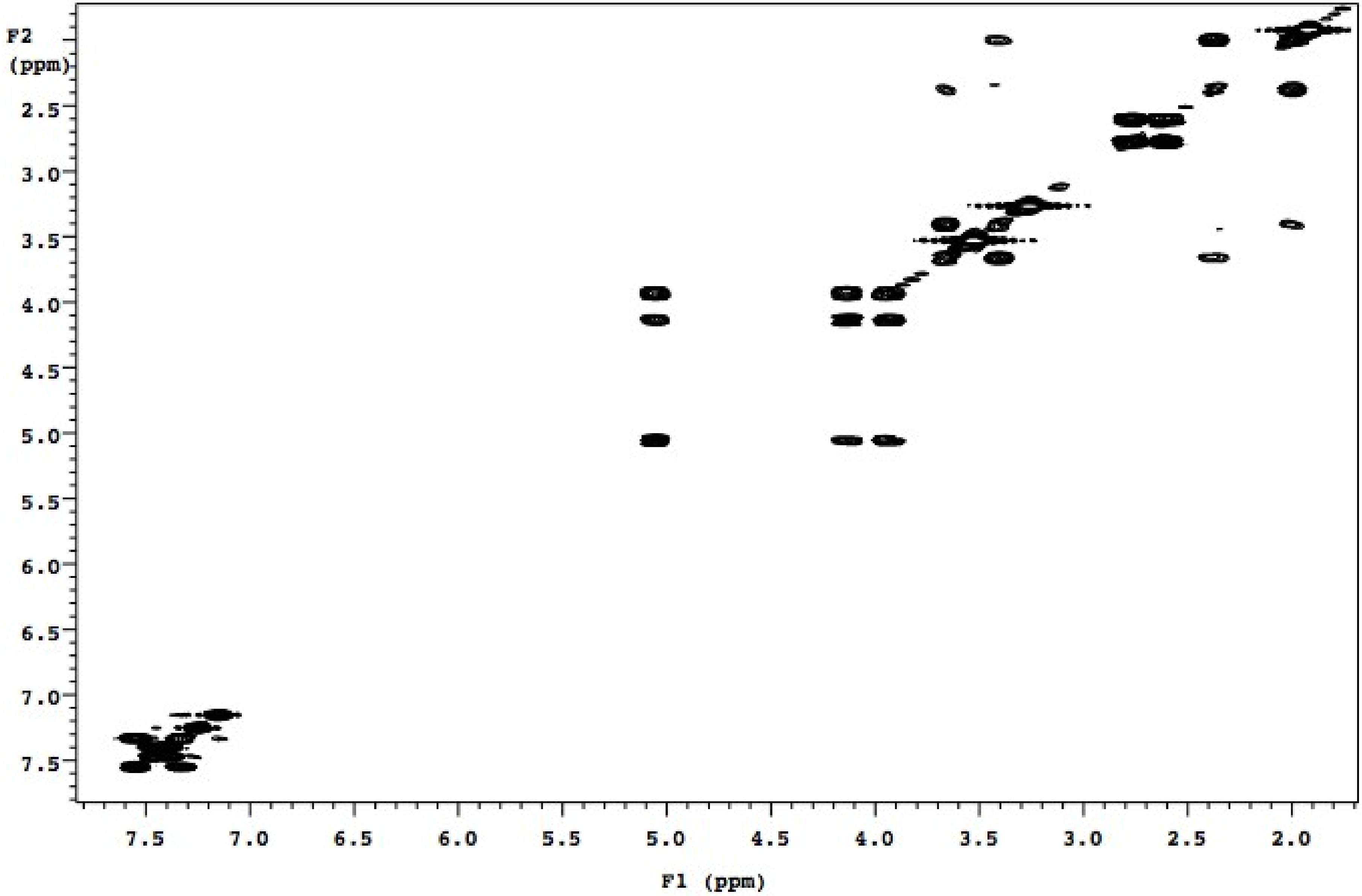

Caulamidine A (1) TFA Salt HSQC Spectrum (CD_3CN)

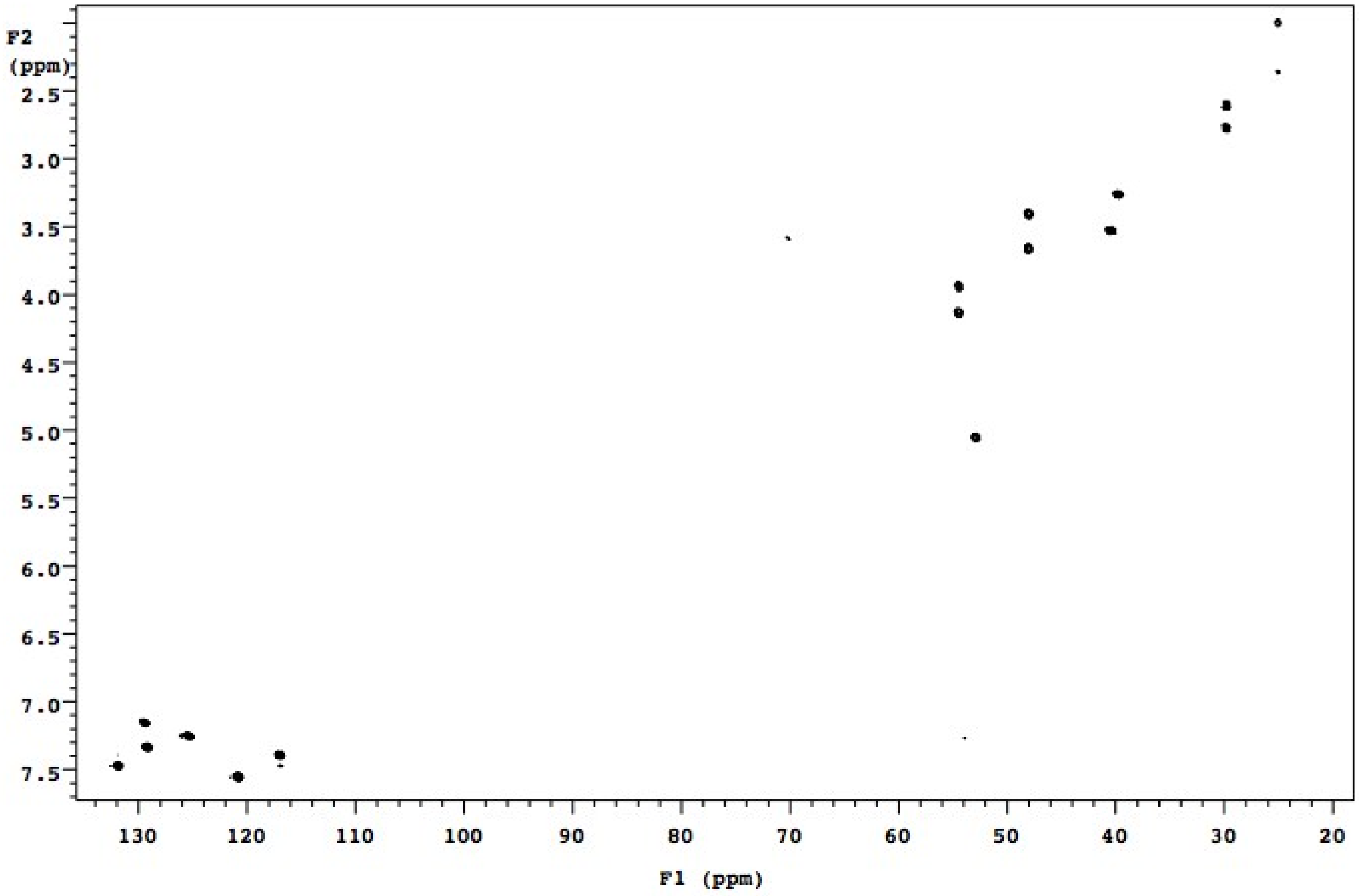

Caulamidine A (1) TFA Salt ^{1}H-^{13}C HMBC Spectrum (CD_3CN) Optimized for 8.3 Hz

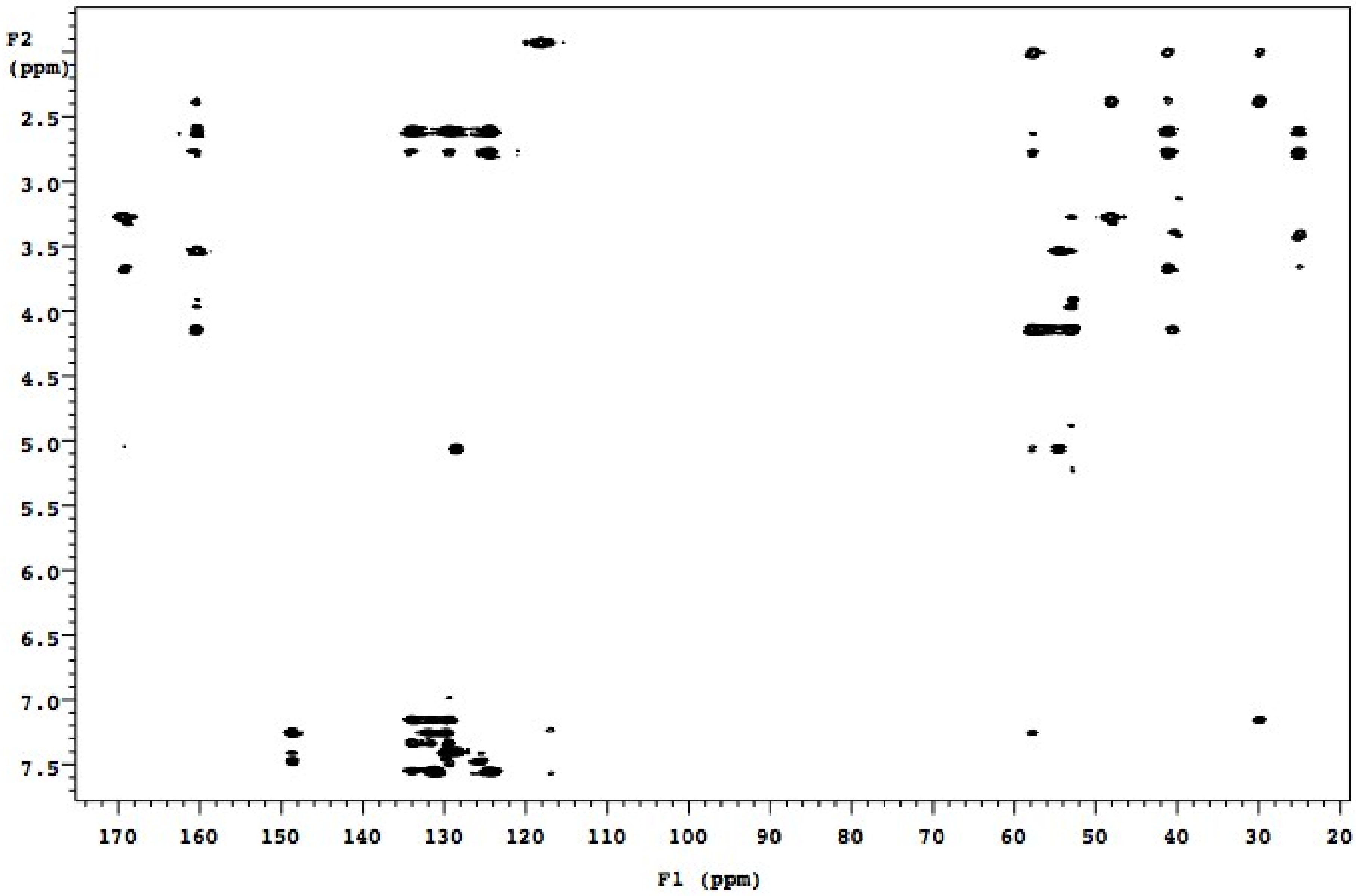

Caulamidine A (1) TFA Salt ROESY Spectrum (CD_3CN)

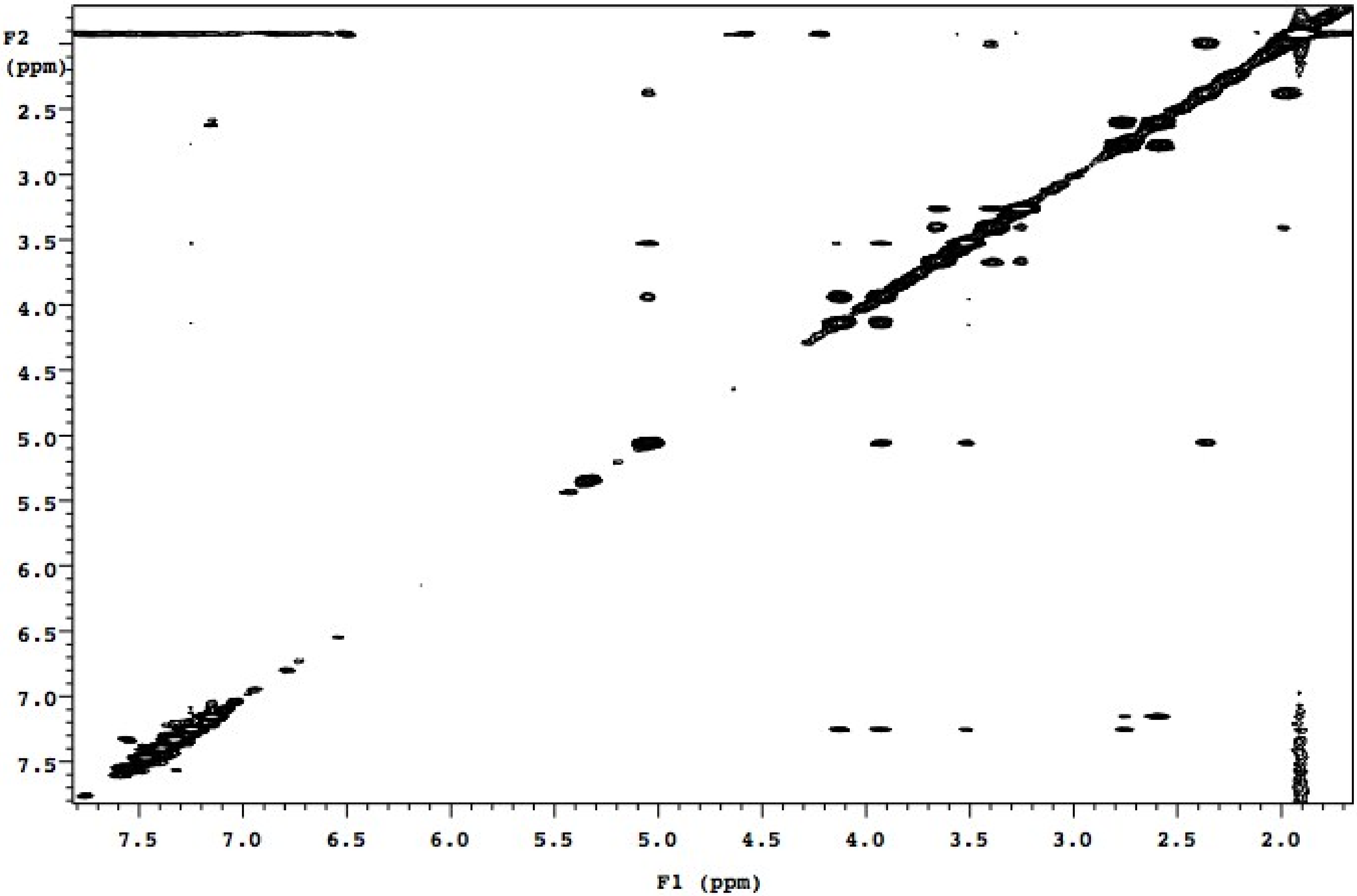

Selective (H-8) 1D NOESY spectrum of caulamidine A (1) TFA salt in CD_3CN

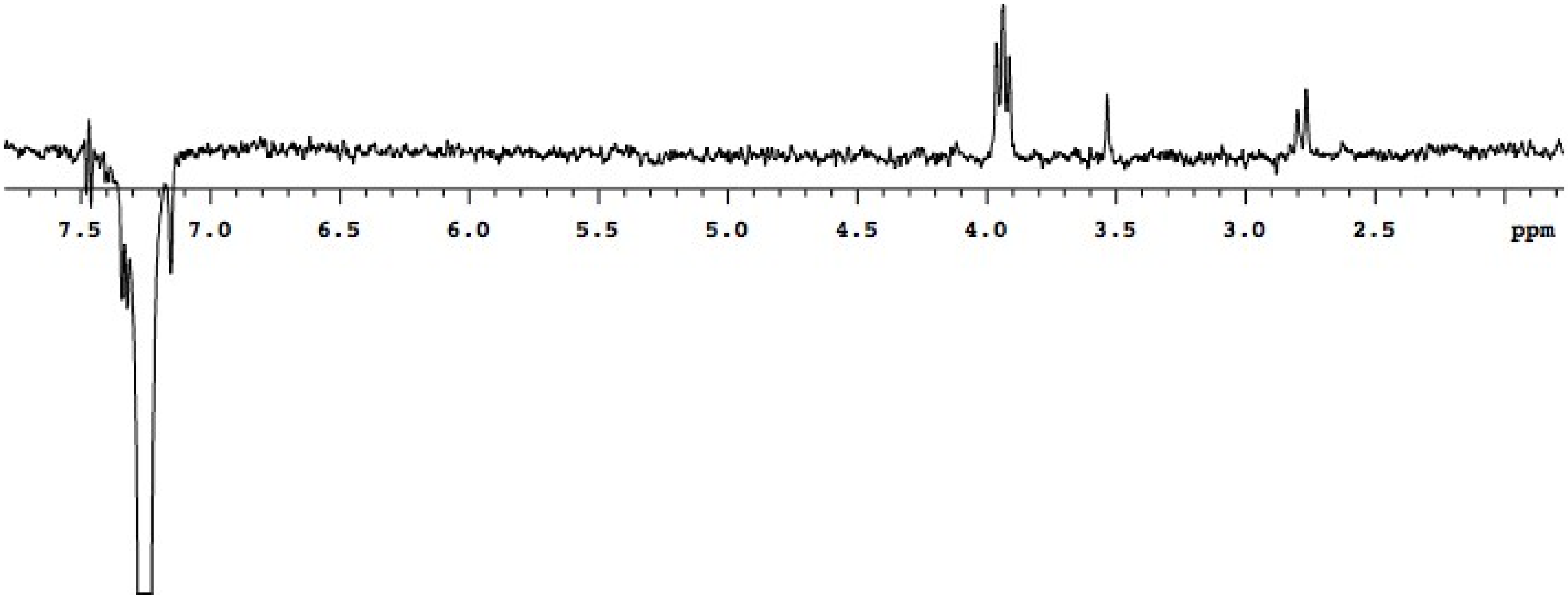

Selective (H-22a) 1D NOESY spectrum of caulamidine in CD_3CN.

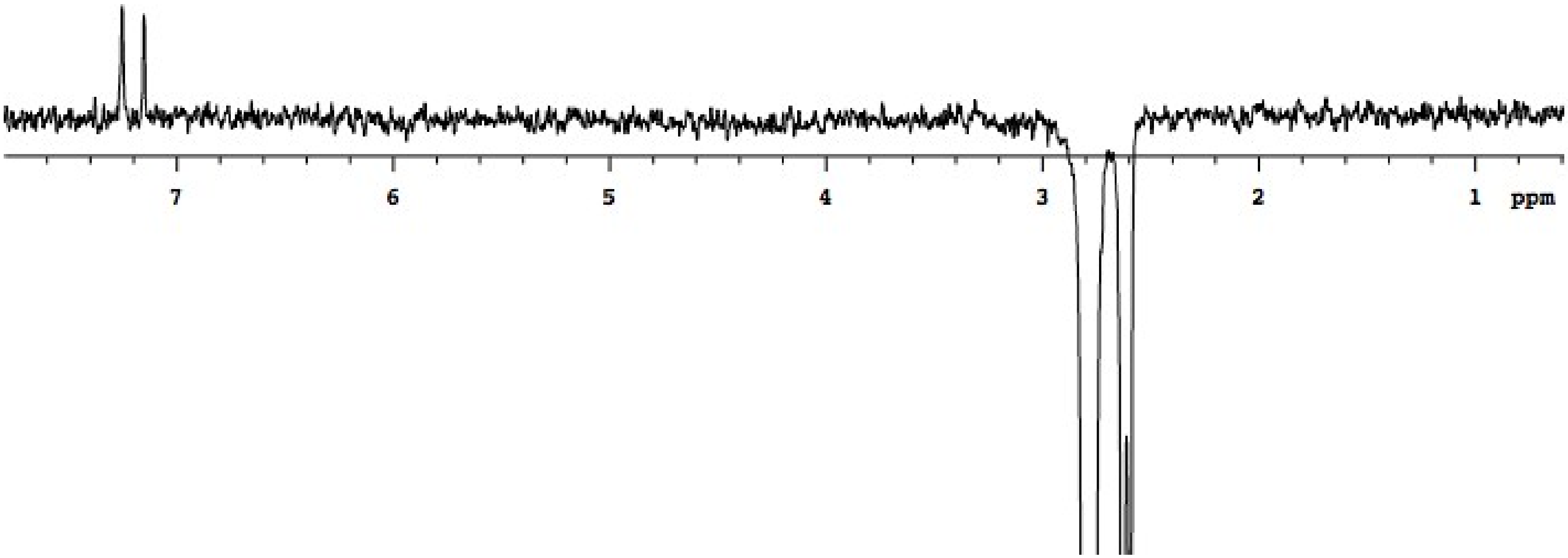

Selective (H24a) 1D NOESY spectrum of caulamidine in CD_3CN.

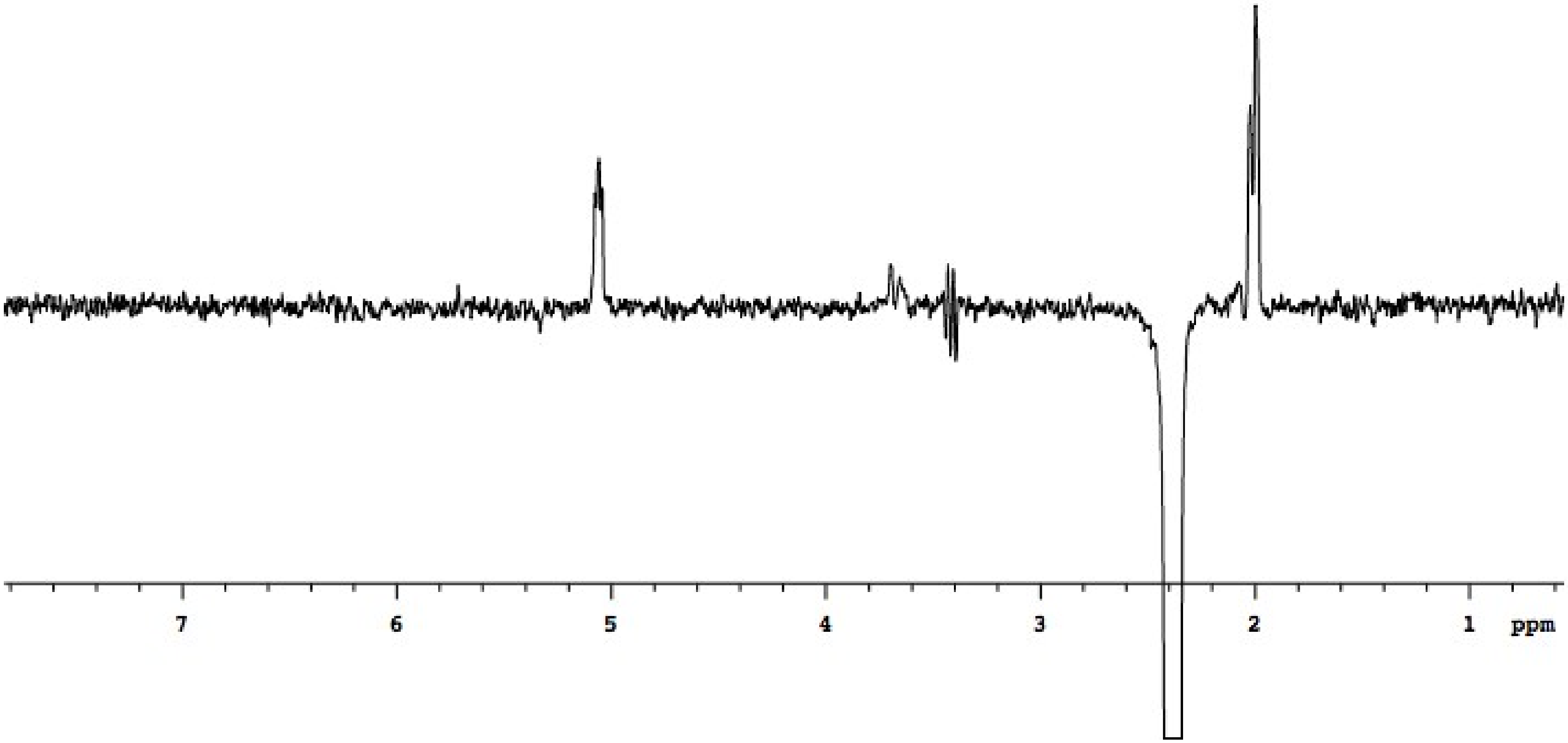

Selective (H11) 1D NOESY spectrum of caulamidine in CD_3CN.

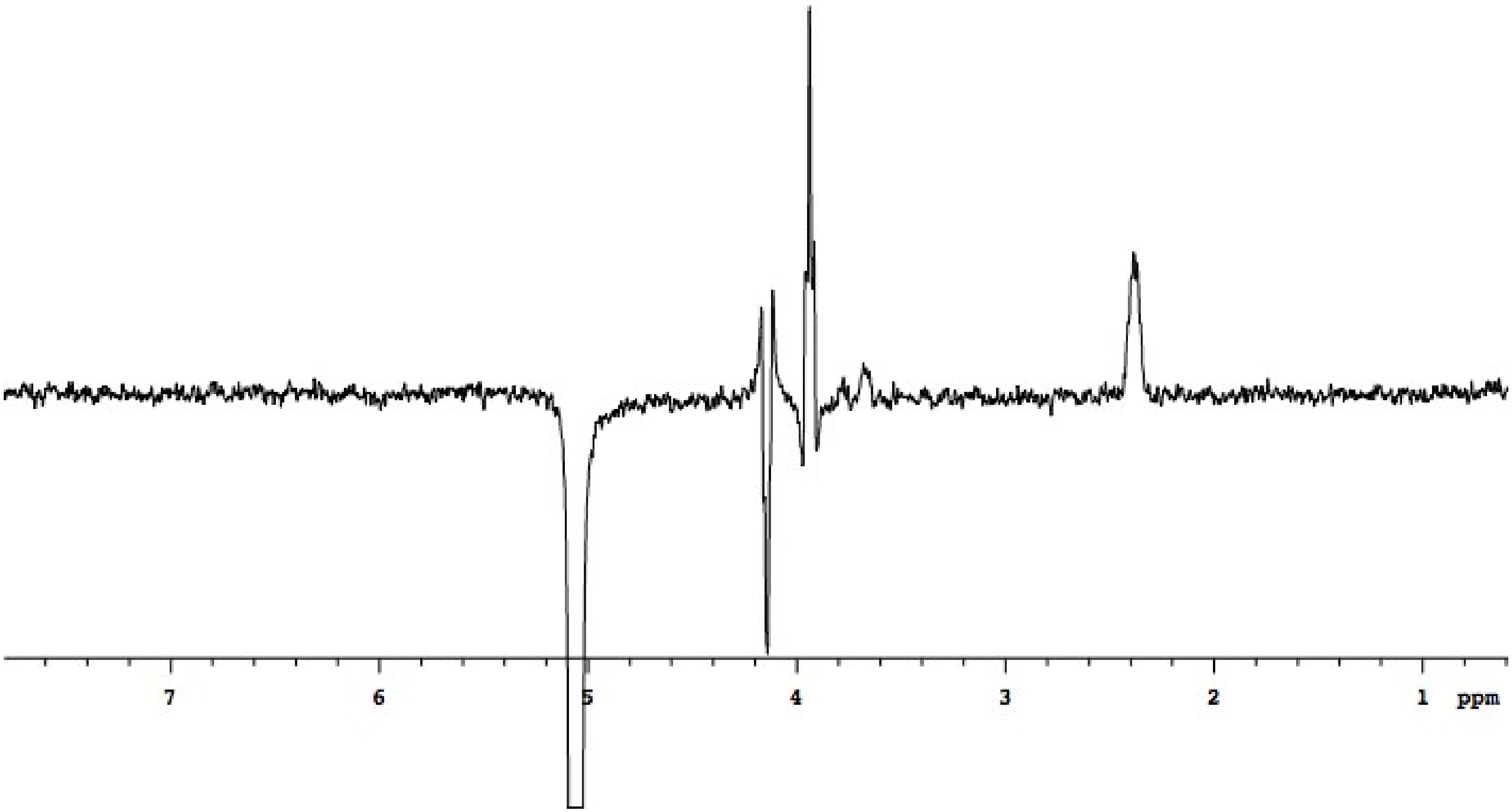

Supplement: Supplementary file 1 [file SC-009-C7SC01996C-s001.pdf]
